# Development of the AD*F*ICE_IT Models for Predicting Falls and Recurrent Falls in Community-Dwelling Older Adults: Pooled Analyses of European Cohorts With Special Attention to Medication

**DOI:** 10.1093/gerona/glac080

**Published:** 2022-04-04

**Authors:** Bob van de Loo, Lotta J Seppala, Nathalie van der Velde, Stephanie Medlock, Michael Denkinger, Lisette CPGM de Groot, Rose-Anne Kenny, Frank Moriarty, Dietrich Rothenbacher, Bruno Stricker, André Uitterlinden, Ameen Abu-Hanna, Martijn W Heymans, Natasja van Schoor

**Affiliations:** Amsterdam UMC location Vrije Universiteit Amsterdam, Epidemiology and Data Science, De Boelelaan 1117, Amsterdam, Netherlands; Amsterdam UMC location University of Amsterdam, Internal Medicine, Section of Geriatric Medicine, Meibergdreef 9, Amsterdam, Netherlands; Amsterdam Public Health research institute, Amsterdam, The Netherlands; Amsterdam UMC location University of Amsterdam, Internal Medicine, Section of Geriatric Medicine, Meibergdreef 9, Amsterdam, Netherlands; Amsterdam Public Health research institute, Amsterdam, The Netherlands; Amsterdam UMC location University of Amsterdam, Internal Medicine, Section of Geriatric Medicine, Meibergdreef 9, Amsterdam, Netherlands; Amsterdam Public Health research institute, Amsterdam, The Netherlands; Amsterdam Public Health research institute, Amsterdam, The Netherlands; Amsterdam UMC location University of Amsterdam, Department of Medical Informatics, Meibergdreef 9, Amsterdam, Netherlands; Institute for Geriatric Research, Ulm University at Agaplesion Bethesda Clinic, Ulm, Germany; Geriatric Center Ulm, Ulm, Germany; Division of Human Nutrition and Health, Wageningen University, Wageningen, The Netherlands; TILDA, Department of Medical Gerontology, Trinity College, Dublin, Ireland; Division of Human Nutrition and Health, Wageningen University, Wageningen, The Netherlands; School of Pharmacy and Biomolecular Sciences, Royal College of Surgeons in Ireland, Dublin, Ireland; Institute of Epidemiology and Medical Biometry, Ulm University, Ulm, Germany; Department of Epidemiology, Erasmus University Medical Center, Rotterdam, The Netherlands; Department of Epidemiology, Erasmus University Medical Center, Rotterdam, The Netherlands; Department of Internal Medicine, Erasmus University Medical Center, Rotterdam, The Netherlands; Amsterdam Public Health research institute, Amsterdam, The Netherlands; Amsterdam UMC location University of Amsterdam, Department of Medical Informatics, Meibergdreef 9, Amsterdam, Netherlands; Amsterdam UMC location Vrije Universiteit Amsterdam, Epidemiology and Data Science, De Boelelaan 1117, Amsterdam, Netherlands; Amsterdam Public Health research institute, Amsterdam, The Netherlands; Amsterdam UMC location Vrije Universiteit Amsterdam, Epidemiology and Data Science, De Boelelaan 1117, Amsterdam, Netherlands; Amsterdam Public Health research institute, Amsterdam, The Netherlands

**Keywords:** Accidental falls, Fall-risk-increasing drugs, Individual participant data, Prognosis

## Abstract

**Background:**

Use of fall prevention strategies requires detection of high-risk patients. Our goal was to develop prediction models for falls and recurrent falls in community-dwelling older adults and to improve upon previous models by using a large, pooled sample and by considering a wide range of candidate predictors, including medications.

**Methods:**

Harmonized data from 2 Dutch (LASA, B-PROOF) and 1 German cohort (ActiFE Ulm) of adults aged ≥65 years were used to fit 2 logistic regression models: one for predicting any fall and another for predicting recurrent falls over 1 year. Model generalizability was assessed using internal–external cross-validation.

**Results:**

Data of 5 722 participants were included in the analyses, of whom 1 868 (34.7%) endured at least 1 fall and 702 (13.8%) endured a recurrent fall. Positive predictors for any fall were: educational status, depression, verbal fluency, functional limitations, falls history, and use of antiepileptics and drugs for urinary frequency and incontinence; negative predictors were: body mass index (BMI), grip strength, systolic blood pressure, and smoking. Positive predictors for recurrent falls were: educational status, visual impairment, functional limitations, urinary incontinence, falls history, and use of anti-Parkinson drugs, antihistamines, and drugs for urinary frequency and incontinence; BMI was a negative predictor. The average *C*-statistic value was 0.65 for the model for any fall and 0.70 for the model for recurrent falls.

**Conclusion:**

Compared with previous models, the model for recurrent falls performed favorably while the model for any fall performed similarly. Validation and optimization of the models in other populations are warranted.

Falls in community-dwelling older adults are common and form a growing public health problem in aging societies. Approximately one third of adults aged 65 years and older living in the community endure a fall every year and almost half of fallers experience a repeated fall within the next year (1). One out of 5 falls results in severe injury requiring medical attention, such as a hip fracture or head injury (2). Other consequences of falls include functional decline, avoidance of physical and social activities, mortality, and high costs for society (2–4).

Efficient and cost-effective implementation of fall preventive strategies requires identification of individuals most at risk of falls. The strongest risk factors for falls include history of falls, gait problems, dizziness, the use of a walking aid, and Parkinson disease (5). In addition, use of certain medications is increasingly recognized as an important risk factor for falls. Fall-risk increasing-drugs (FRIDs) include among others loop diuretics, antidepressants, and antiepileptics (6–8). A growing number of studies have described the development of prediction models for falls that combine data for multiple predictors to estimate the probability of a future fall incident. However, in a recent systematic review of prediction models for falls in community-dwelling older adults, researchers concluded that no model can currently be recommended for practice as all models suffered from a high risk of bias (9). This was caused by restrictive eligibility criteria resulting in the exclusion of participants with specific diseases or conditions and limitations with regard to statistical methods and outcome assessments.

Use of certain medications is recognized as an important risk factor for falls and taking medication use into account may therefore help predict falls (6–8). However, previous studies describing the development of prediction models for falls have generally considered only a limited number or no variables related to use of FRIDs in model development (9). Moreover, previous studies have not considered possible variations in the relative strength of predictors in different medication user groups which may arise due to differences in patient characteristics. There is a need for tools to help distinguish between high- and low-risk patients within medication user groups (10). Such tools may aid in clinical decision-making regarding the deprescribing of FRIDs.

Inclusion of a high number of variables in model development typically requires larger data sets to minimize the risk of model overfitting (11). In one study, researchers developed a prediction model for falls using a wide range of candidate predictors that included medication use (12). However, the researchers used a relatively small sample of 976 participants. In another study, a set of prediction models for falls and recurrent falls was developed using data from 6 056 Medicare enrollees (13). While the researchers used a large sample for the development of the models, they did not consider medication use for inclusion in the models. Other studies have used large data sets comprised of routinely collected data, such as electronic health records or insurance claims (14–19). However, routinely collected data may suffer from misclassification bias and underreporting as a result of the data not being collected for the purpose of research (20). Indeed, prediction models based on routinely collected data may be less sensitive to noninjurious falls as these typically go unreported (21). A pooled analysis of individual participant data from multiple studies would allow prediction models to be developed using a large data set containing a wide spectrum of candidate fall predictors. As an added benefit, prediction models derived from pooled analyses have the potential of being generalizable to a wider range of populations (22).

The present work reports on the rigorous development and internal validation of the AD*F*ICE_IT models for predicting any fall and recurrent falls over a 1-year period in community-dwelling older adults. Our main aim was to improve upon previous prediction models for falls in terms of predictive performance by drawing upon a large, pooled sample of European cohorts and by considering a wide range of candidate predictors, including medication use. Secondary aims of this paper were to (a) develop an additional set of models using only variables that are easily obtainable in clinical practice, (b) explore differences with respect to the selected predictors in different groups of medication users, and (c) examine whether a prediction model derived from a larger, retrospective data set would result in better discriminative performance.

## Method

The Transparent Reporting of a multivariable prediction model for Individual Prognosis Or Diagnosis (TRIPOD) checklist was used as a guideline for reporting this study (Supplementary File 1: Cohorts and TRIPOD checklist) (11).

### Study Population

This study draws on data from European cohorts that were combined using harmonization procedures as part of the larger AD*F*ICE_IT project (23). The AD*F*ICE_IT harmonized cohort data set comprises 6 cohort studies, which all collected retrospective data on falls and among which 3 cohorts also collected prospective data on fall incidents. For the main analyses, we used baseline and 1-year follow-up data from the 3 cohorts with prospective data on falls, consisting of the Longitudinal Aging Study Amsterdam (LASA; wave C, 1995/1996), the B-vitamins for the PRevention Of Osteoporotic Fractures study (B-PROOF), and the Activity and Function in the Elderly in Ulm study (ActiFE Ulm) (24–26). For the main analyses, we included 5 722 participants aged 65 years and older from these cohorts for whom medication and follow-up data were available. In an additional analysis (see “Additional Analyses”), we also included baseline data from the remaining 3 cohorts with only retrospective data on fall incidents, that is, LASA (wave 3B, 2012/2013), the Rotterdam Study, and the Irish Longitudinal Study on Ageing (TILDA) (24,27,28). For details regarding all cohorts in the AD*F*ICE_IT harmonized cohort data set, we refer to Supplementary File 1. Participants from all cohorts provided informed consent, and all cohort studies were approved by their institutional ethics committees.

### Harmonization

We developed harmonization algorithms for variables measuring the same concept across at least 2 of the 3 cohorts with prospective data on falls. These are presented in the harmonization guide (Supplementary File 2).

### Outcomes

The ascertainment and definition of falls in the cohorts with prospective data on falls conformed with the recommendations of the ProFaNE statement (29). Falls were measured prospectively using falls calendars for 12 months. Participants were asked to record falls every week and to return the calendar at 3-month intervals. A fall was defined as an unintentional change in position resulting in coming to rest at a lower level or ground. Participants were contacted by telephone if the calendars were not returned or if the calendars were filled out incorrectly. Two outcome variables were defined: any fall (1 or more falls) and recurrent falls (2 or more falls) during a 1-year follow-up period.

### Candidate Predictors

A total of 82 candidate predictors were selected based on previously reported risk factors of falls (5–8,30). Candidate predictors were measured at baseline and included: sociodemographic variables; measures of emotional, cognitive, and physical functioning; self-reported chronic conditions; variables related to lifestyle; biomarkers; and use of certain medications. Additionally, a cohort index was added as a candidate predictor to account for possible differences in baseline risk between the cohort populations. This approach has been recommended for increasing model generalizability in a pooled analyses of a low number of studies (22). Included medication groups consisted of potential FRIDs as identified by recent reviews (6–8,31–34). Medication use was coded using the Anatomical Therapeutic Chemical classification system.

### Missing Data

Within each cohort with prospective data on falls, there were at least 2 and a maximum of 11 predictors that contained missing values for all individuals and were thus systematically missing (see Supplementary File 3: Table 1 for a complete overview of which variables were systematically missing). For example, verbal fluency was systematically missing for LASA while visual impairment, number of functional limitations, and urinary incontinence were systematically missing for B-PROOF. Multiple imputation by chained equations was employed to impute missing values for the predictors and outcome variables. We imputed 5 data sets for each outcome variable. We included the cohort index in the imputation models, which is a valid approach to account for the between-study heterogeneity (35).

### Model Development, Internal Validation, and Updating

Logistic regression was employed to develop 2 separate prediction models: one for predicting any fall and another for predicting recurrent falls. A backward elimination procedure with a criterion of *p* < .05 was applied to reduce the number of predictors in both models (36). *p* Values were derived from the Wald statistic as calculated from the multiply imputed data using Rubin’s rules (37). For the continuous predictors grip strength, verbal fluency, depression score, physical activity, functional limitations, processing speed, and gait speed *Z*-score transformations were applied for the main purpose of harmonization. The other continuous variables were modeled on their original scale. Nonlinearity between the continuous variables and outcome variables was visually inspected using LOESS plots. We found that the use of restricted cubic splines for nonlinear variables made no difference with respect to the predicted risks and model performance and we therefore chose to model these variables linearly.

Generalizability of the prediction models was assessed using internal–external cross-validation. Internal–external cross-validation allows the predictive performance of the prediction models to be assessed across all 3 cohorts while allowing for the final prediction models to be built using all data (22). The internal–external cross-validation procedure consisted of the following steps: (a) Two of the 3 cohorts were selected as derivation data. The remaining study served as the validation data set; (b) A prediction model was developed using the derivation data. We applied the same modeling methods as for the original model with the exclusion of the cohort index; (c) The validation data set was used to evaluate the performance of the derived model; and (d) Steps a–c were repeated until each cohort had been used as a validation data set.

Performance of the prediction models was evaluated in terms of calibration and discrimination (11). Calibration was assessed using calibration plots and by calculating the calibration intercept and slope. The calibration slope assesses the agreement between estimated risks and observed outcomes. The calibration slope has a target value of 1: a value of <1 suggests that estimated risks are too high for individuals who are at high risk and too low for those who are at low risk. Conversely, a slope >1 suggests that risk estimates are too moderate. The calibration intercept quantifies the calibration-in-the-large and has a target value of 0. A negative calibration intercept indicates overestimation of risk, whereas a positive value suggests underestimation of risk. Discrimination was measured using the *C*-statistic, where a value of 0.5 indicates no discrimination and a value of 1 indicates perfect discrimination. The *C*-statistic can be interpreted as the probability that a randomly selected participant from the event group has a higher predicted probability of having the event than a randomly selected participants from the nonevent group. *C*-statistic measures derived from the internal–external cross-validation procedure were averaged to obtain a single estimate.

A common problem in prediction research is overfitting, which can result in overestimation of the coefficients. To address the possibility of overfitting, we adjusted the coefficients for each prediction model using a shrinkage procedure. We obtained a shrinkage factor for each model by averaging the calibration slope values as derived from the internal–external cross-validation procedure for that model. The shrinkage factor was then multiplied with the coefficients of the respective prediction model. Finally, we reestimated the intercept for each model so that the average predicted risk was equal to the observed event rate.

### Additional Analyses

We conducted 3 additional analyses. First, we developed another set of models for predicting any fall and recurrent falls using only candidate predictors that are easily obtainable in clinical practice. Specifically, we excluded the following predictors from the original selection of candidate predictors: verbal fluency score, processing speed score, Hospital Anxiety and Depression Scale-Anxiety (HADS-A) score, total physical activity, immediate recall, delayed recall, estimated glomerular filtration rate, C-reactive protein, vitamin B12, and vitamin D. The models were developed and validated using the same strategy as in the main analysis.

Second, we explored whether there were differences with respect to the selected predictors across different groups of medication users. We hypothesized that variations in population characteristics between these subgroups could affect the relative strength of predictors and hence the predictors that are selected. Using the cohorts with prospective data on falls, we developed and validated prediction models for any future fall within user groups of commonly prescribed potential FRIDs that were used by at least 800 of the participants, that is, user groups of the following medications: ACE inhibitors, low ceiling diuretics, beta-blockers, proton pump inhibitors, and statins (6–8,31–34). The models were developed using the same model development strategy as for the main analysis. For each model, we obtained an optimism-adjusted *C*-statistic estimate via a bootstrap procedure using 200 samples.

Finally, we examined whether a prediction model derived from an even larger population would result in better discriminative performance. To this end, we conducted a pooled analysis of all 6 cohorts within the AD*F*ICE_IT harmonized cohort data set, namely LASA (wave C and wave 3B; *n* = 1 507 and *n* = 887, respectively), B-PROOF (*n* = 29 12), the Rotterdam Study (*n* = 7 151), ActiFE Ulm (*n* = 1 463), and TILDA (*n* = 8 081). All participants aged 50 years and older with medication data were included in this analysis. History of 1 or more falls in the past 12 months was used as outcome variable. The model was developed using the same model development strategy as for the prospective analyses. Discriminative performance of the model was evaluated using internal–external cross-validation.

### Software

Statistical analyses were performed using the R (version 4.0.2) statistical programming language. We used the “mice” package for multiple imputation and the “psfmi” package for the backward elimination, bootstrapping, and shrinkage procedures (38,39).

## Results

### Study Participants

A total of 22 001 participants were included in the analyses, of which 5 722 participants with prospective data were included in development of the prediction models for future falls and recurrent falls. Within the 1-year follow-up, 1 868 (34.7%) participants endured at least 1 fall and 702 (13.3%) endured at least 2 falls. The main characteristics of the cohorts with prospective data on falls are presented in Table 1 (see Supplementary File 3: Table 1 for a complete overview). Participants in the cohorts with prospective data on falls differed with respect to all characteristics (*p* < .05), except for history of at least 1 fall in the previous 12 months. In comparison with participants from LASA and B-PROOF, participants from ActiFE Ulm were less often female, lower educated, and found to have higher grip strength scores as well as lower systolic blood pressure. The characteristics of all cohorts with retrospective data on falls are presented in Supplementary File 3: Table 2.

**Table 1.** Baseline Characteristics of the Older Adults From the European Cohorts With Prospective Data on Falls

Variable	LASA (*n* = 1 433)	B-PROOF (*n* = 2 912)	ActiFE Ulm (*n* = 1 377)	Total (*n* = 5 722)	*p* Value
Age (years)	75 [69, 81]	73 [69, 78]	74 [70, 81]	74 [69, 79]	<.001
Sex, female	738 (51.5)	1 456 (50.0)	591 (42.9)	2 785 (48.7)	<.001
Educational status					<.001
Low	1 038 (72.5)	2 006 (68.9)	1 069 (78.5)	4 113 (72.1)	
Middle	219 (15.3)	149 (5.1)	141 (10.4)	509 (8.9)	
High	174 (12.2)	755 (25.9)	151 (11.1)	1 080 (18.9)	
Depressive symptoms*	212 (15.3)	132 (4.6)	140 (10.7)	484 (8.7)	<.001
Animals or items named in verbal fluency test	―	12 [9, 15]	21 [17, 25]	17 [12, 23]	<.001
BMI	26.93 ± 4.28	27.14 ± 3.96	27.56 ± 4.12	27.19 ± 4.08	<.001
Visual impairment	102 (7.1)	―	201 (14.8)	303 (10.9)	<.001
Number of functional limitations (0–5)	1 [0, 2]	―	0 [0, 2]	1 [0, 2]	<.001
Grip strength (kg)	29.23 ± 10.18	32.49 ± 10.84	33.51 ± 11.37	31.91 ± 10.93	<.001
Urinary incontinence	359 (25.1)	―	511 (37.6)	870 (31.2)	<.001
Systolic blood pressure (mmHg)	153.08 ± 23.91	148.73 ± 19.68	142.34 ± 11.81	148.20 ± 19.67	<.001
≥1 fall in previous 12 months	458 (32.0)	737 (32.6)	469 (34.5)	1 664 (33.0)	.348
≥2 falls in previous 12 months	215 (15.1)	268 (11.9)	157 (11.6)	640 (12.7)	.006
Current smoker	265 (18.5)	281 (9.6)	88 (6.4)	634 (11.1)	<.001
Anti-Parkinson drugs	6 (0.4)	38 (1.3)	24 (1.7)	68 (1.2)	.004
Antiepileptics	17 (1.2)	52 (1.8)	39 (2.8)	108 (1.9)	.005
Drugs for urinary frequency and incontinence	7 (0.5)	40 (1.4)	29 (2.1)	76 (1.3)	.001
Antihistamines	16 (1.1)	83 (2.9)	17 (1.2)	116 (2.0)	<.001

*Notes:* ActiFE Ulm = Activity and Function in the Elderly in Ulm study; BMI = body mass index; B-PROOF = B-vitamins for the PRevention Of Osteoporotic Fractures study; LASA = Longitudinal Aging Study Amsterdam; *SD* = standard deviation. Data are presented as mean ± *SD*, *n* (%), or median [interquartile range]. The sign “―” indicates the corresponding variable is systematically missing.

*Defined by validated cutoff scores for Center for Epidemiological Studies Depression Scale (in LASA), Hospital Anxiety and Depression Scale-Depression subscale (in ActiFE Ulm), and Geriatric Depression Scale (in B-PROOF).

*p* Values were calculated using chi-square tests, Kruskal–Wallis tests, and ANOVA tests.

### Model Development and Validation

#### AD*F*ICE_IT model for predicting any fall

After applying the backward elimination procedure in the 3 cohorts with prospective data on falls, 12 predictors remained significant in the final AD*F*ICE_IT model for predicting any fall (Table 2). The model included the following predictors with a positive relationship with any fall: educational status, depression score, verbal fluency score, number of functional limitations, history of at least 1 fall in the previous 12 months, history of at least 2 falls in the previous 12 months, use of antiepileptics, and use of drugs for urinary frequency and incontinence. The following predictors showed a negative relationship with any fall: body mass index (BMI), grip strength, systolic blood pressure, and smoking status.

**Table 2.** Final AD*F*ICE_IT Models for Predicting Any Fall and Recurrent Falls in Community-Dwelling Older Adults Derived From the European Cohorts With Prospective Data on Falls

	Model for Predicting Any Fall		Model for Predicting Recurrent Falls	
Predictor	Beta^§^	OR (95% CI)^§^	Beta^†^	OR (95% CI)^†^
Intercept	0.062		−1.732	
Educational status				
Middle	0.127	1.14 (0.93–1.40)	0.080	1.08 (0.79–1.49)
High	0.283	1.33 (1.14–1.55)*	0.452	1.57 (1.28–1.93)*
Depression score^‡^	0.080	1.08 (1.01–1.17)		
Verbal fluency score^‡^	0.134	1.14 (1.04–1.26)*		
Visual impairment			0.418	1.52 (1.11–2.08)*
BMI	−0.020	0.98 (0.97–1.00)*	−0.032	0.97 (0.95–0.99)*
Number of functional limitations (0–5)^‡^	0.141	1.15 (1.05–1.27)*	0.242	1.27 (1.16–1.40)*
Grip strength (kg)^‡^	−0.139	0.87 (0.81–0.93)*		
Urinary incontinence			0.327	1.39 (1.15–1.68)*
Systolic blood pressure (mmHg)	−0.003	1.00 (0.99–1.00)		
≥1 fall in previous 12 months	0.432	1.54 (1.33–1.78)*	0.570	1.77 (1.42–2.20)*
≥2 falls in previous 12 months	0.590	1.81 (1.48–2.19)*	0.843	2.32 (1.71–3.16)*
Current smoker	−0.252	0.78 (0.64–0.95)*		
Use of anti-Parkinson drugs			0.669	1.95 (1.04–3.66)
Use of antiepileptics	0.456	1.58 (1.02–2.45)		
Use of drugs for urinary frequency and incontinence	0.656	1.93 (1.09–3.40)	0.541	1.72 (0.96–3.09)
Use of antihistamines			0.594	1.81 (1.14–2.87)*

*Notes:* ActiFE Ulm = Activity and Function in the Elderly in Ulm study; BMI = body mass index; B-PROOF = B-vitamins for the PRevention Of Osteoporotic Fractures study; CES-D = Center for Epidemiologic Studies Depression Scale; CI = confidence interval; GDS = Geriatric Depression Scale; HADS-A = Hospital Anxiety and Depression Scale-Anxiety; LASA = Longitudinal Aging Study Amsterdam; OR = odds ratio.

^§^Coefficients were corrected for overfitting with a shrinkage factor of 0.90.

^†^Coefficients were corrected for overfitting with a shrinkage factor of 0.91.

^‡^OR refers to standardized *Z*-score, which were used for the purpose of harmonization. *Z*-scores are calculated as: *Z*-score depression_LASA_ = (CES-D score—7.980)/7.826; *Z*-score depression_B-PROOF_ = (GDS score—1.440)/1.942; *Z*-score depression_ActiFE Ulm_ = (HADS-D score—3.802)/2.899; *Z*-score verbal fluency_B-PROOF_ = (items named in test—12.132)/3.753; *Z*-score verbal fluency_ActiFE Ulm_ = (animals named in test—21.215)/6.476; *Z*-score functional limitations_LASA_ = (number of functional limitations—1.209)/1.529; *Z*-score functional limitations_ActiFE Ulm_ = (number of functional limitations—1.013)/1.362; *Z*-score grip strength_LASA_ = (kg—29.015)/10.244; *Z*-score grip strength_B-PROOF_ = (kg—32.484)/10.841; *Z*-score grip strength_ActiFE Ulm_ = (kg—33.364)/11.412.

*p < .01.

Using our internal–external cross-validation approach, we assessed the model’s discriminative ability and calibration across the cohorts. The average *C*-statistic for the model was 0.65 (Supplementary File 3: Table 3). The model performed best when validated in LASA and worst when validated in ActiFE Ulm, with *C*-statistic measures of 0.68 (95% confidence interval [CI] 0.64–0.71) and 0.61 (95% CI 0.58–0.64), respectively. The calibration plots revealed some miscalibration when the model was validated in ActiFE Ulm (Supplementary File 3: Figure 1A). Calibration-in-the-large across the 3 cohorts was excellent, with calibration intercept values ranging from −0.02 to 0.02. Calibration slope values for the model for any fall across the 3 cohorts ranged from 0.67 to 1.12 and indicated overfitting when the model was validated in B-PROOF and ActiFE Ulm.

#### AD*F*ICE_IT model for predicting recurrent falls

The final AD*F*ICE_IT model for predicting recurrent falls contained 10 predictors (Table 2). The following predictors showed a positive relationship with recurrent falls: educational status, visual impairment, number of functional limitations, urinary incontinence, history of at least 1 fall in the previous 12 months, history of at least 2 falls in the previous 12 months, use of anti-Parkinson drugs, use of drugs for urinary frequency and incontinence, and use of antihistamines. Additionally, BMI was included as a predictor with a negative relationship with recurrent falls.

Using the internal–external cross-validation procedure, we obtained an average *C*-statistic measure of 0.70 for the model (range 0.69–0.71; Supplementary File 3: Table 3). The calibration plots revealed some miscalibration when the model was validated in B-PROOF (Supplementary File 3: Figure 1B). Intercept values ranged from −0.03 to 0.11 and indicated underestimation of risk in ActiFE Ulm. Calibration slopes ranged from 0.79 to 1.01 and indicated overfitting when the model was validated in B-PROOF.

### Additional Analyses

Results of the 3 additional analyses are presented in Supplementary File 1. In the first additional analysis, we developed additional models for predicting falls and recurrent falls using only candidate predictors that are easily obtainable in clinical practice and found that these models showed similar performance as compared with our main models (Supplementary File 3: Figure 2, Tables 4 and 5). Second, we explored an approach in which we developed prediction models for any fall within groups of medication user groups with the aim of exploring possible differences in the selected predictors for these groups. The number of predictors for the different medication user groups varied between 2 and 8 predictors (Supplementary File 3: Table 6). Of the 15 predictors that were selected in 1 or more of the models, 6 predictors were included in more than 1 model. Finally, we found that the use of a larger, retrospective database resulted in a model with lower discriminative performance as compared with the models in the main model (Supplementary File 3: Tables 8 and 9).

## Discussion

At the development stage of the AD*F*ICE_IT models, we considered a wide range of candidate predictors, including medications. The final model for any fall contained 12 predictors, of which the following showed a positive relationship with any fall: educational status, depression score, verbal fluency score, number of functional limitations, history of at least 1 fall in the previous 12 months, history of at least 2 falls in the previous 12 months, use of antiepileptics, and use of drugs for urinary frequency and incontinence. The following predictors showed a negative relationship with any fall: BMI, grip strength, systolic blood pressure, and smoking status. The model for recurrent falls contained 10 predictors, of which the following showed a positive relationship with recurrent falls: educational status, visual impairment, number of functional limitations, urinary incontinence, history of at least 1 fall in the previous 12 months, history of at least 2 falls in the previous 12 months, use of anti-Parkinson drugs, use of drugs for urinary frequency and incontinence, and use of antihistamines. BMI was included as a predictor with a negative relationship with recurrent falls. The average *C*-statistic for the cohorts in the main analyses was 0.65 for the model for any fall and 0.70 for the model for recurrent falls. Calibration of both models was fair with the models showing good calibration when validated in the 2 Dutch cohorts and suboptimal calibration when validated in the German cohort.

Most predictors in the final prediction models for any fall (ie, falls history, educational status, depression, cognitive functioning, visual impairment, BMI, functional limitations, grip strength, urinary incontinence, and use of antiepileptics) were also included in previous models for predicting falls (9). Among the predictors included in the model for any fall were also systolic blood pressure and smoking status, which both showed a negative relationship with falls. Although previous studies have considered these as candidate predictors for predicting falls, they had not been included as predictors in any final models (9). Low blood pressure is a well-known risk factor for falls (40). The negative direction of the effect for smoking in the model for any fall may seem counterintuitive given that smoking is known as a risk factor for frailty and a number of chronic diseases (41). Yet 2 previous studies have also reported a negative association between smoking and fall risk, which has led some to suggest smoking status may be a marker for being able to cope better with smoking-related diseases (42,43). Verbal fluency and educational status were included as predictors with a positive relationship in the model for any fall, meaning that higher verbal fluency scores and a higher educational status were associated with a higher fall risk. This contradicts findings of other studies, which have generally suggested a protective effect of better executive functioning in general as well as better verbal fluency specifically (44,45). In addition, studies investigating the association between educational status and fall risk have reported mixed results with most studies finding no association (5). When checking the univariable associations, it was found that for all predictors, directions were similar in the univariable models as compared with the multivariable models (data not shown). Nonetheless, our results should not be interpreted as evidence for any causal effects as the predictors can be proxies for other fall risk factors. Higher verbal fluency scores and educational status may both reflect a higher socioeconomic status, which is known to be related to higher alcohol use (46,47). In addition, previous research has also indicated that older adults with higher education are generally more physically active, which could imply a greater level of exposure to activity- and environmental-based risk factors (48,49).

Almost all predictors in the model for recurrent falls (ie, falls history, educational status, visual impairment, weight, functional limitations, and urinary incontinence) have been included in previous models for recurrent falls (50–52). Predictors that had not been included in earlier models for recurrent falls were medication-related (ie, use of anti-Parkinson drugs, use of drugs for urinary frequency and incontinence, and use of antihistamines). Previous studies reporting prediction models for falls and recurrent falls have generally not considered a wide range of FRIDs as candidate predictors (9), although exceptions exist (12,18,19,53). Almost all medications included as predictors in our final models for predicting any fall and recurrent falls (ie, antiepileptics, drugs for urinary frequency and incontinence, and antihistamines) were classified as FRIDs by panelists in a recent Delphi consensus study (54). However, the panelists were not able to reach consensus for anti-Parkinson drugs, which was included as predictor in the model for recurrent falls. In addition, meta-analyses also found no consistent association between anti-Parkinson drugs and fall risk (8). The positive relationship between the use of anti-Parkinson medication and recurrent falls may be due to anti-Parkinson medication serving as a proxy for parkinsonism. In our study, we only had data on medication and not on parkinsonism.

It is worth noting that age, sex, gait speed, and balance were not included as predictors in the final models in the main analyses, even though these variables are among the most frequently selected predictors in other prediction models for falls (9). This may be explained by the fact that we included a wide range of candidate predictors, including predictors not often used such as potential FRIDs and verbal fluency. Interestingly, when we removed verbal fluency as a candidate predictor in an additional analysis, it resulted in the inclusion of gait speed, fear of falling, and use of calcium channel blockers. This suggests these predictors have less predictive value when modeled together with verbal fluency.

The obtained *C*-statistic value for the model for any fall lies with the range of previous prediction models that have been validated, that is 0.62 and 0.69 (9), and our model for recurrent falls performs favorably as compared with earlier models. As in our study, other studies have also found prediction models for recurrent falls to perform better in terms of discrimination as compared with prediction models for any fall (12,13,55). In comparison to a single fall, a recurrent fall is more likely to be due to an underlying risk factor as opposed to chance alone (5), which makes recurrent falls easier to predict.

The internal–external cross-validation procedure revealed some heterogeneity with regard to the calibration and discriminative performance of the AD*F*ICE_IT models for predicting any fall and recurrent falls across cohorts. For both models, the lowest *C*-statistic values were obtained for the German ActiFE Ulm study as compared with the Dutch LASA and B-PROOF cohorts. The heterogeneity in performance may be attributable to differences in population and geographical setting or variations in study procedures between the cohorts. Overall, participants from ActiFE Ulm appeared healthier in comparison with those from LASA and B-PROOF. On average, they had higher grip strength scores as well as lower blood pressure, which may partly be due to the lower proportion of female participants in ActiFE Ulm. The cohorts also used different tests for assessing a number of variables, which we handled by using harmonization algorithms and *Z*-score transformations.

Differences in performance of the models across the cohorts may also be attributed to heterogeneity in odds ratios for predictors of falls. In general, studies into risk factors for falls have shown a substantial amount of inconsistency in terms of reported odds ratios for risk factors (5). In a validation study of another prediction model for falls, researchers found univariable associations between common risk factors and falls to be weaker in ActiFE Ulm than in the other cohorts in their analyses (56). When validating their model in ActiFE Ulm, the researchers also found the calibration of their model to be suboptimal and they obtained a lower *C*-statistic value (*C*-statistic = 0.56) in comparison to the other cohorts in their analyses.

We conducted 3 additional analyses. First, we developed an additional model for predicting any fall using only candidate predictors easily obtainable in clinical practice. Performance of this model was comparable to that of the AD*F*ICE_IT model for predicting any fall. The model has potential as a practical screening tool in clinical settings. However, given the observed variation in performance of the model in the 3 cohorts, external validation and further optimization of the model in populations outside the Netherlands is needed. As part of the larger AD*F*ICE_IT project, we have implemented this model in a clinical decision support system for optimizing FRIDs deprescribing in falls clinic patients, which we will evaluate in a multicenter trial across falls clinics in the Netherlands (23).

In the second additional analysis, we developed models for predicting falls within user groups of commonly prescribed potential FRIDs to explore possible differences in the selected predictors in groups of medication users. There were large differences between the prediction models for the 5 medication user groups with respect to the predictors in the final models, which may have arisen from variations in population characteristics between the groups. Indeed, of the 15 predictors selected in 1 or more of the models, only 6 predictors were included in more than 1 model, that is, educational status, able to perform tandem stand, history of at least 1 fall, history of at least 2 falls, use of calcium channel blockers, and use of antiepileptics. A limitation of this analysis was the lower sample sizes available for developing the models, which may have resulted in some model instability. Nonetheless, these analyses were merely explorative in nature. As post hoc analyses, we compared the discriminative performance of the respective subgroup models with that of the AD*F*ICE_IT main model for predicting any fall, when applied in the subgroup samples. In comparison with the subgroup models, the AD*F*ICE_IT model performed very similarly and in some cases even better (Supplementary File 3: Table 7). All in all, our results indicate that while the relative strength of predictors may vary across medication user groups, the performance of the AD*F*ICE_IT model was stable across these subgroups.

In the third additional analysis, we examined whether a pooled analysis of a retrospective data set comprised of 6 cohorts could yield a model with better discriminative properties when compared with the models in the main analyses. The final model in this analysis did not outperform the prediction models in the main analyses in terms of discriminative performance (range *C*-statistic: 0.60–0.67). Importantly, we observed a considerable degree of heterogeneity with respect to model performance across the 6 cohorts when validating the model. This further illustrates that models for predicting fall-related outcomes may show variations in performance between different populations. The final model included most of the predictors included in the models from the main analyses. Differences in predictors may be attributable to the cross-sectional design, differences in sample size, and the inclusion of different study cohorts.

Strengths of this study include the use of a large pooled data set, which allowed us to develop and internally validate our models across multiple settings and to include a wide range of candidate predictors, including medications. A further strength of this study is that falls were prospectively measured on a weekly basis using fall calendars. Additionally, our works adheres to the reporting guidelines for prediction models outlined in the TRIPOD Statement (11). A few limitations deserve consideration. First, the cohorts used different tests for assessing a number of variables, which may have contributed to the differences in population characteristics between the cohorts. We applied harmonization algorithms and *Z*-score transformations on cohort-level to mitigate this source of heterogeneity. The *Z*-score transformations also allow the final models to be validated in future studies in which other tests and instruments were used for measuring these variables. For example, data from other instruments for measuring activities of daily living, such as the Katz-score, can be used as input for the functional limitations predictor. A disadvantage of using harmonization algorithms is that some variables had to be reduced to simpler variables, resulting in some loss of information. Second, although we included a wide range of candidate predictors in our analyses, we were unable to consider the role of genetic variation and extrinsic factors (eg, environmental and housing factors) in the development of our models. Future research could investigate whether our models can be improved by considering these variables. Recent publications have found associations between genetic variation and fall risk and have suggested genetic variation can act as effect modifier in the relationship between medication and fall risk (57,58).

## Conclusion

The AD*F*ICE_IT models include well-known predictors for falls as well as some predictors that have not been included in previous models, namely use of drugs for urinary frequency and incontinence, use of antihistamines, use of anti-Parkinson drugs, smoking status, and systolic blood pressure. Compared with earlier models, the model for recurrent falls showed favorable performance in terms of discrimination while the model for any fall performed similarly in terms of discrimination. However, performance of the models differed across the cohorts and therefore external validation and further optimization of the models in other populations outside the Netherlands is warranted.

## Supplementary Material

glac080_suppl_Supplementary_Material
