# Supplementary material for: Development of the ADFICE_IT Models for Predicting Falls and Recurrent Falls in Community-Dwelling Older Adults: Pooled Analyses of European Cohorts With Special Attention to Medication
Source: J Gerontol A Biol Sci Med Sci. 2022 Apr 4;77(7):1446–54. doi: 10.1093/gerona/glac080 (PMC9255686; doi:10.1093/gerona/glac080)
Supplement: glac080_suppl_Supplementary_Material [file glac080_suppl_supplementary_material.pdf]

Supplementary Files

Contents

Supplementary File 1: Used Cohort Studies 2
Supplementary File 1: Results Additional Analyses 4
Supplementary File 1: TRIPOD Checklist 5
Supplemental file 2: data harmonization guide 8
Supplementary File 3: Table 1 99
Supplementary File 3: Table 2 104
Supplementary File 3: Table 3 111
Supplementary File 3: Figure 1 112
Supplementary File 3: Table 4 113
Supplementary File 3: Table 5 114
Supplementary File 3: Figure 2 115
Supplementary File 3: Table 6 116
Supplementary File 3: Table 7 118
Supplementary File 3: Table 8 119
Supplementary File 3: Table 9 121

Supplementary File 1: Used Cohort Studies

LASA is an ongoing cohort study that focuses on the determinants, trajectories and consequences of physical, cognitive, emotional and social functioning in older adults in the Netherlands. Data collection started in 1992-1993 [1]. A sample of participants aged 55 years and over was drawn from eleven municipalities across three regions. Sampling was performed at random and stratified by age, sex, urbanization grade and expected 5-years mortality rate. For the main analyses, we used data from wave C (1995/1996) which had 1509 participants, aged 65 years and older as of January 1, 1996. Of these, we included 1433 participants in the main analyses for whom follow-up and medication data was available.

B-PROOF is a multicenter, randomized, placebo-controlled, double-blind trial investigating the efficacy of vitamin B12 and folic acid supplementation on the prevention of fracture-incidence in individuals aged 65 years and over with an elevated plasma homocysteine concentration [2]. Additional inclusion criteria were: compliance to tablet intake in a period prior to start of the trial and competence to make own decisions. Participants that were bedridden or wheelchair bound, that were diagnosed with cancer, or that had high serum creatinine levels were excluded. A total of 2919 participants were recruited across three centers in the Netherlands between 2008 and 2011. Results of B-PROOF showed no effect of the intervention on the time to first or second fall and the number of falls experienced during follow-up [3]. Therefore, we treated B-PROOF as a prospective cohort study in the context of the present paper. For the main analyses, we used baseline and one-year follow-up data of 2912 participants for whom medication and follow-up data was available.

ActiFE Ulm is a German population-based cohort study among community-dwelling people living in the greater Ulm, Neu-Ulm and Alb-Donau-Kreis areas. Recruitment started in 2009 and finished in 2010 [4]. Inclusion criteria for: age between 65 and 90 years, could provide informed consent, and ability to walk through their own room. People with severe deficits in cognition, vision or hearing were excluded from participation. A total of 1506 participants were sampled randomly and according to sex and age strata. We included baseline and one-year follow-up data from 1366 participants in the main analyses for whom follow-up and medication data was available.

For details regarding the three cohorts with only retrospective data on fall incidents we refer to their respective cohort profiles [1,5,6]. In brief, wave 3B of LASA was conducted between 2012 and 2013. The Rotterdam Study is an ongoing Dutch, prospective cohort study that started in 1990. The harmonized cohort dataset included data from measurements conducted between 2009 and 2014, which were collected as part of the fifth examination cycle of the first cohort, the third examination cycle of the second cohort and the second examination cycle of the third cohort. TILDA is an ongoing cohort study in Ireland. The harmonized cohort dataset contained data from the baseline measurements conducted between 2009 and 2011. Participants from all cohorts in the AD*F*ICE_IT harmonized cohort dataset provided informed consent and all cohort studies were approved by their institutional ethics committees.

References

1. Hoogendijk EO, Deeg DJH, Breij S De, Klokgieters SS, Kok AAL. The Longitudinal Aging Study Amsterdam: cohort update 2019 and additional data collections. *Eur J Epidemiol*. 2019;(0123456789). doi:10.1007/s10654-019-00541-2
2. Wijngaarden V, Van Wijngaarden JP, Dhonukshe-Rutten RA, et al. Rationale and design of the B-PROOF study, a randomized controlled trial on the effect of supplemental intake of vitamin B12 and folic acid on fracture incidence Rationale and design of the B-PROOF study, a randomized controlled trial on the effect of sup. *BMC Geriatr*. 2011;11(December). doi:10.1186/1471-2318-11-80
3. van Wijngaarden JP, Swart KMA, Enneman AW, et al. Effect of daily vitamin B-12 and folic acid supplementation on fracture incidence in elderly individuals with an elevated plasma homocysteine concentration: B-PROOF, a randomized controlled trial. *Am J Clin Nutr*. 2014;100(6):1578-1586. doi:10.3945/ajcn.114.090043
4. Denkinger MD, Franke S, Rapp K, et al. Accelerometer-based physical activity in a large observational cohort - Study protocol and design of the activity and function of the elderly in Ulm (ActiFE Ulm) study. *BMC Geriatr*. 2010;10. doi:10.1186/1471-2318-10-50
5. Donoghue OA, McGarrigle CA, Foley M, Fagan A, Meaney J, Kenny RA. Cohort profile update: The Irish Longitudinal study on ageing (TILDA). *Int J Epidemiol*. 2018;47(5):1398-1398L. doi:10.1093/ije/dyy163
6. Ikram MA, Brusselle G, Ghanbari M, et al. Objectives, design and main findings until 2020 from the Rotterdam Study. *Eur J Epidemiol*. 2020;35(5):483-517. doi:10.1007/s10654-020-00640-5

Supplementary File 1: Results Additional Analyses

A number of additional analyses were conducted. First, we developed an additional set of models for predicting any fall and recurrent falls using only candidate predictors that are easily obtainable in clinical practice. The model for predicting any fall developed using this subset of candidate predictors contained 14 predictors, which largely overlap with those included in the model for predicting any fall in the main analysis (Supplementary Table 4). In comparison with the model in the main analysis, the model contained the predictors gait speed, fear of falling, and use of calcium channel blockers instead of verbal fluency. With an average C-statistic of 0.65 (range 0.61-0.67), the discriminative performance of the model was similar to that of the model for any fall in the main analysis (Supplementary Table 5). Visual inspection of the calibration plot for the model showed observed and predicted risks largely overlapped in the three cohorts (Supplementary Figure 2). The final model for predicting recurrent falls contained the same 10 predictors as the final model for recurrent falls in the main analysis (results not shown).

Second, we explored an approach in which we developed prediction models for any fall within groups of medication user groups with the aim of exploring possible differences in the selected predictors for these groups. The number of predictors for the different medication user groups varied between 2 and 8 predictors. Of the 15 predictors that were selected in one or more of the models, 6 predictors were included in more than one model, i.e., educational status, able to perform tandem stand, history of at least one fall, history of at least two falls, use of calcium channel blockers, and use of antiepileptics. The final models are presented in Supplementary Table 6. Discriminative performance of the models varied with optimism-adjusted C-statistic measures ranging from 0.61 to 0.69 (Supplementary Table 7).

Finally, we examined whether a pooled analysis of a larger retrospective dataset comprised of all six cohorts within the harmonized cohort dataset could yield a model with better discriminative properties. After the backward elimination procedure, 26 predictors remained significant in relation to a fall in the past year. Among these 26 predictors were 8 variables that were also included as a predictor in the models in the main analyses (Supplementary Table 8). Discriminative performance of the model across the six cohorts was lower when compared to the models in the main analyses with an average C-statistic of 0.64 (range 0.60-0.67; Supplementary Table 9).

Supplementary File 1: TRIPOD Checklist

Section/Topic	Item	Checklist Item	
**Title and abstract**			
Title	1	Identify the study as developing and/or validating a multivariable prediction model, the target population, and the outcome to be predicted.	✓
Abstract	2	Provide a summary of objectives, study design, setting, participants, sample size, predictors, outcome, statistical analysis, results, and conclusions.	✓
**Introduction**			
Background and objectives	3a	Explain the medical context (including whether diagnostic or prognostic) and rationale for developing or validating the multivariable prediction model, including references to existing models.	✓
	3b	Specify the objectives, including whether the study describes the development or validation of the model or both.	✓
**Methods**			
Source of data	4a	Describe the study design or source of data (e.g., randomized trial, cohort, or registry data), separately for the development and validation data sets, if applicable.	✓
	4b	Specify the key study dates, including start of accrual; end of accrual; and, if applicable, end of follow-up.	✓
Participants	5a	Specify key elements of the study setting (e.g., primary care, secondary care, general population) including number and location of centres.	✓
	5b	Describe eligibility criteria for participants.	✓
	5c	Give details of treatments received, if relevant.	✓
Outcome	6a	Clearly define the outcome that is predicted by the prediction model, including how and when assessed.	✓
	6b	Report any actions to blind assessment of the outcome to be predicted.	*
Predictors	7a	Clearly define all predictors used in developing or validating the multivariable prediction model, including how and when they were measured.	✓
	7b	Report any actions to blind assessment of predictors for the outcome and other predictors.	**
Sample size	8	Explain how the study size was arrived at.	✓
Missing data	9	Describe how missing data were handled (e.g., complete-case analysis, single imputation, multiple imputation) with details of any imputation method.	✓
Statistical analysis methods	10a	Describe how predictors were handled in the analyses.	✓
	10b	Specify type of model, all model-building procedures (including any predictor selection), and method for internal validation.	✓
	10d	Specify all measures used to assess model performance and, if relevant, to compare multiple models.	✓
Risk groups	11	Provide details on how risk groups were created, if done.	NA
**Results**			
Participants	13a	Describe the flow of participants through the study, including the number of participants with and without the outcome and, if applicable, a summary of the follow-up time. A diagram may be helpful.	***

	13b	Describe the characteristics of the participants (basic demographics, clinical features, available predictors), including the number of participants with missing data for predictors and outcome.	✓
Model development	14a	Specify the number of participants and outcome events in each analysis.	✓
	14b	If done, report the unadjusted association between each candidate predictor and outcome.	NA
Model specification	15a	Present the full prediction model to allow predictions for individuals (i.e., all regression coefficients, and model intercept or baseline survival at a given time point).	✓
	15b	Explain how to the use the prediction model.	****
Model performance	16	Report performance measures (with CIs) for the prediction model.	✓
**Discussion**			
Limitations	18	Discuss any limitations of the study (such as nonrepresentative sample, few events per predictor, missing data).	✓
Interpretation	19b	Give an overall interpretation of the results, considering objectives, limitations, and results from similar studies, and other relevant evidence.	✓
Implications	20	Discuss the potential clinical use of the model and implications for future research.	✓
**Other information**			
Supplementary information	21	Provide information about the availability of supplementary resources, such as study protocol, Web calculator, and data sets.	✓
Funding	22	Give the source of funding and the role of the funders for the present study.	✓

*** Report any actions to blind assessment of the outcome to be predicted**

Falls as an outcome requires no interpretation and blinding is therefore not relevant.

**** Report any actions to blind assessment of predictors for the outcome and other predictors**

Predictor values were almost entirely derived from objective measures and blinding is therefore not relevant.

***** Describe the flow of participants through the study, including the number of participants with and without the outcome and, if applicable, a summary of the follow-up time. A diagram may be helpful.**

The median follow-up period was 52 weeks (IQR 52-52).

****** Explain how to the use the prediction model**

A risk score can be calculated for any fall and recurrent falls by multiplying the value for each predictor with its respective regression coefficient and by then summing all values. The sum of these values (LP) can be converted into a risk probability using the following formula: p = 100% * (1 / (1 + exp(-LP))).

AD*F*ICE_IT harmonized cohort data

Data harmonization guide

Version October 2021

Introduction

Data were harmonized from six cohort studies, including The Irish Longitudinal Study on Ageing (TILDA) (public wave 1), the Longitudinal Aging Study Amsterdam (LASA) (waves C and 3B), the ActiFE Ulm Study (baseline wave), the B-PROOF study (baseline wave) and Rotterdam Study (ERGO-5). This data harmonisation guide describes how the variables were computed.
The variables were selected based on known risk factors of falls from previous studies focusing on demographic, health, lifestyle and environmental factors. Only those variables that were available in at least 3 of the 6 cohorts were harmonised. This guide describes for each of the variables [i] which relevant original items were available in each of the cohorts, [ii] the algorithm used to harmonise each of the variables, and [iii] the variable names, range and labels of the harmonised variables. The core principle of harmonisation is to capture the largest (most informative) common denominator.

A list of the variables ordered by theme and with hyperlinks is provided on page 5.

The cohorts

The **Longitudinal Ageing Study Amsterdam** (LASA) is an ongoing interdisciplinary cohort study on predictors and consequences of changes in physical, cognitive, emotional, and social functioning in men and women aged 55-85 years at baseline in 1992-93 (wave B). A random sample stratified for age, sex, and expected 5-year mortality was drawn from the population registries of 11 municipalities in the Netherlands.[3,4] New cohorts were initiated in 2002/2003 (wave 2B) and in 2012-13 (wave 3B) in persons aged 55-65 years. Measurement waves took place approximately every three years in all the three cohorts. For the current study, data from 1995/1996 (wave C, first cohort, n = 2545) and 2012-2013 (wave 3B, third cohort, n = 1023) were used. For both cohorts, it was asked whether the participant had fallen in the year before baseline. In addition, falls were recorded weekly on a fall calendar during three years starting at wave C.

The study on **Activity and Function in the Elderly in Ulm** (ActiFE Ulm) is embedded in a European funded study on the prevalence of COPD and asthma (Indicators for Monitoring COPD and Asthma - IMCA). A random sample of 7460 persons aged 65 years and over was selected from the population registers in Ulm, Neu-Ulm and Alb-Donau-Kreis. The recruitment phase started in February 2009 and finished in April 2010. In total, 1506 persons agreed to participate in the study. The primary focus is physical activity (as measured by sensor technology) and the consequences of physical activity for cognitive, emotional and social functioning.

The **Irish Longitudinal Study on Ageing** (TILDA) is an ongoing cohort study designed to achieve a representative sample of community-dwelling people aged 50 years or older in Ireland.[8] A random sample of 25600 residential addresses in Ireland were selected with stratification for socioeconomic status, age and geography. Each address was provided with study information and visited by field staff. All persons aged 50 years and over (primary respondents) and their spouses or partners of any age (secondary respondents) were eligible. Enrolled participants completed a computer-assisted questionnaire, self-completion questionnaire and a health assessment. Baseline data from the 8504 primary and secondary participants were collected between October 2009 and July 2011.

The **B-PROOF** study (B vitamins for PRevention Of Osteoporotic Fractures) is a 2-year randomized double-blind placebo-controlled trial, including 2,919 people aged 65 years or older, independently living or institutionalized, with an elevated homocysteine concentration (≥ 12 μmol/L). As the intervention was not effective, B-PROOF can be used as a cohort. Participants were recruited via registries of municipalities and elderly homes in the area of the research centres (Rotterdam, Amsterdam, Wageningen). One group received a daily tablet with 500 μg vitamin B12 and 400 μg folic acid and the other group received a placebo tablet. Both tablets included 15 μg (600 IU) vitamin D. The primary outcome of B-PROOF is time to first osteoporotic fracture. Falls were recorded weekly, on the research calendar. Baseline measurements took place between October 2008 and March 2011.

The **Rotterdam Study** is a prospective cohort study in the Ommoord district in the city of Rotterdam, the Netherlands, that started in 1990. The main objectives of the Rotterdam Study were to investigate the risk factors of cardiovascular, neurological, ophthalmological and endocrine diseases in the elderly. Up till now, the Rotterdam Study consists of three cohorts, for which the overall response figure at baseline was 72.0 %. Examinations were repeated every 3-4 years in potentially changing characteristics. Since the baseline cohort (RS-I-1), which included 7983 participants 55 years of age or over, four follow-up measurements were conducted. The most recent re-examination of the original cohort members was RS-I-6. Between 2009 and 2013, RS-I-5 (n=2147) was conducted as one project (i.e. 'ERGO-5') together with the third examination of the second cohort (RS-II-3, n=1893) and the second examination of the third cohort (RS-III-2, n=3122).

General instructions per cohort

LASA – The relevant data collection waves are wave C (1995-6) and 3B (i.e. the baseline assessment for the 3rd cohort completed in 2012-13). The data collection involves a face-to-face main interview, a face-to-face medical interview and a self-completed questionnaire. LASA stores its variables in separate files for each theme and each data collection wave. Details on data collection, cleaning and coding can be found for each of the variables on the LASA website (http://lasa-vu.nl/themes/navigator.htm). File names are structured as LASAC161 or LAS3B161, with 'C' referring to cohort 1 wave C and '3B' referring to cohort 3 wave B, respectively, and the number referring to the theme (e.g. '161' is anthropometry). Wave C includes prospective data on falls (i.e. fall calendars) for a period of 3 years as well as retrospective data for the past 12 months. For wave 3B data on retrospective falls for the past 12 months were available.

ActiFE Ulm – The relevant data collection wave is de baseline assessment (2009/2010). This was obtained by sending a data-analysis proposal to the ActiFE Ulm team and filling in the data request forms/contracts. ActiFE Ulm has been harmonised before in de EPOSA study, the data harmonisation guide of EPOSA (https://static-content.springer.com/esm/art%3A10.1186%2F1471-2474-12-272/MediaObjects/12891_2011_1300_MOESM1_ESM.PDF). The baseline wave includes retrospective fall data, but also a one-year follow-up calendar is available for both the medication and fall data.

TILDA – Survey 1 (2009-2011) is the relevant survey for the ADFICE_IT harmonisation. Survey 1 involves a self-completed questionnaire (SCQ), a computer-assisted personal interview (CAPI), and a health assessment (HAC). As the health assessment was done in the clinic, participants who were older, frailer and/or lived more remote were less likely to complete the health assessment. Sample weights are available to account for the selective drop-out. For survey 1, all variables (except medication use) are stored in one file per wave. For later surveys, variables of the CAPI and SCQ are stored in one file and the HAC data are stored in separate files for clinic (HAC) and home (HOME) assessments. Variables codes can be found in the questionnaire (available upon request) and the derived variables codebook (file:///R:/Wave%201/Derived%20Variables%20Codebook.html). Attrition data are stored in a separate file called "AuditTracker". Survey 1 includes retrospective fall data (i.e. fall in the past 12 months).

B-PROOF – The relevant data that will be used is the baseline data, conducted between October 2008 and March 2011. Baseline measurements were performed during a 1.5-2 hour session at one of the research centres or at the participants home. The B-PROOF codebook has been obtained, including information on variables, original questions and data availability (baseline only, follow-up only, baseline and follow-up). Information on medication data is not included in the codebook, but these data can be obtained from a separate access dataset. Data was obtained via B-PROOF contact persons. In B-PROOF falls are measured both prospectively (i.e. fall calendar) and retrospectively (i.e. fall past 12 months).

Rotterdam Study – The relevant cycle is the ERGO-5 cycle, as this is the newest wave with the medication data available. The RS-I-5, RS-II-3 and RS-III-2 examinations share the same project items; participants were interviewed at home and went through an extensive set of examinations in a specially built research facility in the centre of the district, such as bone mineral densitometry, including sample collections for in-depth molecular and genetic analyses. The codebook can be derived by obtaining access to a secluded website. Medication data is accessible via Bruno Stricker. The ERGO includes, both prospective (this concerns serious falls, i.e. falls leading to a hospital admission or leading to a fracture) as well as the retrospective (past 12-month) fall data.

Reference list

1. Huisman M, Poppelaars J, van der Horst M, et al. Cohort profile: the longitudinal aging study amsterdam. *Int J Epidemiol.* 2011;40(4):868-876.
2. Hoogendijk EO, Deeg DJ, Poppelaars J, et al. The Longitudinal Aging Study Amsterdam: cohort update 2016 and major findings. *Eur J Epidemiol.* 2016;31(9):927-945.
3. Kearney PM, Cronin H, O'Regan C, et al. Cohort profile: the Irish Longitudinal Study on Ageing. *Int J Epidemiol.* 2011;40(4):877-884.
4. Kriegsman DM, Penninx BW, van Eijk JT, Boeke AJ, Deeg DJ. Self-reports and general practitioner information on the presence of chronic diseases in community dwelling elderly. A study on the accuracy of patients' self-reports and on determinants of inaccuracy. *J Clin Epidemiol.* 1996;49(12):1407-1417.
5. Peeters GM, Alshurafa M, Schaap L, de Vet HC. Diagnostic accuracy of self-reported arthritis in the general adult population is acceptable. *J Clin Epidemiol.* 2015;68(4):452-459.
6. Van Wijngaarden JP, Dhonuskhe-Rutten RA, van Schoor NM, van der Velde N, Swart KM, Enneman AW, van Dijk SC, Brouwer-Brolsma EM, Zilikens MC, van Meurs JB, Brug J, Uitterlinden AG, Lips P, de Groot LC. Rationale and design of the B-PROOF study, a randomized controlled trial on the effect of supplemental intake of vitamin B12 and folic acid on fracture incidence. *BMC Geriatr.* 2011;11:80.
7. Schaap LA, Peeters GMEE, Dennison EM, Zambon S, Nikolaus T, Sanchez-Martinez M, Musacchio E, van Schoor NM, Deeg DJH, and the EPOSA research group. European Project on Osteoarthritis (EPOSA): methodological challenges in hamonization of existing data from five European population-based cohorts on aging. *BMC Musculoskelet Disord.* 2011;12:272.
8. Ikram MA, Brusselle GGO, Murad SD, van Duijn CM, Franco OH, Goedegebure A, Klaver CCW, Nijsten TEC, Peeters RP, Stricker BH, Tiemeier H, Uitterlinden AG, Vernooij MW, Hofman A. The Rotterdam Study: 2018 update on objectives, design and main results.
9. Denkinger, MD, Franke S, Rapp, Kilian R, Weinmayr G, Duran-Tauleria E, Nikolaus T, Peter R, ActiFE Ulm Study Group. Accelerometer-based physical activity in a large observational cohort – study protocol and design of the activity and function of the elderly in Ulm (ActiFE Ulm) study. *BMC Geriatrics.* 2010;10(50).

List of variables by theme

Generic variables	Chronic conditions	Cognitive functioning	General health and symptoms	Blood markers
Cohort	Anxiety	Executive function	Blood pressure	CRP
ID number	Arthritis	Memory	Body mass index (BMI)	Vitamin D
Year of data collection	Cancer	MMSE	Dizziness	Vitamin B12
	Cardiovascular disease	Reaction time	Functional limitations	Creatinine
**Socio-demographic**	Arrhythmia	Speed of processing	Hearing	
Age	Angina pectoris		Height	**Medication**
Education	Myocardial infarction	**Physical Performance / physical functioning**	Pain	Number of medications
Income	Heart failure	Walking aid	Pulse rate	Medication use
Marital status	Stroke	Balance	Self-rated health	Anticholinergic medication
Number of people in household	Depression	Grip strength	Sleep	Medication for hypertension
Occupation and retirement	Diabetes	Gait speed	Urinary incontinence	
Urbanisation	Heart disease		Visual problems	
Sex	Hypertension	**Lifestyle**	Waist circumference	
	Lung disease	Alcohol use	Weight	
**Falls**	Number of chronic conditions	Smoking	Weight loss	
Faller	Parkinson’s disease	Physical activity	Genetic variants	
Number of falls			Quality of life	
Fear of falls				
Injurious falls				
Falls in Follow-up (time to first fall)				

<table>
<tr><th>Cohort</th><th>Original items (variable name)</th><th>Range or category labels</th><th>Harmonisation algorithm</th><th>Harmonised variable details</th></tr>
<tr><td colspan="5">Age</td></tr>
<tr><td>LASA</td><td>Age at LASA main/telephone interview (bage, cage)</td><td>bage: 54.95-65.55 yrs
cage: 57.94-88.85 yrs</td><td>Round to lower integer (e.g. 57.7 → 57) to remove decimals.
If age >= 80 → age = 80</td><td rowspan="5">Variable name:
age
agemax80

Variable label:
Age in years
Age in years max value is 80

For TILDA, respondents below the age of 50 or whose age was missing were excluded (n=341).</td></tr>
<tr><td>ActiFE Ulm</td><td>age at visit 1 (age_BL_v1)</td><td>Range 65.3-91.4</td><td>Round to lower integer (e.g. 57.7 → 57) to remove decimals.
If age >= 80 → age = 80</td></tr>
<tr><td>TILDA</td><td>Age at interview assuming DOB is 1st of specified month (age)

) Note that the sampling frame was set up to target residents aged 50+, but partners were also invited to participate, which resulted in the inclusion of 329 participants aged <50 at baseline.</td><td>Range 29-105 yrs
Ages <50 and 80+ are all '49' and '80', respectively. So: the actual age range in the public Tilda data is missing (maximum age=80, minimum=49)?</td><td>Use as is.</td></tr>
<tr><td>B-PROOF</td><td>Age of participant at baseline, based on date of birth and date baseline interview (Age).</td><td>Range 63 - 98 years</td><td>Use as is.
If age >= 80 → age = 80</td></tr>
<tr><td>Rotterdam Study</td><td>Date of birth and interview date (Date_of_birth, e5_3493)</td><td>Range interview date:
Date >= today
01-JAN-1582 = default/missing</td><td>Age = e5_3493 - Date_of_birth
If age >= 80 → age = 80</td></tr>
<tr><td colspan="5">Alcohol use</td></tr>
<tr><td>LASA</td><td>Do you drink alcohol? (cmvar24, BMALCOHU)

How many days per week do you drink alcohol (past year)? (cmvar25a, BMALCOHD)

How many consumptions do you drink each time? (cmvar28, BMALCOHN)

Number of times six glasses or more? (cmvar29, BMALCOH6)</td><td>1= no
2= yes

1= every day
2= 5-6 days/week
3= 3–4 days/week
4= 1-2 days/week
5= 1-3 days/month
6= < 1 day/month

1= 11+ drinks
2= 8-10 drinks
3= 6-7 drinks
4= 4-5 drinks
5= 2-3 drinks
6= 1 drink

1= Every day
2= 5-6 days/week
3= 3-4 days/week
4= 1-2 days/week
5= 1-3 days/month
6= <1 day/month</td><td>alcohol
<table>
<tr><th></th><th colspan="7">quantity of alcohol consumed</th></tr>
<tr><th></th><th>n/a</th><th>1</th><th>2-3</th><th>4-5</th><th>6-7</th><th>8-10</th><th>11+</th></tr>
<tr><td>no alcohol</td><td>0</td><td>1</td><td>1</td><td>1</td><td>1</td><td>1</td><td>1</td></tr>
<tr><td><1 day/month</td><td></td><td>1</td><td>1</td><td>1</td><td>1</td><td>1</td><td>1</td></tr>
<tr><td>1-3 days/month</td><td></td><td>2</td><td>2</td><td>2</td><td>2</td><td>2</td><td>2</td></tr>
<tr><td>1-2 days</td><td></td><td>2</td><td>2</td><td>2</td><td>2</td><td>2</td><td>3</td></tr>
<tr><td>3-4 days</td><td></td><td>2</td><td>2</td><td>3</td><td>3</td><td>4</td><td>4</td></tr>
<tr><td>5-6 days</td><td></td><td>2</td><td>3</td><td>4</td><td>4</td><td>4</td><td>4</td></tr>
<tr><td>every day</td><td></td><td>2</td><td>3</td><td>4</td><td>4</td><td>4</td><td>4</td></tr>
</table>

alcoholyn, wave C:
if cmvar24=1 → alcoholyn= 0
if cmvar24=2 → alcoholyn= 1

alcoholyn, wave 3B:
if BMALCOHU=1 → alcoholyn=0
if BMALCOHU=2 → alcoholyn=1

alcoholfr, wave C:
if cmvar24 = 1 → alcoholfr = 0
if cmvar25a = 6 → alcoholfr = 1
if cmvar25a = 5 → alcoholfr = 2
if cmvar25a = 3 or cmvar25a = 4 → alcoholfr = 3
if cmvar25a = 1 or cmvar25a = 2→ alcoholfr = 4

alcoholfr, wave 3B:
if BMALCOHU = 1 → alcoholfr = 0
if BMALCOHD = 6 → alcoholfr = 1
if BMALCOHD = 5 → alcoholfr = 2
if BMALCOHD = 3 or BMALCOHD = 4 → alcoholfr = 3
if BMALCOHD = 1 or BMALCOHD = 2→ alcoholfr = 4</td><td>Variable name:
1. alcohol
2. alcoholyn
3. alcoholfr

Variable label:
1. Alcohol intake
2. Do you drink alcohol?
3. Frequency of alcohol use

Value labels:
alcohol
0=non-drinker
1=rarely drinks
2=low risk (≤14 per week)
3=risky (15-28 per week)
4=high risk (>28 per week)

alcoholyn
0=no
1=yes

alcoholfr
0=non-drinker
1=less than once a month
2=1-3 times a month
3=1-4 days a week
4=(almost) daily</td></tr>
</table>

<table>
<tr>
<td>ActiFE Ulm</td>
<td>Frequency of alcohol use (alcohol_freq_BL)

Average quantity was not measured.</td>
<td>1 = daily
2 = several times a week
3 = several times a month
4 = less than once in a month
5 = never</td>
<td>alcohol
Not harmonized; average quantity was not measured.

alcoholyn
if alcohol_freq_BL=5 → alcoholyn=0
if alcohol_freq_BL>=1 and alcohol_freq_BL<=4 → alcoholyn=1

alcoholfr
if alcohol_freq_BL =5 → alcoholfr=0
if alcohol_freq_BL =4 → alcoholfr=1
if alcohol_freq_BL =3 → alcoholfr=2
if alcohol_freq_BL =2 → alcoholfr=3
if alcohol_freq_BL =1 → alcoholfr=4</td>
<td rowspan="2">Notes: (1) More info on the alcohol tables is provided in appendix 3. (2) For alcoholfr, there are slight differences in the cut-off values for the harmonization algorithms of the different algorithms. For example, one a month or less in the Rotterdam study was harmonized as less than once a month.</td>
</tr>
<tr>
<td>TILDA</td>
<td>Do you drink alcohol? (SCQAlcohol)

In the last <u>6 months</u>, how often have you had drinks containing alcohol? (SCQAlcoFreq)*

How many drinks consumed on days drink taken? (SCQAlcoNo2)

*) Note that some participants ticked two adjacent boxes, in which case the higher frequency was used (e.g. if 3-4 days/week and 5-6 days/week were ticked, 5-6 days/week was used).</td>
<td>1= yes
2= no

1= almost every day*
2= 5-6 days/week*
3= 3-4 days/week*
4= 1-2 days/week*
5= 1-2 days/month*
6= < 1 day/month*
7= not at all in the last 6 months*

Range 0-10
(10=10+ glasses)</td>
<td>alcohol
Frequency of drinking was based on SCQAlcoFreq and quantity of drinking was based on SCQAlcoNo2.
<table>
<tr><td></td><td colspan="7">quantity of alcohol consumed</td></tr>
<tr><td></td><td>.</td><td>0</td><td>1-2</td><td>3-4</td><td>5-6</td><td>7-8</td><td>9+</td></tr>
<tr><td>not at all</td><td>0</td><td>0</td><td>1</td><td>1</td><td>1</td><td>1</td><td>1</td></tr>
<tr><td><1 day/month</td><td></td><td></td><td>1</td><td>1</td><td>1</td><td>1</td><td>1</td></tr>
<tr><td>1-2 days/month</td><td></td><td></td><td>2</td><td>2</td><td>2</td><td>2</td><td>2</td></tr>
<tr><td>1-2 day/week</td><td></td><td></td><td>2</td><td>2</td><td>2</td><td>3</td><td>3</td></tr>
<tr><td>3-4 days/week</td><td></td><td></td><td>2</td><td>3</td><td>3</td><td>3</td><td>4</td></tr>
<tr><td>5-6 days/week</td><td></td><td></td><td>2</td><td>3</td><td>4</td><td>4</td><td>4</td></tr>
<tr><td>almost every day</td><td></td><td></td><td>2</td><td>3</td><td>4</td><td>4</td><td>4</td></tr>
</table>

alcoholyn
if SCQAlcohol=2 → alcoholyn=0
if SCQAlcohol=1 → alcoholyn=1

alcoholfr
if SCQAlcoFreq =7 or SCQAlcohol = 2 → alcoholfr=0
if SCQAlcoFreq =6 → alcoholfr=1
if SCQAlcoFreq =5 → alcoholfr=2
if SCQAlcoFreq =4 or SCQAlcoFreq =3→ alcoholfr=3
if SCQAlcoFreq =2 or SCQAlcoFreq =1 → alcoholfr=4</td>
</tr>
</table>

<table>
<tr>
<td>B-PROOF</td>
<td>Do you sometimes drink alcohol? (alcohol)

Did you drink alcohol last year? (alcohol_last_year)

When you drink alcohol, how many glasses do you have? (alcohol_amount)

How often did you drink more than 6 glasses in one day? (alcohol_large_amount)</td>
<td>0 = no
1 = yes

1 = daily
2 = 5-6 days per week
3 = 3-4 days per week
4 = 1-2 days per week
5 = 1-3 days per month
6 = < 1 day per month

1 = 11+ glasses
2 = 8-10 glasses
3 = 6-7 glasses
4 = 4-5 glasses
5 = 2-3 glasses
6 = 1 glass

1 = everyday
2 = 5-6 days per week
3 = 3-4 days per week
4 = 1-2 days per week
5 = 1-3 days per month
6 = < 1 day per month</td>
<td>alcohol
<table>
<tr><td></td><td colspan="7">quantity of alcohol consumed</td></tr>
<tr><td></td><td>n/a</td><td>1</td><td>2-3</td><td>4-5</td><td>6-7</td><td>8-10</td><td>11+</td></tr>
<tr><td>no alcohol</td><td>0</td><td>1</td><td>1</td><td>1</td><td>1</td><td>1</td><td>1</td></tr>
<tr><td><1 day/month</td><td></td><td>1</td><td>1</td><td>1</td><td>1</td><td>1</td><td>1</td></tr>
<tr><td>1-3 days/month</td><td></td><td>2</td><td>2</td><td>2</td><td>2</td><td>2</td><td>2</td></tr>
<tr><td>1-2 days</td><td></td><td>2</td><td>2</td><td>2</td><td>2</td><td>2</td><td>3</td></tr>
<tr><td>3-4 days</td><td></td><td>2</td><td>2</td><td>3</td><td>3</td><td>4</td><td>4</td></tr>
<tr><td>5-6 days</td><td></td><td>2</td><td>3</td><td>4</td><td>4</td><td>4</td><td>4</td></tr>
<tr><td>every day</td><td></td><td>2</td><td>3</td><td>4</td><td>4</td><td>4</td><td>4</td></tr>
</table>

alcoholyn*
if alcohol=0 → alcoholyn=0
if alcohol=1 → alcoholyn=1

alcoholfr*
if alcohol=0 → alcoholfr=0
if alcohol_last_year =6 → alcoholfr=1
if alcohol_last_year =5 → alcoholfr=2
if alcohol_last_year =4 or alcohol_last_year =3 → alcoholfr=3
if alcohol_last_year =2 or alcohol_last_year =1 → alcoholfr=4

*) alcoholyn and alcoholfr were computed prior to the harmonized alcohol variable.</td>
<td></td>
</tr>
<tr>
<td>Rotterdam Study</td>
<td>How often did you use alcohol in the past year? (e5_EIAUDIT1)

How many glasses did you drink on one day? (e5_EIAUDIT2)

How often do you drink six glasses or more on one occasion? (e5_EIAUDIT3)</td>
<td>0=never
1=one a month or less
2=2-4 times a month
3=2-3 times a week
4=4 of more times a week
7=don't know
9=no answer

0=1 or 2
1=3 or 4
2=5 or 6
3=7 to 9
4=10 or more
7=don't know
9=no answer

0=never
1=less than monthly
2=monthly (1-3 times a month)
3=weekly (1-3 times a week)
4= (almost) daily (4 or more times a week)
7=don't know
9=no answer</td>
<td>
<table>
<tr><td></td><td colspan="6">Quantity of alcohol consumed</td></tr>
<tr><td></td><td>.</td><td>1-2</td><td>3-4</td><td>5-6</td><td>7-9</td><td>10+</td></tr>
<tr><td>never</td><td>0</td><td>1</td><td>1</td><td>1</td><td>1</td><td>1</td></tr>
<tr><td><1 day/month</td><td></td><td>1</td><td>1</td><td>1</td><td>1</td><td>1</td></tr>
<tr><td>2-4 times/month</td><td></td><td>2</td><td>2</td><td>2</td><td>2</td><td>3</td></tr>
<tr><td>2-3 times/week</td><td></td><td>2</td><td>2</td><td>3</td><td>3</td><td>4</td></tr>
<tr><td>4+ times/week</td><td></td><td>2</td><td>3</td><td>4</td><td>4</td><td>4</td></tr>
</table>
alcohol
*non-drinker.
if e5_EIAUDIT1=0 → alcohol=0

*rarely drinks.
if e5_EIAUDIT1=0 and e5_EIAUDIT2>=0 and e5_EIAUDIT2<=4 → alcohol=1
if e5_EIAUDIT1=1 and e5_EIAUDIT2>=0 and e5_EIAUDIT2<=4 → alcohol=1

*low risk.
if e5_EIAUDIT1=2 and e5_EIAUDIT2>=0 and e5_EIAUDIT2<=3 → alcohol=2
if e5_EIAUDIT1=3 and e5_EIAUDIT2>=0 and e5_EIAUDIT2<=1 → alcohol=2
if e5_EIAUDIT1=4 and e5_EIAUDIT2=0 → alcohol=2

*risky.
if e5_EIAUDIT1=2 and e5_EIAUDIT2=4 → alcohol=3
if e5_EIAUDIT1=3 and (e5_EIAUDIT2=2 or e5_EIAUDIT2=3) → alcohol=3
if e5_EIAUDIT1=4 and e5_EIAUDIT2=1 alcohol=3

*high risk.
if e5_EIAUDIT1=3 and e5_EIAUDIT2=4 alcohol=4
if e5_EIAUDIT1=4 and e5_EIAUDIT2>=2 and e5_EIAUDIT2<=4 → alcohol=4

alcoholyn
if e5_EIAUDIT1=0 → alcoholyn=0
if e5_EIAUDIT1>=1 and e5_EIAUDIT1<=4 → alcoholyn=1

alcoholfr
if e5_EIAUDIT1 =0 → alcoholfr=0
if e5_EIAUDIT1 =1 → alcoholfr=1</td>
<td></td>
</tr>
</table>

			if e5_EIAUDIT1 =2 → alcoholfr=2<br>if e5_EIAUDIT1 =3 → alcoholfr=3<br>if e5_EIAUDIT1 =4 → alcoholfr=4	
Anxiety				
LASA	HADS-A Anxiety scale<br>Summary score: (banxint, canxint)<br>-feel tense or wound up (banxiet1, canxiet1)<br>-frightened feeling (banxiet2, canxiet2)<br>-worrying thoughts (banxiet3, canxiet3)<br>-I feel relaxed: positive (banxiet4, canxiet4)<br>-frightened in stomach (banxiet5, canxiet5)<br>-feel restless (banxiet6, canxiet6)<br>-sudden feelings of panic (banxiet7, canxiet7)	Range 0-21<br>0=rarely or never<br>1=some of the Time<br>2=occasionally<br>3=mostly or always	HADSA=banxint	Variable name:<br>HADSA<br><br>Variable label:<br>HADS-Anxiety score (range 0-21)<br><br>Value label:<br>0 = low (minimum)<br>8= cut-off<br>21= high (maximum)<br><br>All cohorts include the HADS Anxiety scale, except for B-PROOF.
ActiFE Ulm	HADS-A score (anxiety subscale) (HADS_A_score_BL)	Summary score: 0-21	HADSA=HADS_A_score_BL	
TILDA	HADS-A Anxiety scale (MHhadsa):<br>- feel tense or wound up (SCQAnxiety1)*<br>- frightened feeling as if something awful is about to happen (SCQAnxiety2)*<br>- worrying thoughts (SCQAnxiety3)*<br>- sit at ease and feel relaxed (SCQAnxiety4)*<br>- I get a sort of frightened feeling like "butterflies" in the stomach (SCQAnxiety5)*<br>- I feel restless as if I have to be on the move (SCQAnxiety6)*<br>- I get sudden feelings of panic (SCQAnxiety7)*	Summary score: 0-21<br>1=very often indeed*<br>2=quite a lot*<br>3=not very often*<br>4=not at all*	HADSA=MHhadsa	

B-PROOF	No anxiety scale present.<br>Item from Euroquol about mood (depression/anxiety) (Euroqol_5)<br><br>Items from SF12:<br>- During the past four weeks, have you accomplished less than you would like to as a result of any emotional problems, such as feeling depressed or anxious? (SF12_6)<br>- During the past four weeks, did you not do work or other regular activities as carefully as usual as a result of any emotional problems such as feeling depressed or anxious? (SF12_7)	Range 1-3:<br>1 = not depressed/scared<br>2 = a bit depressed/scared<br>3 = severely depressed/scared<br><br>0 = no<br>1 = yes	Not harmonized.	
Rotterdam Study	Anxiety disorders were assessed using a slightly adapted version of the Munich Composite International Diagnostic Interview, to obtain DSM-IV diagnoses of generalized anxiety disorder, panic disorder, agoraphobia, social phobia and specific phobia. Obsessive compulsive disorder and post-traumatic stress disorder were not included<br><br>Furthermore; the HADS-A Anxiety scale was used:<br>- feel tense or wound up (e5_EIHADS01)*<br>- frightened feeling as if something awful is about to happen (e5_EIHADS03)*<br>- worrying thoughts (e5_EIHADS05)*<br>- sit at ease and feel relaxed (e5_EIHADS07)*<br>- I get a sort of frightened feeling like "butterflies" in the stomach (e5_EIHADS09)*<br>- I feel restless as if I have to be on the move (e5_EIHADS11)*<br>- I get sudden feelings of panic (e5_EIHADS13)*	0 = no<br>1 = yes<br>7 = don't know<br>9 = no answer<br><br>Range 0-21<br>0=most of the time<br>1=often<br>2=sometimes<br>3=never<br>7=don't know<br>9=no answer<br><br>*Order of categories (0-3 or 3-0) differs per question; item 7 and 9 are reverse of anxious. Item 7:<br>0=certainly<br>1=most of the time<br>2=not often<br>3=not at all<br>7=don't know<br>9=no answer<br><br>Item 9:<br>0=not at all<br>1=sometimes<br>2=quite often<br>3=very often<br>7=don't know<br>9=no answer	For item 1, 3, 5, 11, 13: categories are recoded, e.g.:<br>if (e5_EIHADS01=0) hads1=3.<br>if (e5_EIHADS01=1) hads1=2.<br>if (e5_EIHADS01=2) hads1=1.<br>if (e5_EIHADS01=3) hads1=0.<br><br>Finally, the original item 7 and 9 and the recoded items 1, 3, 5, 11, 13 are all summed up and computed as HADSA.<br><br>For n=78: at least one of the single items was missing.	

Arthritis				
LASA	Do you have joint damage or osteoarthritis of the knees, hips or hands? (BRHEUM01, CRHEUM01)<br>Do you have joint inflammation meaning chronic rheumatism or rheumatoid arthritis? (BRHEUM02, CRHEUM02)	<u>3B:</u><br>1=no<br>2=yes<br><br><u>C:</u><br>0=no, never<br>1=no, but at wave B yes<br>2=yes, but at wave B no<br>3=yes, and at wave B yes	**Arthritis, wave 3B:**<br>If BRHEUM01=2 or BRHEUM02=2 → arthritis =1<br>If BRHEUM01=1 and BRHEUM02=1 → arthritis =0*<br><br>**Arthritis, wave C:**<br>if crheum01=1 or crheum01=2 or crheum01=3 or crheum02=1 or crheum02=2 or crheum02=3 → arthritis=1<br>if crheum01=0 and crheum02=0 → arthritis=0*<br><br>**Osteoarthritis, wave 3B:**<br>If BRHEUM01=2 → osteoarthritis=1<br>If BRHEUM01=1 → osteoarthritis=0<br><br>**Osteoarthritis, wave C:**<br>if crheum01=1 or crheum01=2 or crheum01=3 → osteoarthritis=1<br>if crheum01=0 → osteoarthritis=0<br><br>**Rheumatoid, wave 3B:**<br>If BRHEUM02=2 → rheumatoid =1<br>If BRHEUM02=1 → rheumatoid =0<br><br>**Rheumatoid, wave C:**<br>if crheum02=1 or crheum02=2 or crheum02=3 → rheumatoid=1<br>if crheum02=0 → rheumatoid=0<br><br>*) There were no cases in which only one of the two variables were missing.	Variable name:<br>1. arthritis<br>2. osteoarthritis<br>3. rheumatoid<br><br>Variable label:<br>1. Arthritis: OA, RA or other<br>2. Osteoarthritis<br>3. Rheumatoid arthritis<br><br>Value labels:<br>0=no<br>1=yes<br><br>Notes:<br>The distribution of RA in the Rotterdam Study differs greatly from that of the other cohorts.
ActiFE Ulm	arthritis until baseline (C_arthritis_BL)<br><br>rheumatism until baseline (C_rheuma_BL)<br><br>Rheumatism was determined using the following question: *has a doctor ever told you that you have Rheumatic diseases? Such as classic rheumatism or lupus (not osteoarthritis / arthritis).*<br><br>No data for osteoarthritis.	0=no<br>1=yes	**arthritis**<br>If C_arthritis_BL =1 → arthritis=1 or<br>If C_rheuma_BL =1 → arthritis=1<br><br>If C_arthritis_BL =0 and<br>If C_rheuma_BL =0 → arthritis=0<br><br>**osteoarthritis**<br>Not harmonized.<br><br>**rheumatoid**<br>If C_rheuma_BL=1 → rheumatoid=1<br>If C_rheuma_BL =0 → rheumatoid=0	
TILDA	Has a doctor ever told you that you have any of the following conditions: arthritis (including osteoarthritis or rheumatism) (ph301_03)<br>If yes, which type or types of arthritis do you have:<br>-osteoarthritis (ph304_1)<br>-rheumatoid arthritis (ph304_2)<br>-some other kind of arthritis (ph304_3)<br>-don't know (ph304_4)	0=no<br>1=yes<br>There are no missing cases for ph301_03.<br><br>-1= n/a (in case ph301_03 =0)<br>0=no<br>1=yes	**arthritis**<br>If ph301_03=1 → arthritis=1<br>If ph301_03=0 → arthritis=0<br><br>**osteoarthritis**<br>If ph304_1=1 → osteoarthritis=1<br>If ph304_1<=0 → osteoarthritis=0<br><br>**rheumatoid**<br>If ph304_2=1 → rheumatoid=1<br>If ph304_2<=0 → rheumatoid=0	
B-PROOF	No data available.		Not harmonized.	

Rotterdam Study	Were you ever diagnosed with rheumatoid arthritis by a rheumatologist? (e5_EIRAA)<br><br>When was rheumatoid arthritis diagnosed for the first time? (e5_eiraayr)<br><br>Have you ever gotten any medication for reumatoid arthritis? (e5_EIRAATX)<br><br>Have you ever experienced any pain or other complaints in your knees, back, hips or hand joints? (e5_EI3_00)<br><br>Do you experience any pain or stiffness in your knees? (e5_EI3KN01B)<br><br>Have you been to your general practitioner for your joint complaints? (e5_EI3ZG1)<br><br>Have you been to a specialist for your joint complaints? If yes, what specialist? (e5_EI3ZG4)<br><br>Do you know what it is that you have? Did your general practitioner or the specialist tell you what it is? (e5_EI3ZG5)<br><br>No data for osteoarthritis.	0 = no<br>1 = yes<br>7 = don't know<br>9 = no answer<br><br>Date<br><br>0 = no<br>1 = yes<br>7 = don't know<br>8= n.a.<br>9 = no answer<br><br>0 = no<br>1 = yes<br>7 = don't know<br>9 = no answer<br><br>0 = no<br>1 = yes, pain<br>2 = yes, stiffness<br>3 = yes, pain and stiffness<br>7 = don't know<br>9 = no answer<br><br>0 = no<br>1 = yes<br>7 = don't know<br>8= n.a.<br>9 = no answer<br><br>0 = no<br>1 = yes<br>7 = don't know<br>8= n.a.<br>9 = no answer<br><br>0 = no<br>1 = arthrose (worn joint)<br>2 = reumatoid arthritis (chronic)<br>3 = gout<br>4 = spit or sciatica<br>5 = Bekhterev's disease<br>6 = other<br>7 = don't know<br>8 = n.a.<br>9 = no answer	**arthritis**<br>if e5_EI3ZG5=0 or e5_EI3ZG5=4 or e5_EI3ZG5=8 → arthritis=0<br>if e5_EI3ZG5=1 or e5_EI3ZG5=2 or e5_EI3ZG5=3 or e5_EI3ZG5=5 or e5_EI3ZG5=6→ arthritis=1<br><br>**osteoarthritis**<br>Not harmonized.<br><br>**rheumatoid**<br>if e5_EI3ZG5=0 or e5_EI3ZG5=1 or (e5_EI3ZG5>=3 and e5_EI3ZG5<=6) or e5_EIRAA =0 → rheumatoid=0<br>if e5_EI3ZG5=2 or e5_EIRAA =1 → rheumatoid=1	

Balance				
LASA	Tandem stance (up to 10 sec in C and up to 30 sec in 3B):<br>-Time in seconds (btandem2, ctandem2)<br>-Able to perform tandem stand test (btandem1, ctandem1)	Range 0-10/30 seconds<br>1=normal test<br>2=not capable<br>3=falls almost direct<br>4=stops within 3 sec<br>5=refusal<br>6=physically impossible	**balance, wave C:**<br>If (ctandem1>=2 and ctandem1<=4) or ctandem1=6 → balance=0<br>If ctandem2>=3 and ctandem2<=9 → balance=1<br>If ctandem2>=10 → balance=2<br><br>**balanceyn, wave C:**<br>If ctandem1>=2 and ctandem1<=4 or ctandem1=6 → balanceyn=0<br>If ctandem2>=3 and ctandem2<=9 → balanceyn=0<br>If ctandem2>=10 → balanceyn=1<br><br>**balance, wave 3B:**<br>If (btandem1>=2 and btandem1<=4) or btandem1=6 → balance=0<br>If btandem2>=3 and btandem2<=9 → balance=1<br>If btandem2>=10 → balance=2<br><br>**balanceyn, wave 3B:**<br>If btandem1>=2 and btandem1<=4 or btandem1=6 → balanceyn=0<br>If btandem2>=3 and btandem2<=9 → balanceyn=0<br>If btandem2>=10 → balanceyn=1	Variable name:<br>1.balance<br>2.balanceyn<br><br>Variable label:<br>1.Ability to perform tandem test<br>2. Ability to perform tandem test (yes/no)<br><br>Value label:<br>**balance:**<br>0=unable<br>1=3-9 sec: poor performance<br>2=10+ sec: good performance<br><br>**balanceyn:**<br>0=unable <10s<br>1=able >10s
ActiFE Ulm	As part of the SPPB, a semitandam stand, side-by-side stand, and a tandem stand were done. The combined results of these tests were reported using a five-point scale.<br><br>SPPB typically uses the following instructions for these tests: "Begin with a semitandem stand (heel of one foot placed by the big toe of the other foot). Individuals unable to hold this position should try the side-by-side position. Those able to stand in the semitandem position should be tested in the full tandem position. Once you have completed time measures, complete ordinal scoring."<br><br>Balance test (SPPB_b_cat_BL)	0 = side by side 0-9 sec or unable<br>1 = side by side 10, <10 sec semitandem<br>2 = semitandem 10 sec, tandem 0-2 sec<br>3 = semitandem 10 sec, tandem 3-9 sec<br>4 = tandem 10 sec	**balance**<br>if SPPB_b_cat_BL = 0 or SPPB_b_cat_BL = 1 or SPPB_b_cat_BL = 2 → balance = 0<br>if SPPB_b_cat_BL = 3 → balance = 1<br>if SPPB_b_cat_BL = 4 → balance = 2<br><br>**balanceyn**<br>if SPPB_b_cat_BL >= 0 and SPPB_b_cat_BL <= 3→ balanceyn = 0<br>if SPPB_b_cat_BL = 4 → balanceyn = 1	
TILDA	We are interested in your steadiness when walking, standing or getting up from a chair.<br>-When walking, do you feel: (PH411)<br>-When standing, do you feel: (PH412)<br>-When getting up from a chair, do you feel: (PH413)	1. very steady,<br>2. slightly steady,<br>3. slightly unsteady,<br>4. very unsteady<br>98. DK<br>99. RF	Not harmonized; no objective test performed (only TUG, which was used for mobility variable).	

B-PROOF	**Subjective measure:**<br>Subjective 2yr change in balance. (FU1_physicalfunc_balance)<br><br>**Objective measures:**<br>Tandem test:<br>- Tandem score (PP26)<br><br>*NB: PP26 is assigned by the interviewer, but might be incorrect. Tandem score was calculated again by PP_tandem, via PP_time (see sumscores)*<br><br>-Time in seconds (PP_time)<br>-Able to perform tandem stand test (PP_tandem)<br><br>Romberg: position (pp23)<br><br>Romberg: eyes open/closed (pp24)<br><br>Romberg: time in seconds (pp25)	1 = much better<br>2 = somewhat better<br>3 = no change<br>4 = Somewhat worse<br>5 = much worse<br><br>1 = normal test<br>2 = not capable<br>3 = falls almost direct<br>4 = stops within 3 sec<br>5 = refusal<br>6 = physical impossible<br><br>Time of tandem stand<br>Tandem score:<br>0 = unable<br>2 = able to hold 3-9 seconds<br>4 = able to hold at least 10 seconds<br><br>**PP23**<br>0 = not possible<br>1 = position feet: shoulder width<br>2 = position feet: closed<br>3 = position feet: stride stand<br>4 = position feet: tightrope walker<br><br>1 = eyes open<br>2 = eyes closed<br><br>Time of Romberg position	**Balance:**<br>If PP_tandem=0 → balance=0<br>If PP_tandem=2 → balance=1<br>If PP_tandem=4 → balance=2<br><br>**Balanceyn:**<br>If PP_tandem=0 or PP_tandem=2 → balanceyn=0<br>If PP_tandem=4 → balanceyn=1	

Rotterdam Study	Standing, eyes opened (e5_16385):<br>Standing position.<br>Can you stand on one foot? If not, can you stand in tandem position (one foot before the other).<br>If this is not possible, can you stand with your feet right next to eachother?<br>Natural position; can your stand in a comfortable position?<br>Standing, eyes opened.<br><br>PD – Postural reflex, standing, with researcher standing behind participant<br><br>PD – Body swing with feet together, eyes opened<br>PD - Body swing with feet together, eyes closed	0 = normal, standing on one foot >10s.<br>1 = feet together, not one foot >10s.<br>2 = feet together, not in tandem position.<br>3 = feet not together, natural position.<br>4 = without support, natural position, with correction.<br>5 = natural position with support of one arm<br>6 = cannot stand<br>8 = not applicable/default<br>9 = missing<br><br>1 = normal<br>2 = declined<br>3 = not judgeable<br>9 = missing<br>8 = not applicable/default<br><br>0 = normal<br>1 = a little unsteady<br>2 = some unsteadiness (head<10cm)<br>3 = very unsteady (head>10cm)<br>4 = falling directly<br>9 = missing<br>8 = not applicable/default	**Balance**:<br>Not harmonized; no data on duration of tandem test.<br><br>**Balanceyn**:<br>If e5_16385=0 or e5_16385=1 → balanceyn=1<br>If e5_16385>=2 and e5_16385<=6 → balanceyn=0	
Blood pressure				
LASA	Blood pressure measured in duplicate in 3B on the upper arm while sitting using an automatic Omron device (left arm, if not possible right). In LASA C, blood pressure was first measured once using the respondents finger. Then, blood pressure was measured once in sitting position, in lying position and in standing position.<br>Systolic, finger (cmvar9)<br>Diastolic, finger (cmvar10)<br>Systolic, sitting (bmarmss1, bmarmss2, cmvar800)<br>Diastolic, sitting (bmarmds1, bmarmds2, cmvar801)<br>Systolic, lying (cmvar803)<br>Diastolic, lying (cmvar804)<br>Systolic, standing (cmvar806)<br>Diastolic, standing (cmvar807)<br><br>Blood pressure measurements using a finger cuff are considered to be relatively inaccurate (https://www.ncbi.nlm.nih.gov/pubmed/17280997).	In mmHg<br>Missing wave C: n=36<br>Missing wave 3B:<br>1: n=26<br>2: n=35	**BPsys/BPdias, wave C:**<br>if (cmvar800>0 and cmvar803<=0 and cmvar806<=0) → BPsys=cmvar800<br>if (cmvar801>0 and cmvar804<=0 and cmvar807<=0) → BPdias=cmvar801<br>if (cmvar800>0 and cmvar803>0 and cmvar806<=0) → BPsys=min(cmvar800, cmvar803)<br>if (cmvar801>0 and cmvar804>0 and cmvar807<=0) → BPdias=min(cmvar801, cmvar804)<br>if (cmvar800>0 and cmvar803<=0 and cmvar806>0) → BPsys=min(cmvar800, cmvar806)<br>if (cmvar801>0 and cmvar804<=0 and cmvar807>0) → BPdias=min(cmvar801, cmvar807)<br>if (cmvar800<=0 and cmvar803>0 and cmvar806>0) → BPsys=min(cmvar803, cmvar806)<br>if (cmvar801<=0 and cmvar804>0 and cmvar807>0) → BPdias=min(cmvar804, cmvar807)<br>if (cmvar800<=0 and cmvar803>0 and cmvar806<=0) → BPsys=cmvar803<br>if (cmvar801<=0 and cmvar804>0 and cmvar807<=0) → BPdias=cmvar804<br>if (cmvar800<=0 and cmvar803<=0 and cmvar806>0) → BPsys=cmvar806<br>if (cmvar801<=0 and cmvar804<=0 and cmvar807>0) → BPdias=cmvar807<br>if (cmvar800>0 and cmvar803>0 and cmvar806>0) → BPsys=min(cmvar800, cmvar803, cmvar806)<br>if (cmvar801>0 and cmvar804>0 and cmvar807>0) → BPdias=min(cmvar801, cmvar804, cmvar807)<br><br>**BPsysm/BPdiasm, wave C:**	Variable name:<br>BPsys<br>BPdias<br><br>Variable labels:<br>Lowest blood pressure – systolic (mmHg)<br>Lowest blood pressure – diastolic (mmHg)<br><br>Note: in case there were more than one measurements for a cohort, the mean measure was taken (arm, sitting position) to have more data, as only lowest values were available for B-PROOF. If for some participants the second was not available, the first measure was taken.<br><br>Variable name:<br>BPsysm<br>BPdiasm

			if (cmvar800>0 and cmvar803<=0 and cmvar806<=0) → BPsysm=cmvar800<br>if (cmvar801>0 and cmvar804<=0 and cmvar807<=0) → BPdiasm=cmvar801<br>if (cmvar800>0 and cmvar803>0 and cmvar806<=0) → BPsysm=mean(cmvar800, cmvar803)<br>if (cmvar801>0 and cmvar804>0 and cmvar807<=0) → BPdiasm=mean(cmvar801, cmvar804)<br>if (cmvar800>0 and cmvar803<=0 and cmvar806>0) → BPsysm=mean(cmvar800, cmvar806)<br>if (cmvar801>0 and cmvar804<=0 and cmvar807>0) → BPdiasm=mean(cmvar801, cmvar807)<br>if (cmvar800<=0 and cmvar803>0 and cmvar806>0) → BPsysm=mean(cmvar803, cmvar806)<br>if (cmvar801<=0 and cmvar804>0 and cmvar807>0) → BPdiasm=mean(cmvar804, cmvar807)<br>if (cmvar800<=0 and cmvar803>0 and cmvar806<=0) → BPsysm=cmvar803<br>if (cmvar801<=0 and cmvar804>0 and cmvar807<=0) → BPdiasm=cmvar804<br>if (cmvar800<=0 and cmvar803<=0 and cmvar806>0) → BPsysm=cmvar806<br>if (cmvar801<=0 and cmvar804<=0 and cmvar807>0) → BPdiasm=cmvar807<br>if (cmvar800>0 and cmvar803>0 and cmvar806>0) → BPsysm=mean(cmvar800, cmvar803, cmvar806)<br>if (cmvar801>0 and cmvar804>0 and cmvar807>0) → BPdiasm=mean(cmvar801, cmvar804, cmvar807)<br><br>**BPsys/BPdias, wave 3B:**<br>if (BMARMSS1>0 and BMARMSS2>0) → BPsys=min(BMARMSS1, BMARMSS2)<br>if (BMARMSS2<0 and BMARMSS1>0) → BPsys=BMARMSS1<br>if (BMARMSS1<0 and BMARMSS2>0) → BPsys=BMARMSS2<br><br>if (BMARMDS1>0 and BMARMDS2>0) → BPdias=min(BMARMDS1, BMARMDS2)<br>if (BMARMDS2<0 and BMARMDS1>0) → BPdias=BMARMDS1<br>if (BMARMDS1<0 and BMARMDS2>0) → BPdias=BMARMDS2<br><br>**BPsysm/BPdiasm, wave 3B:**<br>if (BMARMSS1>0 and BMARMSS2>0) → BPsysm=mean(BMARMSS1, BMARMSS2)<br>if (BMARMSS2<0 and BMARMSS1>0) → BPsysm=BMARMSS1<br>if (BMARMSS1<0 and BMARMSS2>0) → BPsysm=BMARMSS2<br><br>if (BMARMDS1>0 and BMARMDS2>0) → BPdiasm=mean(BMARMDS1, BMARMDS2)<br>if (BMARMDS2<0 and BMARMDS1>0) → BPdiasm=BMARMDS1<br>if (BMARMDS1<0 and BMARMDS2>0) → BPdiasm=BMARMDS2	Variable labels: :<br>Mean blood pressure – systolic (mmHg)<br>Mean blood pressure – diastolic (mmHg)
ActiFE Ulm	Blood pressure measured (BP was measured three times, using Blood Pressure Monitor UA-767BT from A&D Medical):<br>Systolic (syst_1_BL, syst_2_BL, syst_3_BL)<br>Diastolic (diast_1_BL, diast _2_BL, diast _3_BL)	Missing: n=5	**BPsys/BPdias**<br>BPsys=min(syst_1_BL, syst_2_BL, syst_3_BL)<br>BPdias=min(diast_1_BL, diast _2_BL, diast _3_BL)<br><br>**BPsysm/BPdiasm**<br>BPsysm=mean(syst_1_BL, syst_2_BL, syst_3_BL)<br>BPdiasm=mean(diast_1_BL, diast _2_BL, diast _3_BL)	

TILDA	Blood pressure measured in duplicate on the upper arm while sitting using an Omron M10-IT, Omron Inc., Kyoto, Japan)<br>Systolic (BPseatedsystolic1, BPseatedsystolic2, BPseateddiastolicmean)<br>Diastolic (BPseateddiastolic1, BPseateddiastolic2, BPseatedsystolicmean)<br><br>Note: standing blood pressure measurements have also been taken.	Range 75-222<br><br><br>Range 46-132	**BPsys**<br>if BPseatedsystolic1>0 and BPseatedsystolic2>0 → BPsys=min(BPseatedsystolic1, BPseatedsystolic2)<br>if sysmis(BPseatedsystolic2) → BPsys=BPseatedsystolic1<br>if sysmis(BPseatedsystolic1) → BPsys=BPseatedsystolic2<br><br>**BPdias**<br>if (BPseateddiastolic1>0 and BPseateddiastolic2>0) BPdias=min(BPseateddiastolic1, BPseateddiastolic2)<br>if sysmis(BPseateddiastolic2) →<br>BPdias=BPseateddiastolic1<br>if sysmis(BPseateddiastolic1) → BPdias=BPseateddiastolic2<br><br>**BPsysm**<br>if BPseatedsystolic1>0 and BPseatedsystolic2>0 → BPsysm=mean(BPseatedsystolic1, BPseatedsystolic2)<br>if sysmis(BPseatedsystolic2) → BPsysm=BPseatedsystolic1<br>if sysmis(BPseatedsystolic1) → BPsysm=BPseatedsystolic2<br><br>**BPdiasm**<br>if (BPseateddiastolic1>0 and BPseateddiastolic2>0) BPdiasm=mean(BPseateddiastolic1, BPseateddiastolic2)<br>if sysmis(BPseateddiastolic2) → BPdiasm=BPseateddiastolic1<br>if sysmis(BPseateddiastolic1) → BPdiasm=BPseateddiastolic2	

B-PROOF	Blood pressure is measured in duplicate using an Omron M1 plus blood pressure device (Omron Healthcare Europe). Sitting or standing?<br>Systolic blood pressure (BP_systolic (lowest systolic blood pressure in mmHg), BP_systolic_1, BP_systolic_2)<br>Diastolic blood pressure (BP_diastolic (lowest diastolic blood pressure in mmHg) BP_diastolic_1, BP_diastolic_2)<br>*NB: BP_systolic_1, BP_diastolic_1, BP_systolic_2 and BP_diastolic_2 are missing for all EMC data at baseline, because at EMC only the lowest systolic blood pressure was reported. Follow-up includes data of all three centers.*<br><br>What is the blood pressure status? (BP_status)<br><br><br><br><br><br><br><br><br><br><br><br><br><br>Blood pressure is measured during vascular measurements, non-invasively in two of the research centres (which?) using a datascoop (which device?):<br>Systolic blood pressure (SBP_datascoop)<br>Diastolic blood pressure (DBP_datascoop)<br>Mean arterial pressure (MAP_datascoop)<br><br>Ambulatory blood pressure monitoring (24-h) is performed using Oscar 2 ambulatory blood pressure monitor (SunTech Medical, North Carolina, USA):<br>Systolic blood pressure (SBP_total_ABPM, SBP_total_ABPM_SD, SBP_day_ABPM, SBP_day_ABPM_SD, SBP_night_ABPM, SBP_night_ABPM_SD)<br>Diastolic blood pressure (DBP_total_ABPM, DBP_total_ABPM_SD, DBP_day_ABPM, DBP_day_ABPM_SD, DBP_night_ABPM, DBP_night_ABPM_SD)<br>Mean arterial pressure (MAP_total_ABPM, MAP_total_ABPM_SD, MAP_day_ABPM, MAP_day_ABPM_SD, MAP_night_ABPM, MAP_night_ABPM_SD)	In mmHg.<br>No information on range.<br>n= 1,798: no data for 1 and 2 (EMC).<br><br>*If both BP measurements are missing the EMC data is taken (=lowest value: BP_systolic or BP_diastolic).<br>>n=557 missing, so for these cases no EMC data is available.<br><br><br><br><br><br><br><br><br><br>Range<br>1 = complete and reliable<br>2 = not reliable and incomplete<br>3 = technical problems<br>4 = not motivated<br>5 = physical limitations<br>6 = pain<br>7 = other limitations<br>8 = participant did not follow instructions<br>9 = measurement not performed<br><br>In mmHg.<br>No information on range.	**BPsys/BPdias**<br>if (BP_systolic>0) → BPsys=BP_systolic<br>if (BP_diastolic>0) → BPdias=BP_diastolic<br><br>**BPsysm/BPdiasm**<br>If (BP_systolic_1 > 0 AND BP_systolic_2 >0) → BPsysm = mean(BP_systolic_1, BP_systolic_2)<br>If SYSMIS(BPsysm) → BPsysm = BP_systolic<br>If (BP_diastolic_1 > 0 AND BP_diastolic_2 >0) → BPdysm = mean(BP_diastolic_1, BP_diastolic_2)<br>If SYSMIS(BPdysm) → BPdysm = BP_diastolic	

Rotterdam Study	Blood pressure was measured at the right brachial artery with the participant in sitting position.<br>Systolic bloodpressure 1 in sitting position (in mmHg) (e5_15637)<br>Diastolic bloodpressure 1 in sitting position (in mmHg) (e5_15638)<br>Systolic bloodpressure 2 in sitting position (in mmHg) (e5_15639)<br>Diastolic bloodpressure 2 in sitting position (in mmHg) (e5_15640)<br><br>Mean of two measurements:<br>Systolic blood pressure (e5_15641)<br>Diastolic blood pressure (e5_15642)	Range<br>888=missing	**BPsys**<br>if e5_15637>0 and e5_15637<888 and e5_15639>0 and e5_15639<888 → BPsys = min(e5_15637, e5_15639)<br>if not missing(e5_15637) and missing(e5_15639) → BPsys = e5_15637<br>if missing(e5_15637) and not missing(e5_15639) → BPsys = e5_15639<br><br>**BPdias**<br>if e5_15638>0 and e5_15638<888 and e5_15640>0 and e5_15640<888 → BPdias = min(e5_15638, e5_15640)<br>if not missing(e5_15638) and missing(e5_15640) → BPdias = e5_15638<br>if missing(e5_15638) and not missing(e5_15640) → BPdias = e5_15640<br><br>**BPsysm**<br>if e5_15641>0 and e5_15641<888 → BPsysm = e5_15641<br><br>**BPdiasm**<br>if e5_15642>0 and e5_15642<888 → BPdiasm = e5_15642	
Body mass index (BMI)				
LASA	BMI calculated from measured height (BMED150, CMED150) and weight (BMED153, CMED153)		**Wave C:**<br>bmi= CMED153/(CMED150/100)^2<br>If CMED150=missing or CMED153=missing → bmi=missing<br><br>**Wave 3B:**<br>bmi= BMED153/(BMED150/100)^2<br>If BMED150=missing or BMED153=missing → bmi=missing	Variable name:<br>bmi<br><br>Variable label:<br>BMI
ActiFE Ulm	BMI (BMI_BL) calculated from measured height (Length_BL) and weight (Weight_BL)	Kg/m^2	Use as is.<br>bmi=BMI_BL	
TILDA	BMI calculated from measured height (height) and weight (weight) and presented in the variable labelled FRbmi	Weight: 45-135 kg<br>Height: 145-185 cm	Can be used as is. Only needs to be renamed.<br>bmi= FRbmi	
B-PROOF	BMI (BMI) calculated from measured height (Length) and weight (Weight)	Kg/m^2	Can be used as is.<br>If bmi<=0 → bmi=missing	
Rotterdam Study	BMI calculated from measured height in cm (e5_229) and weight in kg (e5_230)	Range height:<br>100.0-250.0 cm<br><br>Range weight:<br>30.0-160.0 kg<br><br>999.9=missing<br>888.8=not appropriate-default	If e5_230 >888 or e5_229 >888 → bmi=missing<br>bmi= e5_230 / (e5_229/100)^2	
Cancer				
LASA	Do/did you have: cancer (BCANCER1, CCANCER1)	3B:<br>1=no<br>2=yes<br><br>C:<br>0=no, never<br>1=no, but at wave B yes<br>2=yes, but at wave B no<br>3=yes, and at wave B yes	**Wave C:**<br>If ccancer1=0 → cancer=0<br>If ccancer1=1 or ccancer1=2 or ccancer1=3 → cancer=1<br><br>**Wave 3B:**<br>If BCANCER1=1 → cancer=0<br>If BCANCER1=2 → cancer=1	Variable name:<br>cancer<br><br>Variable label:<br>History of cancer<br><br>Value label:<br>0=no<br>1=yes

ActiFE Ulm	cancer until baseline (C_cancer_BL)	0=no<br>1=yes	Use as is.<br>Cancer= C_cancer_BL	
TILDA	Has a doctor ever told you that you have any of the following conditions?<br>-Cancer or malignant tumor (ph301_05)	0=no<br>1=yes	Use as is.<br>Cancer=ph301_05	
B-PROOF	No data: includes data on adverse events (including cancer) from the follow-up measurements and during follow-up, but about lifetime cancer		Not harmonized.	
Rotterdam Study	Heeft u sinds datum interview ERGO-4/ErgoPlus-2/ErgoJong-1 kanker gehad? (e5_EIMC31)<br>Heeft u ooit kanker gehad? (ep_pimc31)<br>Heeft u sinds (ERGO 3/plus) datum kanker gehad? (e4_dimc31)<br>Heeft u ooit borstkanker gehad? (e3_camama)<br>Heeft u ooit prostaatkanker gehad? (e3_capros)<br>Heeft u ooit dikke darmkanker gehad? (e3_cacol)<br>Ever diagnosed for cancer? (e2_b2casev)	0 = No<br>1 = Yes<br>7 = Don't know<br>9 = No answer	if e5_EIMC31 = 0 or ep_pimc31 = 0 or e4_dimc31 = 0 or e3_camama = 0 or e3_capros = 0 or e3_cacol = 0 or e2_b2casev = 0 → cancer = 0.<br>if e5_EIMC31 = 1 or ep_pimc31 = 1 or e4_dimc31 = 1 or e3_camama = 1 or e3_capros = 1 or e3_cacol = 1 or e2_b2casev = 1 → cancer = 1.	
Cardiovascular disease				
LASA	See appendix 5.			See appendix 5.<br>It makes more sense to use the CVD variables individually as the cohorts differ with respect to the conditions that were reported on.
ActiFE Ulm				
TILDA				
B-PROOF				
Rotterdam study				
Arrhythmia				
LASA	self report surgery: pacemaker (b/c_surARI)<br>use of antiarrhythmics (b/c_mARI)<br>gp diagnosis: cardiac arrhythmia (b/c_gpARI)<br><br>Algorithm for determining presence of arrhythmia (see LASA website for the complete algorithm) (c_alg_ARI / b3_ARI)	-1 = missing<br>0 = no<br>1 = yes<br>2 = possible<br><br>-1 = missing<br>0 = no<br>1 = definitive<br>2 = possible<br>3 = contradictory	**Wave C:**<br>If c_alg_ARI = 0 → arrhythmia = 0<br>If c_alg_ARI = 1 → arrhythmia = 1<br><br>**Wave 3B:**<br>If b3_ARI = 0 → arrhythmia = 0<br>If b3_ARI = 1 → arrhythmia = 1	Variable name:<br>arrhythmia<br><br>Variable label:<br>History of arrhythmia<br><br>Value label:<br>0=no<br>1=yes<br><br>Note that for LASA there was only a self-report for having a pacemaker. Therefore we used LASA's algorithm for determining history of arrhythmia.
TILDA	Has a doctor ever told you that you have any of the following conditions:<br>-angina (ph201_02)<br>-heart attack (ph201_03)<br>-heart failure (ph201_04)<br>-heart murmur (ph201_09)<br>-abnormal heart rhythm (ph201_10)<br>-other heart trouble (ph201_11)	0=no<br>1=yes	Can be used as is. Only needs to be renamed.<br>**arrhythmia=**ph201_10	

B-PROOF	Which type of CVD does participant have? (CVdisease)	1 = arrhythmia<br>2 = angina pectoris<br>3 = myocardial infarction<br>4 = heart failure<br>5 = atrial septum defect<br>6 = pericarditis<br>7 = aneurysm<br>8 = pulmonal hypertension<br><br>678 missing (23,2%)	If CVdisease not 1 → **arrhythmia** =0<br>If CVdisease =1 → **arrhythmia** =1	
ERGO	No data		Not harmonized.	
ActiFE Ulm	No data		Not harmonized.	
Angina pectoris				
LASA	self report: angina pectoris (b3_srAP/c_srAP)<br>gp diagnosis: angina pectoris (b3_gpAP/c_gpAP)<br>use of nitratess (b3_mAP /c_mAP)<br><br><br>Algorithm for determining presence of angina pectoris (c_alg_AP / b3_AP)	-1 = missing<br>0 = no<br>1 = yes<br>2 = possible<br><br>-1 = missing<br>0 = no<br>1 = definitive<br>2 = possible<br>3 = contradictory	**Wave C:**<br>If c_srAP = 0 → anginap= 0<br>If c_srAP = 1 → anginap= 1<br><br>**Wave 3B:**<br>If b3_srAP = 0 → anginap= 0<br>If b3_srAP = 1 → anginap= 1	Variable name:<br>anginap<br><br>Variable label:<br>History of angina pectoris<br><br>Value label:<br>0=no<br>1=yes
TILDA	Has a doctor ever told you that you have any of the following conditions:<br>-angina (ph201_02)<br>-heart attack (ph201_03)<br>-heart failure (ph201_04)<br>-heart murmur (ph201_09)<br>-abnormal heart rhythm (ph201_10)<br>-other heart trouble (ph201_11)	0=no<br>1=yes	anginap= ph201_02	
B-PROOF	Which type of CVD does participant have? (CVdisease)	1 = arrhythmia<br>2 = angina pectoris<br>3 = myocardial infarction<br>4 = heart failure<br>5 = atrial septum defect<br>6 = pericarditis<br>7 = aneurysm<br>8 = pulmonal hypertension	If CVdisease not 2 → anginap =0<br>If CVdisease =2 → anginap =1	
ERGO	No data		Not harmonized.	
ActiFE Ulm	No data		Not harmonized.	

Myocardial infarction				
LASA	self report: myocardial infarction (b3_srMI/c_srMI)<br>gp diagnosis: MI (b3_gpMI/c_gpMI)<br><br><br><br>Algorithm for determining presence of MI (c_alg_MI / b3_MI)	-1 = missing<br>0 = no<br>1 = yes<br>2 = possible<br><br>-1 = missing<br>0 = no<br>1 = definitive<br>2 = possible<br>3 = contradictory	**Wave C:**<br>If c_srMI = 0 → myocardinf = 0<br>If c_srMI = 1 → myocardinf = 1<br><br>**Wave 3B:**<br>If b3_srMI = 0 → myocardinf = 0<br>If b3_srMI = 1 → myocardinf = 1	Variable name:<br>myocardinf<br><br>Variable label:<br>History of myocardial infarction<br><br>Value label:<br>0=no<br>1=yes
TILDA	Has a doctor ever told you that you have any of the following conditions:<br>-angina (ph201_02)<br>-heart attack (ph201_03)<br>-heart failure (ph201_04)<br>-heart murmur (ph201_09)<br>-abnormal heart rhythm (ph201_10)<br>-other heart trouble (ph201_11)	0=no<br>1=yes	myocardinf= ph201_03	
B-PROOF	Which type of CVD does participant have? (CVdisease)	1 = arrhythmia<br>2 = angina pectoris<br>3 = myocardial infarction<br>4 = heart failure<br>5 = atrial septum defect<br>6 = pericarditis<br>7 = aneurysm<br>8 = pulmonal hypertension	If CVdisease not 3 → Myocardinf =0<br>If CVdisease =3 → Myocardinf =1	
ERGO	Heeft u na (datum interview ERGO-4/ErgoPlus-2/ErgoJong-1) wel eens een hartinfarct doorgemaakt ? (e5_EIMI)<br>Heeft u ooit een hartinfarct doorgemaakt? (EP_pimc9)<br>Heeft u na (datum interview ERGO-3/Plus) wel eens een hartinfarct doorgemaakt ? (e4_dimi)<br>Heeft u na (datum interview ERGO-1) wel eens een hartinfarct doorgemaakt ? (e3_cimi)<br>Myocardial infarction after Ergo1? (e2_b1mi)<br>Did you ever experience a heart attack? (e1_aimi)	0=nee<br>1=ja	if e5_EIMI = 0 or EP_pimc9 = 0 or e4_dimi = 0 or e3_cimi = 0 or e2_b1mi = 0 or e1_aim = 0 → myocardinf = 0.<br>if e5_EIMI = 1 or EP_pimc9 = 1 or e4_dimi = 1 or e3_cimi = 1 or e2_b1mi = 1 or e1_aim = 1 → myocardinf = 1.	
ActiFE Ulm	myocardial infarction until baseline (C_myocardinf_BL)	0=no<br>1=yes	myocardinf= C_myocardinf_BL	

Heart failure				
LASA	self reported diagnosis of congestive heart failure (b3_srCHF/c_srCHF)<br>gp diagnosis: congestive heart failure (b3_gpCHF/c_gpCHF)<br>medication for congestive heart failure (b3_mCHF/c_mCHF)<br><br>Algorithm for determing presence of congestive heart failure (c_alg_CHF / b3_CHF)	-1 = missing<br>0 = no<br>1 = yes<br>2 = possible<br><br>-1 = missing<br>0 = no<br>1 = definitive<br>2 = possible<br>3 = contradictory	**Wave C:**<br>If c_srCHF = 0 → heartf = 0<br>If c_srCHF = 1 → heartf = 1<br><br>**Wave 3B:**<br>If b3_srCHF = 0 → heartf = 0<br>If b3_srCHF = 1 → heartf = 1	Variable name:<br>heartf<br><br>Variable label:<br>History of heart failure<br><br>Value label:<br>0=no<br>1=yes
TILDA	Has a doctor ever told you that you have any of the following conditions:<br>-angina (ph201_02)<br>-heart attack (ph201_03)<br>-heart failure (ph201_04)<br>-heart murmur (ph201_09)<br>-abnormal heart rhythm (ph201_10)<br>-other heart trouble (ph201_11)	0=no<br>1=yes	heartf= ph201_04	
B-PROOF	Which type of CVD does participant have? (CVdisease)	1 = arrhythmia<br>2 = angina pectoris<br>3 = myocardial infarction<br>4 = heart failure<br>5 = atrial septum defect<br>6 = pericarditis<br>7 = aneurysm<br>8 = pulmonal hypertension	If CVdisease not 4 → heartf =0<br>If CVdisease =4 → heartf =1	
ERGO	No data		Not harmonized	
ActiFE Ulm	heart failure until baseline (C_heartf_BL)	0=no<br>1=yes	heartf= C_heartf_BL	
Cohort				
LASA C			cohort=1	Variable name:<br>cohort<br><br>Value labels:<br>1=LASA C<br>2=LASA 3B<br>3=ActiFE Ulm<br>4=TILDA<br>5=B-PROOF<br>6=Rotterdam study
LASA 3B			cohort=2	
ActiFE Ulm			cohort=3	
TILDA			cohort=4	
B-PROOF			cohort=5	
Rotterdam study			cohort=6	

**Creatinine**				
LASA	Blood/Serum: creatinine uMOL/L (cmcreati/bmcreat)	-1 = missing	**Wave C:**<br>If (cmcreati>0) creatinine = cmcreati<br>GFRe variable created.<br><br>**Wave 3B:**<br>If (bmcreat>0) creatinine = bmcreat<br>GFRe variable created.	Variable name:<br>1. creatinine<br>2. eGFR<br><br>Variable label:<br>Creatinine (µmol/l)<br>eGFR (Cockcroft and Gault formula)<br><br>GFR can be estimated as follows:<br>eGFR = ((140 – age (years)) × weight (kg) × (1.23male + 1.05females)) /serum creatinine(µmol/L)<br><br>Note: The Cockcroft and Gault formula is generally not considered to be the most accurate formulate for estimating GFR since it does not take into account ethnicity. However, most cohorts lack data on ethnicity.
ActiFE Ulm	Serum creatinine (µmol/l) (LAB_Creatinine_BL)		LAB_Creatinine_BL=creatinine<br><br>GFRe variable created.	
TILDA	No data in public dataset.		Not harmonized.	
B-PROOF	Serum creatinine level (micromol/l) (Creatinine)		Use as is.<br><br>GFRe variable created.	
Rotterdam study	Creatinine in serum (µmol/l) (e5_15751)	8888=not applicable/default	e5_15751=creatinine<br><br>GFRe variable created.	
**CRP level**				
LASA	cmhscrp, bmhscrp	Range (in mg/L)<br>-2= no data, n.a.<br>-1= no valid data	**Wave C:**<br>If (cmhscrp>0)CRP = cmhscrp<br><br>**Wave 3B:**<br>If (bmhscrp >0) CRP = bmhscrp	Variable name:<br>CRP<br><br>Variable label:<br>CRP (mg/l)
ActiFE Ulm	LAB_CRP_BL	Range (in mg/l)	Use as is.<br>CRP = LAB_CRP_BL	
TILDA	CRP_W1	Range (in mg/l)	Use as is.<br>CRP = CRP_W1	
B-PROOF	CRP	Range (in mg/l)	Use as is.	
Rotterdam study	No data available.		Not harmonized.	
**Depression**				
LASA	20-item Center for Epidemiologic Studies Depression Scale (CES-D) (bcesdint, ccesdint)<br><br>Reference:<br>Radloff LS, Teri L. Use of the CES-D with older adults. Clin Gerontol. 1986; 5, 119-36.	Range 0-60<br>(scores >16 are indicative of clinical depression)	**Wave C:**<br>If ccesdint <16 → depression=0<br>If ccesdint >=16 → depression=1<br>Zdepression variable created<br><br>**Wave 3B:**<br>If bcesdint <16 → depression=0<br>If bcesdint >=16 → depression=1<br>Zdepression variable created	Variable name:<br>1. depression<br>2. zdepression<br><br>Variable labels:<br>1. Clinical depression (Yes/No)<br>2. Z-score depression<br><br>Value label depression:<br>0=no clinically relevant depression

ActiFE Ulm	HADS-Depression (HADS_D_score_BL)<br><br>Cut-off score of 8 appears to be optimal, and is commonly used.<br><br>Reference:<br>Bjelland, L. The validity of the Hospital Anxiety and Depression Scale. An updated literature review. Journal of Psychosomatic Research. 2002; 52 (2), 69-77	Range 0-21	If HADS_D_score_BL < 8 → depression = 0<br>If HADS_D_score_BL >= 8 → depression = 1<br><br>Zdepression variable created	1=clinically relevant depression<br><br>Ref CESD cut-offs:<br>https://edge.edx.org/assets/courseware/v1/f617df004b9ff814249a98b79773cd6e/asset-v1:GeorgetownX+CCHD+2016+type@asset+block/CESD.pdf<br>Ref GDS:<br>https://econtent.hogrefe.com/doi/full/10.1024/1662-9647/a000101<br><br><br>Note: in accordance with total scores constructed in TILDA and LASA; when one of the items was missing, no total cesd score was developed for ERGO (same approach as for HADS-Anxiety).
TILDA	20-item Center for Epidemiologic Studies Depression Scale (CES-D) (MHcesd_capi)	Range 0-60<br>(scores >16 are indicative of clinical depression)	If MHcesd_capi<16 → depression=0<br>If MHcesd_capi>=16 → depression=1<br><br>Zdepression variable created	
B-PROOF	Geriatric Depression Scale (GDS) is used (GDS_score)<br><br><br><br><br><br>Sub items from SF and Euroqol scales about depression:<br>Euroqol_5 (mood)<br><br><br><br><br><br><br><br><br><br>SF12_11: How much time during the past 4 weeks have you felt down?	Score GDS, Range 0-15<br>Score >= 6 possible/clinically relevant depression<br><br>1 = not depressed/scared<br>2 = a bit depressed/scared<br>3 = severe depressed/scared<br><br>1 = all of the time<br>2 = most of the time<br>3 = a good bit of the time<br>4 = some of the time<br>5 = a little of the time<br>6 = none of the time	If GDS_score<6 → depression=0<br>If GDS_score>=6 → depression=1<br><br>Zdepression variable created	

Rotterdam Study	Subjects are screened with the Center for Epidemiologic Studies Depression Scale (CESD) during the home interview (20 items). (e5_EICESD01… e5_EICESD20)<br><br><br><br><br><br><br><br><br><br><br><br><br><br><br><br>Another questionnaire used is based on the Hospital Anxiety and Depression Scale (HADS), with the aim to assess depressive disorders:<br>I feel tense lately. (e5_EIHADS01)<br><br><br><br><br>I get an anxious feeling that something terrible will happen any moment. (e5_EIHADS03)<br><br><br><br><br><br>I am worried lately. (e5_EIHADS05)<br><br><br><br><br><br><br>I can sit peacefully and relax lately. (e5_EIHADS07)<br><br><br><br><br><br><br>Lately I get sort of a tense feeling in M (e5_EIHADS09)<br><br><br><br><br><br><br>I feel restless lately (e5_EIHADS11)	Range 0-60<br>(scores >16 are indicative of clinical depression)<br><br>Answer categories:<br>0 = seldom or never (0-1 da y)<br>1 = sometimes (1-2 days)<br>2 = regularly (3-4 days)<br>3 = most of the time (5-7 days)<br>7 = don't know<br>9 = no answer<br><br>0 = most of the time<br>1 = often<br>2 = sometimes<br>3 = never<br>7 = don't know<br>9 = no answer<br><br>0 = very true<br>1 = yes, but not that much<br>2 = a little, no concerns<br>3 = not at all<br>7 = don't know<br>9 = no answer<br><br>0 = very often<br>1 = often<br>2 = now and then<br>3 = seldom or never<br>7 = don't know<br>9 = no answer<br><br>0 = certainly<br>1 = most of the time<br>2 = not often<br>3 = not true<br>7 = don't know<br>9 = no answer<br><br>0 = not true<br>1 = sometimes<br>2 = regularly<br>3 = very often<br>7 = don't know<br>9 = no answer<br><br>0 = very much<br>1 = quite a lot<br>2 = not that much<br>3 = not true<br>7 = don't know<br>9 = no answer<br><br>0 = very often<br>1 = regularly	For items 1-3, 5-7, 9-11, 13-15, 17-20 the scoring is 0 → 3.<br>For items 4, 8, 12 and 16 the scoring is 3 → 0 (reverse).<br><br>First, the four items are recoded, next, the scores of all items are added up (cesd_score).<br><br>if (cesd_score<16) depression=0.<br>if (cesd_score>=16) depression=1.<br><br><br>*For n=416 >> at least one of the single CESD items is missing.*<br>*In total n=8196 missing >> no CESD score (of in total n=14926 cases).*<br><br>Zdepression variable created	

	Lately I get sudden feelings of anxiety or panic (e5_EIHADS13)	2 = not very often<br>3 = not true<br>7 = don’t know<br>9 = no answer		

Diabetes				
LASA	Have you been diagnosed with: diabetes (BDIABE01, CDIABE01)	C:<br>0=no, never<br>1=no, but at wave B yes<br>2=yes, but at wave B no<br>3=yes, and at wave B yes<br><br>3B:<br>1=no<br>2=yes	**Wave C:**<br>If cdiabe01=0 → diabetes=0<br>If cdiabe01=1 or cdiabe01=2 or cdiabe01=3 → diabetes=1<br><br>**Wave 3B:**<br>If BDIABE01=1 → diabetes=0<br>If BDIABE01=2 → diabetes=1	Variable name:<br>diabetes<br><br>Value labels:<br>0=no<br>1=yes
ActiFE Ulm	diabetes until baseline (C_diab_BL)	0=no<br>1=yes	Use as is.<br>diabetes = C_diab_BL	
TILDA	Has a doctor ever told you that you have any of the following conditions: diabetes or high blood sugar (ph201_05)	0=no<br>1=yes	Use as is.<br>Ph201_05=diabetes	
B-PROOF	Does the participant have diabetes? (diabetes)	0 = no<br>1 = yes<br><br>662 missing responses (22.7%)	Use as is.	
Rotterdam Study	Do you have diabetes? (e5_EIDM)	0 = No<br>1 = Yes<br>7 = don't know<br>9 = no answer	if e5_EIDM < 7 → diabetes= e5_EIDM	
Dizziness				
LASA	Dizzy: regular? (BMVAR700, CMVAR700)<br><br>If yes, the following follow-up questions were asked:<br>-dizzy when getting up? (BMVAR701, CMVAR701)<br>-dizzy when turning head? (BMVAR702, CMVAR702)<br>-dizzy when looking up? (BMVAR703, CMVAR703)<br>-dizzy, other? (BMVAR704, CMVAR704)	1=no<br>2=yes<br><br>0=not mentioned<br>1=mentioned	**Wave C:**<br>If CMVAR700=1 → dizzy=0<br>If CMVAR700=2 → dizzy=1<br><br>**Wave 3B:**<br>If BMVAR700=1 → dizzy=0<br>If BMVAR700=2 → dizzy=1	Variable name:<br>dizzy<br><br>Variable label:<br>Regular dizziness<br><br>Value labels:<br>0=no<br>1=yes
ActiFE Ulm	dizziness (dizziness_BL)	0=none<br>1=rare<br>2=sometimes<br>3=frequently<br>4=permanent)	dizziness_BL=0 or dizziness_BL=1 → dizzy=0<br>dizziness_BL=2 or dizziness_BL=3 or dizziness_BL=4 → dizzy=1	
TILDA	No data available		Not harmonized.	
B-PROOF	No data available		Not harmonized.	

Rotterdam Study	Are you dizzy often? (dcam20)<br><br>Are you ever dizzy? (e5_EIKNO6)<br><br>Do you get dizzy without moving, i.e. when sitting still, standing still, or lying still? (e5_EIKNO6B1)<br>Do you get dizzy when: moving your head? (e5_EIKNO6B2)<br>Do you get dizzy when: watching moving images (i.e. on film, computer etc.)? (e5_EIKNO6B3)<br>Do you get dizzy when: getting up? (e5_EIKNO6B4)<br>Do you get dizzy when: looking up? (e5_EIKNO6B5)<br>Do you get dizzy when: turning in bed? (e5_EIKNO6B6)<br>Do you get dizzy when: other? (e5_EIKNOnumbe rof 6B7)<br><br>In case of other, in what situations do you get dizzy? (e5_EIKNO6B8)	0 = not or seldom<br>1 = >1 times a week<br>8 = no answer/don't know<br>9 = not applicable/missing<br><br>0 = no<br>1 = almost never<br>2 = sometimes<br>3 = yes, all the time<br>7 = don't know<br>9 = no answer<br><br>0 = no<br>1 = yes<br>7 = don't know<br>8 = n.a.<br>9 = no answer	If e5_EIKNO6 >= 2 and e5_EIKNO6 <= 4 → dizzy=1<br>If e5_EIKNO6 = 0 or e5_EIKNO6 = 1 → dizzy=0	
Education				
LASA	Education level attained (aeducat)	1=elementary not completed (5 yrs)<br>2=elementary (6 yrs)<br>3=lower vocational (9 yrs)<br>4=general intermediate (10 yrs)<br>5=intermediate vocational (11 yrs)<br>6=general secondary (12 yrs)<br>7=higher vocational (15 yrs)<br>8=college (16 yrs)<br>9=university (18 yrs)	If aeducat=1 or aeducat=2 or aeducat=3 or aeducat=4 → edu =1<br>If aeducat=5 or aeducat=6 → edu =2<br>If aeducat=7 or aeducat=8 or aeducat=9 → edu =3	Variable name:<br>Edu<br><br>Variable label:<br>Education level<br><br>Value labels:<br>1= Low (ISCED level 0,1 and 2)<br>2= Average (ISCED level 3 and 4)<br>3= High (ISCED level 5 through 8)<br><br>Education was harmonised in accordance with the ISCED 2011 mappings.
ActiFE Ulm	education reached (education)	1=no graduation<br>2=9 years<br>3=10-11 year<br>4>=12 years<br>5=University	If education=1 or education=2 or education=3→ edu =1<br>If education=4 → edu =2<br>If education=5 → edu =3	

TILDA	What is the highest level of education you have completed? (dm001)	1=some primary (not complete)<br>2=primary or equivalent<br>3=intermediate/junior/group cert<br>4=leaving cert or equivalent<br>5=diploma/cert<br>6=primary degree<br>7=postgraduate/higher degree<br>96=none	If dm001=96 or dm001=1 or dm001=2 or dm001=3 → edu =1<br>If dm001=4 → edu =2<br>If dm001=5 dm001=6 or dm001=7 → edu =3	
B-PROOF	What is the highest level of education completed? (education)<br>How many years of education (dependent on previous variable)? (Edu_years)	1 = primary school (5 yrs)<br>2 = lower education (6 yrs)<br>3 = MULO, ULO, MAVO (9 yrs)<br>4 = secondary education (10 yrs)<br>5 = MMS, HBS, Lyceum, Atheneum, Gymnasium to 3rd year (included) (11 yrs)<br>6 = MMS, HBS, Lyceum, Atheneum, Gymnasium completed (12 yrs)<br>7 = higher education (15 yrs)<br>8 = university or college, until examination (16 yrs)<br>9 = university or college, fully completed (18 yrs)	If education=1 or education=2 or education=3 or education=4 or education=5 → edu =1<br>If education=6 → edu =2<br>If education=7 or education=8 or education=9 → edu =3	
Rotterdam Study	Highest level of education RS-I-1 (recoded), RS-II-1 and RS-III-1 – UNESCO classification (ses_UNESCO_recode)	0 = primary education (5 yrs)<br>1 = lower vocational/intermediate general education (5-9 yrs)<br>2 = intermediate vocational education OR general secondary education (10-14 yrs)<br>3 = higher vocational education (15-18 yrs)<br>99 = missing	If ses_UNESCO_recode=0 or ses_UNESCO_recode=1→ edu=1<br>If ses_UNESCO_recode=2 → edu=2<br>If ses_UNESCO_recode=3 → edu=3	
Executive function				
LASA	Verbal fluency*<br>-words with letter 'D' (bmDWcor)<br>-animal naming (bmAcor)<br><br>***Executive functioning was only measured in wave 3B.**	No. of words correct:<br>Range 0-30<br>Range 0-50	**semanticfl**<br>semanticfl =bmAcor<br><br>**zverbfl**<br>Create Z-score ZbmAcor;<br>ZbmAcor=zverbfl	Variable name:<br>zverbfl<br><br>Variable label:<br>Z-score verbal fluency<br><br>zverbfl is a combination of both semantic (LASA, TILDA, ERGO, ActiFE Ulm) and phonetic (B-PROOF) fluency and includes all cohorts, as a Z-score is calculated.
ActiFE Ulm	Verbal fluency animals (0-15 sec) (fluency1_BL)<br>Verbal fluency animals (15-30 sec) (fluency2_BL)<br>Verbal fluency animals (30-45 sec) (fluency3_BL)<br>Verbal fluency animals (45-60 sec) (fluency4_BL)	No. of named animals:<br>Range 0 – 19	**semanticfl**<br>semanticfl = fluency1_BL + fluency2_BL + fluency3_BL + fluency4_BL<br><br>Create Z-score of summed fluency scores<br>zverblf	

TILDA	Colour trail 2 test<br>Verbal fluency (ph125)<br>Visual reasoning	Range 0-50	semanticfl=ph125<br><br>Create Z-score Zph125;<br>Zph125=zverbfl	
B-PROOF	Trail Making Test (TMT) item A en B (Time_sec_TMT_A, TMT_A_corrections, Time_sec_TMT_B, TMT_B_corrections)<br><br>Stroop colour test (card 1 (words), 2 (colours), 3 (words & colours) (Time_sec_Stroop_Card1, Stroop_Card1_corrections, Time_sec_Stroop_Card2, Stroop_Card2_corrections, Time_sec_Stroop_Card3, Stroop_Card3_corrections)<br>Letter Fluency test (items named with first, second, third letter, and mean) (Let_flu_tot_1st_letter, Let_flu_tot_2nd_letter, Let_flu_tot_3rd_letter, Fluency_mean)	Time in seconds spent on test (max. 300 s) and # of corrections made by interviewer<br><br>Time in seconds spent on test (max. 300 s) and # of corrections made by interviewer<br><br># items named (range 0-30)	Create Z-score ZFluency_mean<br>ZFluency_mean=zverbfl	
Rotterdam Study	Number of attempts fluency task (e5_2751)<br>Correct answers fluency task (e5_2750)	Range<br>= 0 - 50<br>99 = missing<br>88 = not applicable/default	semanticfl=e5_2750<br><br>Create Z-score zverbfl	
Faller in past 12 months				
LASA	Did you fall in the past year? (BMVAR706, CMVAR706)<br><br>How often did you fall in the past year? (BMVAR707, CMVAR707)	1=no<br>2=yes<br><br>3B: Range 1-20<br>C: Range 1-100	**faller**<br>If CMVAR706/BMVAR706=1 → faller=0<br>If CMVAR706/BMVAR706=2 → faller=1<br><br>**Nfalls1**<br>If CMVAR706/BMVAR706=1 → nfalls1=0<br>If CMVAR707/BMVAR707=1 → nfalls1=1<br>If CMVAR707/BMVAR707>=2 → nfalls1=2<br><br>**Nfalls2**<br>If CMVAR706/BMVAR706=1 → nfalls2=0<br>If CMVAR707/BMVAR707=1 or CMVAR707/BMVAR707=2 → nfalls2=1<br>If CMVAR707/BMVAR707>=3 & CMVAR707/BMVAR707<=9 → nfalls2=2<br>If CMVAR707/BMVAR707>=10 → nfalls2=3	Variable name:<br>1. faller<br>2. nfalls1<br>3. nfalls2<br><br>Variable label:<br>1. Fall in the past 12 months<br>2. Number of falls in the past 12 months (0=none, 1=1 fall, 2=2+ falls)<br>3. Number of falls in the past 12 months (0=none, 1=1-2 times, 2=3-9 times, 3=10+ times)<br><br>Value label:<br>faller:<br>0=no<br>1=yes<br><br>Nfalls1:<br>0=none<br>1=1 fall<br>2=2+ falls

ActiFE Ulm	fall within 12 months prior to baseline (FH_fall_12_BL)<br><br>frequency of falling within 12 months prior to baseline [fall history] (FH_fall_12_freq_imp3_BL)<br>Note that FH_fall_12_freq_imp3_BL includes data from another item related to falls in the last 3 months before baseline.	0=no<br>1=yes<br><br>Range 1-300	Use as is.<br>faller = FH_fall_12_BL<br><br>**Nfalls1:**<br>if FH_fall_12_BL = 0 → nfalls1 = 0<br>if FH_fall_12_freq_imp3_BL = 1 → nfalls1 = 1<br>if FH_fall_12_freq_imp3_BL >= 2 → nfall1 = 2<br><br>**Nfalls2:**<br>If FH_fall_12_BL = 0 → nfalls2=0.<br>If FH_fall_12_freq_imp3_BL = 1 or FH_fall_12_freq_imp3_BL = 2 → nfalls2 = 1<br>If FH_fall_12_freq_imp3_BL >=3 & FH_fall_12_freq_imp3_BL <=9 → nfalls2=2<br>If FH_fall_12_freq_imp3_BL >=10 → nfalls2=3	Nfalls2:<br>0=none<br>1=1-2 times<br>2=3-9 times<br>3=10+ times<br><br>Note: no data on no. of falls >2 for B-PROOF. Also, nfalls2 could not be generated for ERGO.
TILDA	Any fall in the past year (ph401)<br><br>Number of falls in the past year (ph402)<br>(only asked if responded 'yes' to ph401)	1=yes<br>5=no<br><br>Range 1-10<br>10 = 10+ falls	**Faller:**<br>If ph401=5 → faller=0<br>If ph401=1 → faller=1<br><br>**Nfalls1:**<br>if ph401=5 → nfalls1=0<br>if ph402=1 → nfalls1=1<br>if ph402>=2 → nfall1=2<br><br>**Nfalls2:**<br>If ph401=5 → nfalls2=0.<br>If ph402=1 or ph402=2 → nfalls2=1<br>If ph402>=3 & ph402<=9 → nfalls2=2<br>If ph402>=10 → nfalls2=3	
B-PROOF	How often did you fall in the past 12 months? (falls_frequency)	Fall frequency 12 months before baseline:<br>0 = no falls<br>1 = one fall<br>2 = two or more falls	**Faller**<br>If falls_frequency=0 → faller=0<br>If falls_frequency=1 or falls_frequency=2 → faller=1<br><br>**Nfalls1**<br>falls_frequency=nfalls1<br><br>**Nfalls2**<br>No data available.	
Rotterdam Study	Did you fall in the past 12 months? (If yes, ask): How often did you fall in the past 12 months? (e5_EIMC27)	0 = no<br>1 = yes, < 1 time per month<br>2 = yes, >=1 times per month,<br>3 = yes, >= 1 times per week<br>4 = daily<br>7 = don't know<br>9 = no answer	**Faller**<br>If e5_EIMC27=0 → faller=0<br>If e5_EIMC27=1 or e5_EIMC27=2 or e5_EIMC27=3 or e5_EIMC27=4 → faller=1<br><br>Nfalls cannot be harmonized.	

Falls in Follow-up (time to first fall)				
LASA	LASA 3B no data<br><br>In addition to the retrospective falls, during LASA C, a three-year prospective fall calendar was used. At the end of LASA wave C (1995/1996), respondents received a 'fall calendar' and were asked to record fall events weekly until the medical interview at wave D (1998/1999). Participants were instructed to record weekly whether or not they had fallen and if they had fallen inside or outside. Subsequently, they were asked to mail the calendar to the institute at the end of every three-month period. They were contacted by telephone if they were not able to complete the 'fall calendar', if no calendar was returned even after a reminder, or if the calendar was completed incorrectly. Proxies were contacted if participants were not able to respond. For the respondents, a fall was defined as 'an unintentional change in position resulting in coming to rest at a lower level or on the ground' (LASACo00/01/02).<br><br>status first fall: yes/no (fall1)<br>Time to first fall in weeks (TTF1)<br>Fall this week (week001, week002, …)<br><br>Note that values for TTF1 do not necessarily indicate a fall occurred. If fall1=0, then TTF1 represents the observation period. If fall1=1, then TTF1 represents the time until first fall.	-2 = Not in study anymore<br>0 = No<br>1 = Yes	**fallyearone**<br>if fall1 = 0 → fallyearone=0<br>if fall1 = 1 and TTF1 >52→ fallyearone=0<br>if fall1 = 1 and TTF1 <=52 →fallyearone=1<br><br>**timetofirstfall**<br>TTF1 = timetofirstfall<br><br>**followup**<br>Follow-up variable created that denotes the number of weeks of follow-up, up until 52 weeks.<br><br>**recfallsyearone**<br>If week052 = -2 → recfallsyearone= missing<br>If week052 >= 0 → recfallsyearone= SUM(week001, week002, week003, week004, week005, week006, week007, week008, week009, week010, week011, week012, week013, week014, week015, week016, week017, week018, week019, week020, week021, week022, week023, week024, week025, week026, week027, week028, week029, week030, week031, week032, week033, week034, week035, week036, week037, week038, week039, week040, week041, week042, week043, week044, week045, week046, week047, week048, week049, week050, week051, week052)<br><br>If recfallsyearone < 2 → recfallsyearone = 0<br>If recfallsyearone >= 2 → recfallsyearone =1<br>If recfallsyearone = 0 and followup < 52 → recfallsyearone = missing	Variable name:<br>1. fallyearone<br>2. timetofirstfall<br>3. followup<br>4. recfallsyearone<br><br>Variable labels:<br>1. Fall within first year of follow-up<br>2. Time to first fall in weeks<br>3. followup<br>4. Two or more falls within first year of follow-up<br><br>Values labels:<br>fallyearone<br>0=no<br>1=yes<br><br>Note that the week number is used for timetofirstfall. For example, if a respondent fell two days after baseline, timetofirstfall would equal 1.<br><br>Note that fallyearone is intended for survival analyses. If the intention is to use it as a dichotomous outcome measure without the time variable, then fallyearone should be coded as missing if both the follow-up was shorter than 12 months and no fall was recorded for the patient within the follow-up period.
ActiFE Ulm	Falls were prospectively measured over 53 weeks after baseline.<br><br>Start of fall calender documentation (fall_cal_start_BL)<br><br>Date of first fall within the observation period following baseline (FC_first_fall_date_BL)<br><br>Observation period (days) of falls following baseline (FC_fall_obsdays_BL)<br><br>Frequency of falling within the observation period following baseline [fall calendar] (FC_fall_freq_BL)		**Timetofirstfall & fallyearone**<br>if FC_first_fall_date_BL=missing and fall_cal_start_BL=not missing → fallyearone = 0<br>if FC_first_fall_date_BL=not missing and fall_cal_start_BL=not missing → fallyearone = 1<br>timetofirstfall= round up((FC_first_fall_date_BL - fall_cal_start_BL) / 7)<br>if timetofirstfall= 53 → fallyearone = 0<br>if fallyearone = 0 → timetofirstfall = round up(FC_fall_obsdays_BL / 7)<br>if timetofirstfall=53 → timetofirstfall=52<br>if timetofirstfall = 0 → timetofirstfall = 1<br><br>**followup**<br>followup = round((FC_fall_obsdays_BL/7))<br><br>**recfallsyearone**<br>If FC_fall_freq_BL = 0 OR FC_fall_freq_BL = 1 → recfallsyearone = 0<br>If FC_fall_freq_BL > 1 → recfallsyearone = 1<br>If recfallsyearone = 0 AND followup < 52 → recfallsyearone = missing	
TILDA	no data		Not harmonized.	

B-PROOF	Besides the retrospective falls, falls were also measured during the follow-up (2-3 years), using a fall calendar:<br>Falling during follow-up (FU1_Falls)<br><br>Date first, second, third, fourth, fifth fall (FU1_Date_fall1 … FU1_Date_fall5)<br><br>FU: Time since baseline to first fall (days) (FU1_Time_to_first_fall)<br><br>end data 2 or fall date, thus sensor data, used for the follow-up time (end_date_for_FU)<br><br>Date of baseline interview (Date_interview)	0= no<br>1= yes<br><br><br><br><br><br>Date (only including falls within a 12 months from the baseline measurements)	**timetofirstfall**<br>if FU1_Falls=1 → timetofirstfall= round up(FU1_Time_to_first_fall / 7)<br>if FU1_Falls=0 → timetofirstfall= round up(end_date_for_FU - Date_interview)/7)<br>if timetofirstfall = 0 → timetofirstfall = 1<br><br>**followup**<br>Follow-up variable created that denotes the number of weeks of follow-up, up until 52 weeks.<br><br>**fallyearone**<br>fallyearone= FU1_Falls<br>If timetofirstfall > 52 → fallyearone = 0<br>If timetofirstfall > 52 → timetofirstfall = 52<br><br>**recfallsyearone**<br>timetosecfall = RND((DATEDIFF(FU1_Date_fall2, Date_interview, 'days')/7) + 0.4999)<br>if timetosecfall <= 52 → recfallsyearone=1<br>if timetosecfall > 52 → recfallsyearone=0<br>if (missing(timetosecfall) AND followup >= 52) recfallsyearone = 0.<br>if (missing(timetosecfall) and followup < 52) recfallsyearone=$sysmis.	
Rotterdam Study	Serious falls (i.e. a fall leading to a hospital admission or leading to a fracture) were measured prospectively. Serious fall data were obtained from a computerized reporting system of the general practitioners within the Rotterdam Study.		Not harmonized.	
Fear of falls				
LASA	No data available from LASA 3B.<br><br>LASA C: modified version of the Falls Efficacy Scale (FES) was used. In LASA, the participant was asked to score how concerned he/she felt to fall during 10 activities of daily living:<br>-Cleaning the house (cmvar301)<br>-Dress and undress yourself (cmvar302)<br>-The preparation of simple meals (cmvar303)<br>-Taking a bath or a shower (cmvar304)<br>-Do some shopping (cmvar305)<br>-To get in and out of a chair (cmvar306)<br>-To climb up and down the stairs (cmvar307)<br>-A small walk in the neighbourhood (cmvar308)<br>-Deep or low cupboard (cmvar309)<br>-Answering the phone before it stops (cmvar310)	Range 0-5:<br>1=not concerned<br>2=a little concerned<br>3=fairly concerned<br>4=very concerned<br>5=R does not or cannot	**Fearfall**<br>If (cmvar301=1 or cmvar301=5) and (cmvar302=1 cmvar302=5) and (cmvar303=1 or cmvar303=5) and (cmvar304=1 or cmvar304=5) and (cmvar305=1 or cmvar305=5) and (cmvar306=1 or cmvar306=5) and (cmvar307=1 or cmvar307=5) and (cmvar308=1 or cmvar308=5) and (cmvar309=1 or cmvar309=5) and (cmvar310=1 or cmvar310=5) → fearfall=0<br>If (cmvar301=2 or cmvar301=3) or (cmvar302=2 or cmvar302=3) or (cmvar303=2 or cmvar303=3) or ((cmvar304=2 or cmvar304=3) or ((cmvar305=2 or cmvar305=3) or ((cmvar306=2 or cmvar306=3) or ((cmvar307=2 or cmvar307=3) or ((cmvar308=2 or cmvar308=3) or ((cmvar309=2 or cmvar309=3) or ((cmvar310=2 or cmvar310=3) → fearfall=1<br>If cmvar301=4 or cmvar302=4 or cmvar303=4 or cmvar304=4 or cmvar305=4 or cmvar306=4 or cmvar307=4 or cmvar308=4 or cmvar309=4 or cmvar310=4 → fearfall=2.	Variable name:<br>fearfall<br><br>Variable label:<br>Fear of falling<br><br>Value label:<br>0=not afraid<br>1=somewhat afraid<br>2=very afraid<br><br>For LASA & ActiFE Ulm it was chosen to code fearfall=0 when all activities are rated as not concerned, fearfall=1 when at least one of the activities is rated as a little/fairly concerned

ActiFE Ulm	FES-I SF7:<br>1. Getting dressed or undressed (fesi_1_BL)<br>2. Taking a bath or shower (fesi_2_BL)<br>3. Getting in or out of a chair (fesi_3_BL)<br>4. Going up or down stairs (fesi_4_BL)<br>5. Reaching for something above your head or on the ground (fesi_5_BL)<br>6. Walking up or down a slope (fesi_6_BL)<br>7. Going out to a social event (e.g. religious service, family gathering or club meeting) (fesi_7_BL)<br><br>Sum score (fesi_BL)<br>https://www.ncbi.nlm.nih.gov/pubmed/18032400?dopt=Abstract<br><br>Score of >10 indicates high concern about falling<br>https://academic.oup.com/ageing/article/39/2/210/40898	Range 0-4:<br>1=not at all concerned<br>2=somewhat concerned<br>3=fairly concerned<br>4=very concerned<br><br>Range 7 – 28	**fearfall**<br>if fesi_1_BL=1 and fesi_2_BL=1 and fesi_3_BL=1 and fesi_4_BL=1 and fesi_5_BL=1 and fesi_6_BL=1 and fesi_7_BL=1 → fearfall = 0<br>if (fesi_1_BL= 2 or fesi_1_BL=3) or (fesi_2_BL= 2 or fesi_2_BL=3) or (fesi_3_BL= 2 or fesi_3_BL=3) or (fesi_4_BL= 2 or fesi_4_BL=3) or (fesi_5_BL= 2 or fesi_5_BL=3) or (fesi_6_BL= 2 or fesi_6_BL=3) or (fesi_7_BL= 2 or fesi_7_BL=3) → fearfall = 1<br>if fesi_1_BL=4 or fesi_2_BL=4 or fesi_3_BL=4 or fesi_4_BL=4 or fesi_5_BL=4 or fesi_6_BL=4 or fesi_7_BL=4 → fearfall = 2	(and none of the other activities as >fairly concerned), fearfall=2 when at least one of the activities is rated as very concerned.
TILDA	Are you afraid of falling? (ph408)<br><br>If yes to ph408, do you feel somewhat afraid or very much afraid of falling? (ph409)	1=yes<br>5=no<br><br>1=somewhat afraid<br>2=very afraid	if ph408=5 → fearfall=0.<br>if ph409=1 → fearfall=1.<br>if ph409=2 → fearfall=2.	
B-PROOF	No data available		Not harmonized.	
Rotterdam Study	No data available		Not harmonized.	
Functional limitations / Activities of daily living (see appendix 6)				
LASA	-**Can you walk up and down a staircase of 15 steps without resting**? (BADL1A,CADL1A)<br>-**Can you dress and undress yourself?** (BADL2A, CADL2A)<br>-**Can you sit down and stand up from a chair?** (BADL3A, CADL3A)<br>-**Can you cut your own toenails?** (BADL4A, CADL4A)<br>-**Can you walk outside during five minutes without stopping?** (BADL5A, CADL5A)<br>-Can you use your own or public transportation? (BADL6A, CADL6A)<br><br>**3B: addition:**<br>-Can you take a bath/shower? (BADL7A)	1=No, I cannot<br>2= Only with help<br>3= Yes, with much difficulty<br>4= Yes, with some difficulty<br>5= Yes, without help	**funlimP**<br>Calculate number of limitations by adding BADL1A 1 point for each 'yes with some difficulty', 'yes with much difficulty', 'only with help' or 'no I cannot' to the six items:<br>funlimp= BADL1A+BADL2A+ BADL3A+ BADL4A+ BADL5A+ BADL6A+ BADL7A<br><br>**funlim5**<br>If BADL1A >=1 & BADL1A<=4 → funlim5=funlim5+1<br>If (BADL2A >=1 & BADL2A<=4) → funlim5=funlim5+1<br>If BADL4A >=1 & BADL4A<=4 → funlim5=funlim5+1<br>If BADL5A >=1 & BADL5A<=4 → funlim5=funlim5+1<br>If BADL3A >=1 & BADL3A<=4 → funlim5=funlim5+1<br><br>**zfunlim5**<br>Z-score computed.	Variable name:<br>1. funlimP<br>2. funlim5<br>3. zfunlim5<br><br>Variable labels:<br>1. Number of functional limitations: proportionally scaled to number of items counted<br>2. Number of functional limitations (0-5)<br>3. Number of functional limitations (0-5) z-score – computed for each cohort individually<br><br>Value labels:<br>**FunlimP:**<br>Proportional scaling to account for difference in number of items counted across the cohorts:<br>funlimP= (funlim-MIN)/(MAX-MIN)*10

ActiFE Ulm	**How great difficulty do you have a staircase with 15 steps up and to go down without taking a break?** (ADL_stairs_BL)<br><br>**How much difficulty do you have to dress and undress?** (ADL_dressing_BL)<br><br>**How much difficulty do you have to cut your toenails?** (IADL_toenails_BL)<br><br>**How much trouble do you have to take a five-minute walk without stopping?** (ADL_walk_BL)<br><br>**How much difficulty do you have to sit on a chair and to get up?** (ADL_chair_BL)<br><br>How much difficulty do you have to take a shower or bath? (ADL_shower_BL)	0= None<br>1= Some difficulty (*light* difficulty)<br>2= moderate<br>3= large<br>4= not feasible, need help	**funlimP**<br>Calculate number of limitations by adding 1 point for each difficulty (some difficulty or more; 1-4)<br>If ADL_stairs_BL >= 1 → funlimp = funlimp +1<br>If ADL_dressing_BL >= 1 → funlimp = funlimp +1<br>If ADL_toenails_BL >= 1 → funlimp = funlimp +1<br>If ADL_walk_BL >= 1 → funlimp = funlimp +1<br>If ADL_chair_BL >= 1 → funlimp = funlimp +1<br>If ADL_shower_BL >= 1 → funlimp = funlimp +1<br><br>**Funlim5**<br>Calculate number of limitations by adding 1 point for each difficulty (some difficulty or more; 1-4)<br>If ADL_stairs_BL >= 1 → funlim5 = funlim5 +1<br>If ADL_dressing_BL >= 1 → funlim5 = funlim5 +1<br>If ADL_toenails_BL >= 1 → funlim5 = funlim5 +1<br>If ADL_walk_BL >= 1 → funlim5 = funlim5 +1<br>If ADL_chair_BL >= 1 → funlim5 = funlim5 +1<br><br>**zfunlim5**<br>Z-score computed.	**Funlim5:**<br>Range 0-5<br><br>Activities selected for funlim5 are:<br>1. climbing stairs<br>2. dressing<br>3. 'cutting toenails' or 'bending, kneeling, stooping'<br>4. 'walk outside for 5 min' or 'walking 100 meters/yards'<br>5. Get up from chair or toilet
TILDA	Because of a physical or mental health problem, do you have difficulty doing any of the activities on the card:<br>-**Walking 100 meters (100 yards) (fl001_01)**<br>-Running or jogging about 1.5 kilometers (1 mile) (fl001_02)<br>-Sitting for about two hours (fl001_03)<br>-Getting up from a chair after sitting for long periods (fl001_04)<br>-Climbing several flights of stairs without resting (fl001_05)<br>-**Climbing one flight of stairs without resting (fl001_06)**<br>-**Stooping, kneeling, or crouching(fl001_07)**<br>-Reaching or extending your arms above shoulder level (fl001_08)<br>-Pulling or pushing large objects like a living room chair (fl001_09)<br>-Lifting or carrying weights over 10 pounds/5 kilos, like a heavy bag of groceries (fl001_10)<br>Because of a health or memory problem, do you have difficulty doing any of the activities on this card?<br>-Picking up a small coin from a table (fl001_11)<br>-**Dressing, including putting on shoes and socks (fl002_1)**<br>-Walking across a room (fl002_**2)**<br>-Bathing or showering (fl002_3)<br>-Eating, such as cutting up your food (fl002_4)<br>-Getting in or out of bed (fl002_5)<br>-**Using the toilet, including getting up or down** (fl002_6)	0=no<br>1=yes	**funlimP**<br>Calculate number of limitations by adding 1 point for each 'yes' to the following 10 items:<br>funlimP=fl001_01+fl001_02+fl001_03+fl001_04+fl001_05+fl001_06+fl001_07+fl001_08+fl001_09+fl001_10<br><br>**funlim5**:<br>funlim5=fl001_06 + fl001_01 + fl001_07 + fl002_6 +fl002_1<br><br>**zfunlim5**<br>Z-score computed.	

B-PROOF	SF12 physical function subscale:<br>- Does your health limit you in moderate activities (moving a table, playing golf, etc.)? (SF12_2)<br>- Does your health limit you in climbing stairs? (SF12_3)<br>- During the past 4 weeks, have you accomplished less than you would like as a result of your physical health? (SF12_4)<br>- During the past 4 weeks, were you limited in the kind of work or other regular activities you do as a result of your physical health? (SF12_5)<br><br>- During the past four weeks, how much did pain interfere with your normal work, including both work outside the home and housework? (SF12_8)<br><br>- During the past 4 weeks, how much of the time has your physical health or emotional problems interfered with your social activities like visiting with friends, relatives etc? (SF12_12)<br><br>Items from Euroqol:<br>- Mobility (Euroqol_1)<br><br>- Self-care (Euroqol_2)<br><br>- Daily activities (Euroqol_3)	Range<br>1 = limited a lot<br>2 = limited a little<br>3 = not limited at all<br><br>0 = no<br>1 = yes<br><br>1 = not at all<br>2 = slightly<br>3 = moderately<br>4 = quite a bit<br>5 = extremely<br><br>1 = all of the time<br>2 = most of the time<br>3 = some of the time<br>4 = a little of the time<br>5 = none of the time<br><br>Range 1-3:<br>1 = no problem with walking<br>2 = some problems with walking<br>3 = stay in bed most of the time<br><br>1 = no problem with washing or getting dressed<br>2 = some problems with washing or getting dressed<br>3 = not able to wash myself or get dressed<br><br>1 = no problems with daily activities<br>2 = some problems with daily activities<br>3 = not able to perform daily activities	Includes questions on climbing stairs, walking and self-care, but questions are asked differently, and there are no sufficient similar items to harmonize.	

Rotterdam Study	Questions e5_EI1_03 to e5_EI1_26 are based on the Stanford Health Assessment Questionnaire. Questions e5_EI1_28 to e5_EI1_33 are based on the Instrumental Activities of Daily Living scale:<br><br>Are you able to get your clothes from closets or drawers, on your own? (e5_EI1_03)<br>**Are you able to dress yourself, including doing the buttons, zippers, tying laces, etc**. (e5_EI1_04)<br>Are you able to wash your hair on your own? (e5_EI1_05)<br>Are you able to get up from a chair without using your arms as support? (e5_EI1_06)<br>Are you able to get out of your bed on your own? (e5_EI1_07)<br>Do you have trouble eating ? Do you have trouble cutting meat or bread, or do you have problems with drinking a full glass of milk? (e5_EI1_08)<br>Are you able to open a new carton of milk on your own? (e5_EI1_09)<br>**Are you able to walk outside on a flat terrain on your own?** (e5_EI1_10)<br>**Are you able to walk 5 steps on the stairs and back on your own**? (e5_EI1_11)<br>Are you able to wash and dry your entire body on your own? (e5_EI1_12)<br>Are you able to take a bath or use the shower on your own? (e5_EI1_13)<br>Are you able to open and close the tap of a sink on your own? (e5_EI1_14)<br>**Are you able to sit down on and get up from the toilet on your own?** (e5_EI1_15)<br>Are you able to comb / style your hair on your own? (e5_EI1_16)<br>Are you able to grab a pack of sugar (1 kg) from a shelf above your head? (e5_EI1_17)<br>**Are you able to bend, for example to grab clothes from the floor**? (e5_EI1_18)<br>Are you able to open a car door from the outside on your own? (e5_EI1_19)<br>Are you able to open a jar of jam, that has already been opened before? (e5_EI1_20)<br>Are you able to use a pen or pencil? (e5_EI1_21)<br>Are you able to do your daily grocery shopping? (e5_EI1_22)<br>Are you able to get in and out of a passenger car? (e5_EI1_23)<br>Are you able to travel independently? (e5_EI1_24)<br>Are you able to do small chores in the house? (e5_EI1_25)<br>Are you able to run the household on your own? (e5_EI1_26)<br>Are you able to cycle? (e5_EI1_27)<br>Do you have trouble using the telephone (at home or in a cell)? (e5_EI1_28)<br>Do you have trouble cooking a dish? (e5_EI1_29)<br>Do you have trouble doing the laundry? (e5_EI1_31)<br>Imagine you would have to use medication, are you able to take care of this yourself? (e5_EI1_32)<br>Do you have trouble arranging your finance? (e5_EI1_33)	1 = without difficulty<br>2 = with some difficulty<br>3 = with a lot of difficulty<br>4 = cannot do it her/himself (support needed)<br>7 = don't know<br>8 = N.a.<br>9 = No answer	**funlimP**<br>if funlim=e5_EI1_03+....+e5_EI1_33.<br><br>**Funlim5**<br>If e5_EI1_11>=2 and e5_EI1_11<=4 → funlim5=funlim5+1<br>If e5_EI1_18>=2 and e5_EI1_18<=4 → funlim5=funlim5+1<br>If e5_EI1_10>=2 and e5_EI1_10<=4 → funlim5=funlim5+1<br>If e5_EI1_15>=2 and e5_EI1_15<=4 → funlim5=funlim5+1<br>If e5_EI1_04>=2 and e5_EI1_04<=4→ funlim5=funlim5+1<br><br>**zfunlim5**<br>Z-score computed.	

Genetic variants				
LASA	See Appendix 7			See Appendix 7
ActiFE Ulm	No data			
TILDA	No data			
B-PROOF	See Appendix 7			
Rotterdam study	See Appendix 7			
Grip strength				
LASA	Grip strength dynamometer (wave C: Takei TKK 5001, Takei Scientific Instruments Co. Ltd., Tokyo, Japan. Wave 3B: JAMAR 5030J1 Hydraulic Hand Dynamometer)<br>Two attempts with each hand:<br>-left hand (BMED618, BMED619; CMED618, CMED619)<br>-right hand (BMED616, BMED617; CMED616, CMED617)		**grip, wave C:**<br>grip=max(CMED618, CMED619, CMED616, CMED617)<br><br>**grip, wave 3B:**<br>grip=max(BMED618, BMED619, BMED616, BMED617)<br><br>**zgrip**<br>Z-scores created.	Variable name:<br>grip<br>zgrip<br><br>Variable label:<br>Grip strength (kg) – maximum<br>Z-score of grip strength – computed for each cohort individually<br><br>Range 0-79<br><br>To my knowledge, there are no papers describing the inter-instrument reliability between the various instruments that were used in the cohorts. Therefore, z-scores were computed (zgrip) using the grip scores of each cohort individually.
ActiFE Ulm	A JAMAR dynamometer was used to measure grip strength.<br>Grip strength (kg); highest value of means of two measures (gripstrength_BL)<br>Grip strength right hand first try (kg) (gripstrength_r1_BL)<br>Grip strength left hand first try (kg) (gripstrength_l1_BL)<br>Grip strength right hand second try (kg) (gripstrength_r2_BL)<br>Grip strength left hand second try (kg) (gripstrength_l2_BL)	Range 6 – 110 kg	**grip**<br>grip = max (gripstrength_r1_BL, gripstrength_l1_BL, gripstrength_r2_BL, gripstrength_l2_BL)<br><br>**zgrip**<br>Z-scores created.	
TILDA	Grip strength was measured using a Baseline hydraulic hand dynamometer.<br>Two attempts with each hand:<br>-dominant hand (GRIPtest1D, GRIPtest2D)<br>-non-dominant hand (GRIPtest1ND, GRIPtest2ND)	Range 0-68 kg<br>Range 0-81 kg	**grip**<br>grip=max(GRIPtest1D, GRIPtest2D, GRIPtest1ND, GRIPtest2ND)<br><br>**zgrip**<br>Z-scores created.	

B-PROOF	Measured using a strain-gauged dynamometer (Takei, TKK 5401, Takei Scientific Instruments Co. Ltd., Japan, inter observer CV = 5%). Participants were asked to perform two maximum hand grip trials with each hand in standing position with their arms along their body. Maximal hand grip strength was defined as the average of the highest score of the left and right hand:<br>Dominant hand (hgs_dominant_hand)<br><br>Hand grip right hand (hgs_right_1st_attempt, hgs_right_2nd_attempt)<br>Hand grip left hand (hgs_left_1st_attempt, hgs_left_2nd_attempt)<br><br>Hand grip: position<br><br>Handgrip: remarks (grip_remarks)	1 = right<br>2 = left<br><br>In kg<br><br>No unit/categories included<br><br>1 = handicap right hand<br>2 = handicap left hand<br>3 = pain<br>Missing = no remark	**grip**<br>grip=max(hgs_right_1st_attempt, hgs_right_2nd_attempt, hgs_left_1st_attempt, hgs_left_2nd_attempt)<br><br>**zgrip**<br>Z-scores created.	
Rotterdam Study	Measurement of hand grip strength (in kg) (1) (e5_15813)<br>Measurement of hand grip strength (in kg) (2) (e5_15814)<br>Measurement of hand grip strength (in kg) (3) (e5_15815)<br><br>Which hand is measured? (e5_15816)<br><br>Grip strength was measured using a Fabrication Enterprises hydraulic hand dynamometer.	= 1 - 90<br>99 = missing<br>88 = not applicable/default<br><br>1 = right<br>2 = left<br>9 = missing<br>8 = not applicable/default	**grip**<br>If e5_15813<88 and e5_15814<88 and e5_15815<88 → grip=max(e5_15813, e5_15814, e5_15815)<br><br>If e5_15813=missing and e5_15814<88 and e5_15815<88 → grip=max(e5_15814, e5_15815)<br>If e5_15813<88 and e5_15814=missing and e5_15815<88 → grip=max(e5_15813, e5_15815)<br>If e5_15813<88 and e5_15814<88 and e5_15815=missing → grip=max(e5_15813, e5_15814)<br><br>If e5_15813=missing and e5_15814= missing and e5_15815<88 → grip=e5_15815<br>If e5_15813<88 and e5_15814=missing and e5_15815=missing → grip=e5_15813<br>If e5_15813= missing and e5_15814<88 and e5_15815= missing → grip=e5_15814<br><br>**zgrip**<br>Z-scores created.	
Hearing				
LASA	R wears hearing aid(s) (HA) how many hours (bsense7b)<br><br>Can you hear well enough? (bsense08, csense08)<br>**Can you follow a conversation in a group of three or four persons without a hearing aid?** (bsense09, csense09)<br>Can you follow a conversation in a group of three or four persons with a hearing aid? (bsense10, csense10)<br>**Can you follow a conversation with one person?** (bsense11, csense11)	-2=no hearing aid<br>-1=missing<br>1= less than 1 hr a day<br>2= 1 to 4 hrs a day<br>3= 4 to 8 hrs a day<br>4= all day<br><br>1=Yes, without difficulty<br>2=Yes, but with some difficulty<br>3=Yes, but with much difficulty<br>4=No I cannot	**hearing, wave C**<br>If (csense09=1 or csense09=2) & csense11=1 → hearing=0<br>If csense09>=3 or csense11>=2 → hearing=1<br><br>**hearing, wave 3B**<br>If (bsense09=1 or bsense09=2) & bsense11=1 → hearing=0<br>If bsense09>=3 or bsense11>=2 → hearing=1<br><br>**hearingaid (only for 3B)**<br>if bsense7b=-2 → hearingaid =0<br>if bsense7b>=1 → hearingaid=1	Variable name:<br>hearing<br>hearingaid<br><br>Variable labels:<br>Do you have hearing problems (subjective)?<br>Do you (sometimes) use a hearing aid?<br><br>Value labels<br>0=no<br>1=yes<br><br>Note: The use of a hearing aid is not considered in the hearing variable

	Can you follow a conversation with one person with a hearing aid? (bsense12, csense12)	Additional response option for (sense10 and sense12):<br>5=Respondent does not have a hearing aid		
ActiFE Ulm	hearing (hear_BL)<br>Measured by interviewers' impression.	0=good<br>1=bad	**Hearing**<br>Use as is.<br>hearing=hear_BL	
TILDA	Do you have any of the following aids or appliances to help with your hearing:<br>-hearing aid (all the time) (ph107_1)<br>-hearing aid (some of the time) (ph107_2)<br>- amplifier (ph107_3) is not available in public data<br>-none of the above (ph107_4)<br><br>**Is your hearing with or without a hearing aid … (ph108)**<br><br>**Not in public data wave 1:**<br>Can you follow a conversation with one person (with or without a hearing aid)? (ph109)<br>Can you follow a conversation with four people (with or without a hearing aid)? (ph110)	0=no<br>1=yes<br><br>1=excellent<br>2=very good<br>3=good<br>4=fair<br>5=poor<br><br>1=with no difficulty<br>2=with some difficulty<br>3=with much difficulty<br>4=no I cannot	**hearing**<br>If ph108=1 or ph108=2 or ph108=3 → hearing=0<br>If ph108=4 or ph108=5 → hearing=1<br><br>**hearingaid**<br>if ph107_1=0 and ph107_2 = 0 → hearingaid =0<br>if ph107_1=1 or ph107_2=1 → hearingaid = 1	
B-PROOF	No data available		Not harmonized.	

Rotterdam Study	**Do you think you hear less (without any hearing aids)?** (e5_EIKNO1)<br><br>Do you use any hearing aids? (e5_EIKNO2)<br><br>Do you think you hear less, even when using your hearing aids? (e5_EIKNO2A)<br><br>Are you able to have a conversation with more than 3 persons? (e5_EIKNO3)<br>Do you avoid certain occasions (e.g. birthdays) because of your hearing? (e5_EIKNO4)<br><br>Do you ever hear sounds in your head or (one of) your ears, such as buzzing, peeping, humming, while there's no clear noise source closeby? (e5_EIKNO5)<br><br>If yes, do these sounds hinder your daily activities? (e5_EIKNO5A)	0 = no, I hear almost everything<br>1 = yes, sometimes I don't hear what people are saying<br>2 = yes, I often don't hear what people are saying<br>3 = yes, I (almost) never hear what people are saying<br>7 = don't know<br>9 = no answer<br><br>0 = no<br>1 = yes, hearing instrument(s)<br>2 = yes, a BAHA<br>3 = ja, een cochlear implant<br>7 = don't know<br>9 = no answer<br><br>0 = no, I hear almost everything<br>1 = yes, sometimes I don't hear what people are saying<br>2 = yes, I often don't hear what people are saying<br>3 = yes, I (almost) never hear what people are saying<br>7 = don't know<br>8 = n.a.<br>9 = no answer<br><br>0 = (almost) never<br>1 = sometimes<br>2 = often<br>3 = almost always<br>7 = don't know<br>9 = no answer<br><br>0 = no<br>1 = yes, < 1 times a week<br>2 = yes, >= 1 times a week, but not daily<br>3 = yes, everyday<br>7 = don't know<br>9 = no answer<br><br>0 = no<br>1 = yes<br>7 = don't know<br>8 = n.a.<br>9 = no answer	**hearing**<br>If e5_EIKNO1 = 2 or e5_EIKNO1 = 3 → hearing=1<br>If e5_EIKNO1 = 0 or e5_EIKNO1 = 1 → hearing=0<br><br>**hearingaid**<br>If e5_EIKNO2 = 0 → hearingaid = 0<br>If e5_EIKNO2>=1 and e5_EIKNO2<=3 → hearingaid = 1	

**Heart disease**				
LASA	Do you have a heart disease? (BHART01, CHART01)	3B:<br>1=no<br>2=yes<br><br>C:<br>0=no, never<br>1=no, but at wave B yes<br>2=yes, but at wave B no<br>3=yes, and at wave B yes	**Wave C:**<br>If chart01=0 → heart=0<br>If chart01=1 or chart01=2 or chart01=3 → heart=1<br><br>**Wave 3B:**<br>If BHART01=2 → heart=1<br>If BHART01=1 → heart=0	The harmonization algorithms use different conditions for determining history of heart disease. Therefore, individual cardiovascular conditions should be used instead of this harmonized variable.<br><br>Variable name:<br>heart<br><br>Variable label:<br>History of heart disease<br><br>Value labels:<br>0=no<br>1=yes
ActiFE Ulm	(C_myocardinf_BL) Myocardial infarction<br>(C_heartf_BL) Heart failure	0=no<br>1=yes	If C_myocardinf_BL = 0 AND C_heartf_BL = 0 → heart = 0<br>If C_myocardinf_BL = 1 OR C_heartf_BL = 1 → heart = 1	
TILDA	Has a doctor ever told you that you have any of the following conditions:<br>-angina (ph201_02)<br>-heart attack (ph201_03)<br>-heart failure (ph201_04)<br>-heart murmur (ph201_09)<br>-abnormal heart rhythm (ph201_10)<br>-other heart trouble (ph201_11)	0=no<br>1=yes	If ph201_02=1 | ph201_03=1 | ph201_04=1 | ph201_09=1 | ph201_10=1 | ph201_11=1 → heart=1<br><br>If ph201_02=0 & ph201_03=0 & ph201_04=0 & ph201_09=0 & ph201_10=0 & ph201_11=0 → heart=0	
B-PROOF	Do/did you have heart problems? (heart_problem)<br><br>What heart problems do/did you have? (heart_problem_what)<br><br>Which type of CVD does participant have? (CVdisease)	0 = no<br>1 = yes<br><br>String<br><br>1 = arrhythmia<br>2 = angina pectoris<br>3 = myocardial infarction<br>4 = heart failure<br>5 = atrial septum defect<br>6 = pericarditis<br>7 = aneurysm<br>8 = pulmonal hypertension	Heart=heart problem	
Rotterdam Study	After (date interview ERGO-4/ErgoPlus-2/ErgoJong-1), have you ever been treated in the hospital for a narrowing of the blood vessels of the heart? (e5_EIPTCA)<br><br>After (date interview ERGO-4/ErgoPlus-2/ErgoJong-1), did you experience a heart attack? (e5_EIMI)	0 = No<br>1 = Yes<br>7 = Don't know<br>9 = No answer<br><br>0 = no<br>1 = yes<br>7 = don't know<br>9 = no answer	Not harmonized; this is too specific for Heart disease.	
**Height**				
LASA	Measured height in centimetres (BMED150, CMED150)		height=CMED150/BMED150	Variable name:<br>height<br><br>Variable label:<br>Height in cm
ActiFE Ulm	body height (m) (height_BL)		height = height_BL x 100	
TILDA	Measured height in centimetres (height)	Height: 145-185 cm<br><br>(NB: 145= '<=145'; 185= '185+')	height=height	

B-PROOF	Measured height (Length)	In cm, 1 decimal	height=Length	
Rotterdam Study	Height, standing (in cm) (e5_229)	= 100.0 - 250.0<br>999.9 = missing<br>888.8 = not appropriate-default	height=e5_229	
**Hypertension**				
LASA	Self-reported chronic conditions:<br>-hypertension (BHBD1, CHBD1)<br><br>Follow-up questions only asked to those responding 'yes' to first question:<br>-From which age have you had hypertension? (BHBD2, CHBD2)<br><br>-Are you taking medication for hypertension? (BHBD2A, CHBD2A)<br><br>-Are you being treated for hypertension by a doctor? (BHBD3, CHBD3)	1=no<br>2=yes<br><br><br><br>Range 0-64<br><br>1= no<br>2=yes<br><br>1=no<br>2=yes, family physician<br>3=yes, specialist	**hypertension:**<br>If B/CHBD1=2 → hypertension=1<br>If B/CHBD1=1 → hypertension=0<br><br>**hypertensionm:**<br>if not missing (BPsys) and not missing(BPdias) → hypertensionm = 0*<br>if BPsys >= 140 or BPdias >= 90 → hypertensionm = 1<br><br>*) See blood pressure.<br><br>**hypmsr**<br>if hypertension = 0 or hypertensionm = 0 → hypmsr = 0<br>if hypertension = 1 or hypertensionm = 1 → hypmsr = 1<br><br>**hypmed:**<br>If B/CHBD2A=1 → hypmed=0<br>If B/CHBD2A=2 → hypmed=1	Variable name:<br>1. hypertension<br>2. hypertensionm<br>3. hypmsr<br>4. hypmed<br><br>Variable label:<br>1. Does the respondent have hypertension? (self-reported)<br>2. Does the respondent have hypertension? (measured; based on lowest BP measurements)<br>3. Does the respondent have hypertension? (measured (based on lowest BP measurements) OR self-reported)<br>4. Taking hypertension medication?<br><br>Value label:<br>0=no<br>1=yes<br><br>Notes:<br>According to most national guidelines, the threshold for the diagnosis of hypertension is a systolic blood pressure of at least 140 mm Hg, a diastolic blood pressure of at least 90 mm Hg, or both (https://www.sciencedirect.com/science/article/pii/S0140673614614689?via%3Dihub).<br>The lowest measured value was used for determining presence of hypertension. This will likely overestimate the prevalence of hypertension in LASA C, as BP was only measured once in this cohort (1509 valid cases). We chose to use the lowest BP measurements as opposed to the first BP measurement for determining hypertension, since the very first BP measurements typically are overestimations. Also note that in B-Proof, there are 1243 respondents for whom only the lowest measure was reported.<br><br>Note that for hypmed it may be more appropriate to use the dichotomous variables for cardiovascular drugs related to treatment of hypertension.
ActiFE Ulm	hypertension until baseline (C_hypert_BL)	0=no<br>1=yes	**hypertension:**<br>Use as is.<br>hypertension = **C_hypert_BL**<br><br>**hypertensionm:**<br>if not missing (BPsys) and not missisng(BPdias) → hypertensionm = 0*<br>if BPsys >= 140 or BPdias >= 90 → hypertensionm = 1<br><br>*) See blood pressure.<br><br>**hypmsr**<br>if hypertension = 0 or hypertensionm = 0 → hypmsr = 0<br>if hypertension = 1 or hypertensionm = 1 → hypmsr = 1<br><br>**hypmed**<br>Not harmonized.	
TILDA	Has a doctor ever told you that you have any of the following conditions: high blood pressure (ph201_01)<br><br>Are you currently taking any tablets or pills for high blood pressure? (ph202a)	0=no<br>1=yes<br><br>-1=not asked<br>1=yes<br>5=no	**hypertension:**<br>Use as is.<br>ph201_01=hypertension<br><br>**hypertensionm:**<br>if not missing (BPsys) and not missing(BPdias) → hypertensionm = 0*<br>if BPsys >= 140 or BPdias >= 90 → hypertensionm = 1<br><br>*) See blood pressure.<br><br>**hypmsr**<br>if hypertension = 0 or hypertensionm = 0 → hypmsr = 0<br>if hypertension = 1 or hypertensionm = 1 → hypmsr = 1<br><br>**hypmed:**<br>if ph202a=1 → hypmed=1	

			if ph202a=5 → hypmed=0	
B-PROOF	Does the participant have elevated blood pressure? (BloodPressure)<br><br>Hypertensive medication from the medication data? (which included)	0 = no<br>1 = yes	**hypertension**<br>Use as is.<br>Hypertension=BloodPressure<br><br>**hypertensionm:**<br>if not missing (BPsys) and not missing(BPdias) → hypertensionm = 0*<br>if BPsys >= 140 or BPdias >= 90 → hypertensionm = 1<br><br>*) See blood pressure.<br><br>**hypmsr**<br>if hypertension = 0 or hypertensionm = 0 → hypmsr = 0<br>if hypertension = 1 or hypertensionm = 1 → hypmsr = 1<br><br>**hypmed:**<br>Not harmonized.	
Rotterdam Study	Are you taking any medication for high blood pressure at the moment? (e5_EIMC8C)	0 = no<br>1 = yes<br>7 = don't know<br>8 = n.a.<br>9 = no answer	**hypertension**<br>Not harmonized.<br><br>**hypertensionm:**<br>if not missing (BPsys) and not missing(BPdias) → hypertensionm = 0*<br>if BPsys >= 140 or BPdias >= 90 → hypertensionm = 1<br><br>*) See blood pressure.<br><br>**hypmsr**<br>if hypertensionm = 0 → hypmsr = 0<br>if hypertensionm = 1 → hypmsr = 1<br><br>**hypmed:**<br>If e5_EIMC8C=1 → hypmed=1<br>If e5_EIMC8C=0 → hypmed=0	
ID number				
LASA	Respondent number (respnr)	Range 71000-94138	**Wave C:**<br>ID=respnr + 100000000<br><br>**Wave 3B:**<br>ID=respnr + 200000000	Variable name:<br>ID<br><br>Unit:<br>ID number
ActiFE Ulm	study ID (sid)	Range 20034-79997	ID= sid + 300000000	

TILDA	TILDA unique identifier (id)<br><br>A unique identifier for each participant is provided (id), along with identifiers for the household (household) and geographic cluster (cluster) to which they belong. As this variable contains non-numerical information, a new ID variable was constructed.	Range 01021-40942	id was first reconstructed by assigning a random number to each respondent.<br>Then:<br>ID=id + 400000000	
B-PROOF	Participant number (participant_ID)	Range 5008-32343	ID= participant_ID + 500000000	
Rotterdam Study	Identification (ergoid)	Range 1-9604001	ergoid was first reconstructed by assigning a random number to each respondent.<br>Then:<br>ID= ergoid + 600000000	
Income				
LASA	Income of respondent + partner per month (binccat, cinccat)	Wave C:<br>0=less than 999 guilder (< 453 euro)<br>1=1000-1250 guilder (454 - 567)<br>2=1251-1500 guilder (567 - 680)<br>3=1501-1750 guilder (681 - 794)<br>4=1751-2000 guilder (795 - 907)<br>5=2001-2250 guilder (908 - 1021)<br>6=2251-2500 guilder (1022 - 1134)<br>7=2501-3000 guilder (1135 - 1361)<br>8=3001-3500 guilder (1362 - 1588)<br>9=3501-4000 guilder (1589 - 1815)<br>10=4001-4500 guilder (1816 - 2042)<br>11=4501-5000 guilder (2043 - 2269)<br>12=5001 or more (2270 +)<br><br>Wave 3B:<br>1=454-567 euro<br>2=568-680 euro<br>3=681-794 euro<br>4=795-907 euro<br>5=908-1021 euro<br>6=1022-1134 euro<br>7=1135-1361 euro<br>8=1362-1588 euro<br>9=1589-1815 euro<br>10=1816-2042 euro<br>11=2043-2269 euro<br>12=2270-2495 euro<br>13=2496-2722 euro<br>14=2723-2949 euro<br>15=2950-3176 euro<br>16=3177-3403 euro<br>17=3404-3630 euro<br>18=3631-3857 euro<br>19=3858-4084 euro<br>20=4085-4311 euro	**Wave 3B:**<br>If binccat>=0 and <=3 → income=1<br>If binccat>=4 & binccat<=8 → income=2<br>If binccat>=9 → income=3<br><br>**Wave C:**<br>If cinccat>=0 and <=3 → income=1<br>If cinccat>=4 & cinccat<=8 → income=2<br>If cinccat>=9 → income=3<br><br>Note: if resp. had missing data on c/binccat and reported having no income on the follow-up question:<br>If c/binccat<0 & c/bincf=0 → income=1	Variable name:<br>income<br><br>Variable label:<br>Annual household income (euro)<br><br>Value labels:<br>1= <10,000<br>2= 10,000 - 20,000<br>3= >20,000<br><br>Note: the cut-off values were based on TILDA's value labels.

		21=4312-4537 euro<br>22=4538-4991 euro<br>23=4992-5445 euro<br>24=5446 or more		
ActiFE Ulm	No data available.		Not harmonized.	
TILDA	How much income in total have these people received during the last 12 months? (si408)<br><br>Did the household income amount to a total of less than XXXXX, more than YYYYY? (si409)	Range 0-2,000,000<br><br><br>1= < 10,000 euro<br>2= 10,000 - 20,000 euro<br>3= 20,000 - 40,000 euro<br>4= 40,000 - 70,000 euro<br>5= >70,000 euro	If si408 >=0 and si408 <10,000 → income=1<br>If si408 >=10,000 & si408<20,000 → income=2<br>If si408 >20,000 → income=3<br><br>If si408 = missing & si409=1 → income=1<br>If si408 = missing & si409=2 → income=2<br>If si408 = missing & (si409=3 | si408 = 4 | si408 = 5) → income=3	
B-PROOF	No data available		Not harmonized.	
Rotterdam Study	No data available for ERGO-5; only data on baseline.		Not harmonized.	
Injurious falls				
LASA	No data available on injurious falls.		Not harmonized.	Variable name:<br>ifalls<br><br>Variable label:<br>In the past 12 months, have you had an injurious fall requiring medical attention?<br><br>Value label:<br>0=no<br>1=yes
ActiFE Ulm	frequency of injurious falls within the observation period following baseline [fall calendar] (FC_injury_fall_BL)<br><br>Note that these are prospective falls.	Range 0 - 24	If FC_fall_BL = 0 → ifalls = 0<br>If FC_injury _fall_BL = 0 → ifalls = 0<br>If FC_injury_fall_BL > 0 → ifalls = 1	
TILDA	Did you injure yourself seriously enough to need medical treatment? (ph404)	1=yes, and I got treatment<br>2=yes and I did not get treatment<br>5=no	If ph404=1 | ph404=2 → ifalls=1<br>If ph404=5 | ph401=5 → ifalls=0	
B-PROOF	No data available, but data on fractures after falls in period before baseline:<br>Did participant ever sustain fracture? (fractures)<br><br><br>When did the last fracture occur? (last_fracture_when)<br><br>Cause of the most recent fracture? (cause_fracture)<br><br><br><br><br>Did participant sustain another fracture? (fractures_2)<br><br><br>When did last-but-one fracture occur? (last_but_one_fracture_when)<br><br>Cause of last-but-one fracture? (cause_last_but_one_fracture)	 <br>0=no<br>1=yes<br><br>Date<br><br>1=fall > 'standing height'<br>2=fall from 'standing height'<br>3='traffic incident'<br>4='other'<br><br>0=no<br>1=yes<br><br>Date<br><br><br>1=fall > 'standing height'<br>2=fall from 'standing height'<br>3='traffic incident'<br>4='other'	Should only cover injurious falls in past 12 months, so:<br><br>If cause_fracture = 1 or cause_fracture = 2 or cause_last_but_one_fracture =1 or cause_last_but_one_fracture = 2 → ifalls=1<br>If cause_fracture = 3 or cause_fracture = 4 or cause_last_but_one_fracture = 3 or cause_last_but_one_fracture = 4 → ifalls=0<br><br>year_from_date=XDATE.year(date_interview).<br><br>diffdate_frac=year_from_date-last_fracture_when	

Rotterdam Study	Did this/these fall(s) in the past 12 months have any serious consequences such as a broken bone? (e5_EIMC27A)	0 = no<br>1 = Yes, have broken one of more bones<br>2 = yes, head trauma<br>3 = yes, other<br>7 = don't know<br>8 = n.a.<br>9 = no answer	If e5_EIMC27A=0 or e5_EIMC27A=8 → ifalls=0<br>If e5_EIMC27A>=1 and e5_EIMC27A<=3 → ifalls=1	
Living situation				
LASA	C & 3B: type of housing (chindep/bhindep)<br><br>C & 3B: type of housing (choustyp/bhoustyp)	-1= asked no answer<br>1= independent<br>2= residential home<br>3= nursing home-somatic<br>4= nursing home-psychiatric<br>5= hospital<br>6= psychiatric hospital<br>8= monastery (added)<br><br>Coding in 3B and C differs. **3B:**<br>-3=Na, wrong skip<br>-2=Not applicable<br>-1=unknown<br>1=attached row or semi-detached<br>2=detached<br>3=high rise (elevator)<br>4=high rise (no elevator)<br>5=ground floor apartment<br>6=apt build elderly (elevator)<br>7=home elderly (street level)<br>8=semi-independent<br>9=farm/corporate housing<br>10=houseboat<br>11=housing with communal facilities<br>12=other<br>13=institution<br>14=monastery	If chindep/bhindep=1 → living=0<br>If chindep/bhindep=2 or chindep/bhindep=3 or chindep/bhindep=4 or chindep/bhindep=5 or chindep/bhindep=6 or chindep/bhindep=8 → living=1	Variable name:<br>living<br><br>Variable label:<br>Living situation<br><br>Value label:<br>0=community-dwelling<br>1=institutionalized<br><br>Same approach taken as in EPOSA harmonization guide. Note: enough cases institutionalized? Also: do we use type of house? (i.e. apartment, (semi-)detached, terraced etc.)
ActiFE Ulm	What kind of house do you live in? (IV3N877)<br><br>ActiFE Ulm only includes community-dwelling older persons.	1=one family house<br>2=two family house/row house<br>3=apartment in apartment building	living = 0	
TILDA	Only info on house type as all respondents are community-dwelling: Now I have a few questions about your place of residence. Is this (hw101)	1=a detached house<br>2=a semi-detached house<br>3=a terraced house<br>4=an apartment/ flat/bedsitter<br>95=other (specify)	living=0	

B-PROOF	No info on type of house only living situation (living_sit)	1=independent<br>2=assisted living<br>3=service flat<br>4=home for the elderly	If living_sit=1 → living=0<br>If living_sit=2 or living_sit=3 or living_sit=4 → living=1	
Rotterdam study	How is your living situation? (e5_EIHOME)	1=independent<br>2=service flat/supported housing/community<br>3=residential care home<br>4=nursing home<br>5=other<br>7=don't know<br>9=no answer	If e5_EIHOME=1 →living=0<br>If e5_EIHOME=2 or e5_EIHOME=3 or e5_EIHOME=4 → living=1	
Lung disease				
LASA	Do you have: asthma, chronic bronchitis or emphysema (BCARA01, CCARA01)	3B:<br>1=no<br>2=yes<br><br>C:<br>0=no, never<br>1=no, but at wave B yes<br>2=yes, but at wave B no<br>3=yes, and at wave B yes	**C:**<br>If ccara01=0 → lung=0<br>If ccara01=1 or ccara01=2 or ccara01=3 → lung=1<br><br>**3B:**<br>If BCARA01=1 → lung=0<br>If BCARA01=2 → lung=1	Variable name:<br>lung<br><br>Variable label:<br>Lung disease<br><br>Value label:<br>0=no<br>1=yes<br><br>The number of lung disease that are reported on differs between the cohorts. Also, the questions are asked differently; for example in LASA, presence of asthma, chronic bronchitis or emphysema is all assessed in the same yes/no question.
ActiFE Ulm	asthma until baseline (C_asthma_BL)<br><br>chronic obstructive lung disease until baseline (C_copd_BL)	0=no<br>1=yes	If C_asthma_BL =0 & C_copd_BL =0 → lung=0<br>If C_asthma_BL =1 | C_copd_BL =1 → lung=1	
TILDA	Has a doctor ever told you that you have any of the following conditions?<br>-Asthma (ph301_02)<br>-Chronic lung disease such as bronchitis or emphysema (ph301_01)	0=no<br>1=yes	If ph301_02=0 & ph301_01=0 → lung=0<br>If ph301_02=1 | ph301_01=1 → lung=1	
B-PROOF	No data available.		Not harmonized.	

Rotterdam Study	Have you ever been diagnosed with house dust mite allergy, hay fever or asthma (no COPD)? (e5_EIALLG)<br><br>In the last two years, have you been coughing almost daily for three months in a row? (e5_EICARA1)<br>In the last two years, have you been coughing up mucus almost daily for three months in a row? (e5_EICARA2)<br>Have you ever experience wheezing on the chest? (e5_EICARA4)<br>In the last two years, have you experienced this for longer than a week? (e5_EICARA5)<br>Have you ever experienced attacks of asthma? (e5_EICARA6)<br><br>Do you know whether you have any lung problems? (e5_18424)<br>-Asthma (e5_18425)<br>- COPD (chronic bronchitis / lung emphysema) (e5_18427)	0 = no<br>1 = yes<br>7 = don't know<br>9 = no answer<br><br>0 = no<br>1 = yes<br>7 = don't know<br>9 = no answer<br><br>0=nee<br>1=ja<br>8=not applicable/default<br>9=missing	If e5_18424=1 and (e5_18425=1 or e5_18427=1) → lung=1<br>If e5_18424=0 → lung=0	
Marital status				
LASA	Current marital status (bmarst, cmarst)	1=never married (*single)*<br>2=married (partner)<br>3=divorced (sep)<br>4=widowhood (widow)<br>5=registered partnership (partner)	**C & 3B:**<br>If cmarst/bmarst=2 → marital=1<br>If cmarst/bmarst=5 → marital=1<br>If cmarst/bmarst=1 → marital=2<br>If cmarst/bmarst=3 → marital=3<br>If cmarst/bmarst=4 → marital=4<br><br>**partner**<br>If marital >= 1 and marital <= 4 → partner = 0<br>If marital = 1 → partner = 1	Variable name:<br>marital<br>partner<br><br>Variable label:<br>Current marital status<br>Living with partner<br><br>Value labels:<br>1 = Partnered or (re)married<br>2= Living apart or single<br>3 = Separated or divorced<br>4 = widowed
ActiFE Ulm	Marital status (marital_BL)	1:married, (partner)<br>2:single, *(single)*<br>3:divorced (sep)<br>4:widowed (widow)<br>5:live apart (single)	**marital**<br>If marital_BL =1 → marital=1<br>If marital_BL =2 → marital=2<br>If marital_BL =3 → marital=3<br>If marital_BL =4 → marital=4<br>If marital_BL =5 → marital= 2<br><br>**partner**<br>If marital >= 1 and marital <= 4 → partner = 0<br>If marital = 1 → partner = 1	
TILDA	Are you ….? (cs006)	1=married (partner)<br>2=living with a partner as if married (partner)<br>3=single (never married) *(single)*<br>4=separated (sep)<br>5=divorced (sep)<br>6=widowed (widow)	**marital**<br>If cs006=1 → marital=1<br>If cs006=2 → marital=1<br>If cs006=3 → marital=2<br>If cs006=4 or cs006=5 → marital=3<br>If cs006=6 → marital=4<br><br>**partner**<br>If marital >= 1 and marital <= 4 → partner = 0<br>If marital = 1 → partner = 1	

B-PROOF	What is your marital status? (marital_status)	1 = unmarried (*single*)<br>2 = living together with a partner (partner)<br>3 = married (partner)<br>4 = widow/widower (widow)<br>5 = divorced (sep)	**marital**<br>If marital_status=3 → marital=1<br>If marital_status=2 → marital=1<br>If marital_status=1 → marital=2<br>If marital_status=5 → marital=3<br>If marital_status=4 → marital=4<br><br>**partner**<br>If marital >= 1 and marital <= 4 → partner = 0<br>If marital = 1 → partner = 1	
Rotterdam Study	What is your marital status? (e5_EICIVIL)<br><br>Note: no registered partnership category.<br>Note: widower & then married: falls under the married category now.	1 = Never been married (*single*)<br>2 = Married/ living together with a partner (also: divorced and remarried) (partner)<br>3 = widow/widower(widow)<br>4 = Divorced (sep)<br>5 = widow/widower and remarried (partner)<br>9 = No answer	**marital**<br>If e5_EICIVIL=2 or e5_EICIVIL=5 → marital=1<br>If e5_EICIVIL=1 → marital=2<br>If e5_EICIVIL=4 → marital=3<br>If e5_EICIVIL=3 → marital=4<br><br>**partner**<br>If marital >= 1 and marital <= 4 → partner = 0<br>If marital = 1 → partner = 1	
Memory				
LASA	Word list recall (15 words)<br>-trail 1 (BMWTT1, CMWTT1)<br>-trail 2 (BMWTT2, CMWTT2)<br>-trail 3 (BMWTT3, CMWTT3)<br><br>Delayed recall (BMWTDR, CMWTDR)	 Range 0-15<br>Range 0-15<br>Range 0-15<br><br>Range 0-15	irecall= BMWTT1+ BMWTT2<br>if BMWTT1>10 → irecall=10+BMWTT2<br>if BMWTT2>10 → irecall=BMWTT1+10<br>if BMWTT1>10 & BMWTT2>10 → irecall=20<br><br>drecall=BMWTDR<br>if drecall>10 → drecall=10	Variable name:<br>1. irecall<br>2. drecall<br><br>Variable labels:<br>1. immediate recall (number of words)<br>2. delayed recall (number of words)<br><br>Values:<br>1. Range 0-20<br>2. Range 0-10<br><br>Note: For consistency across the studies:<br>-the maximum number of words to recall per trail was capped at 10<br>-only the first 2 trails were used for irecall
ActiFE Ulm	No data available.		Not harmonized.	
TILDA	Word list recall (10 words)<br>-trail 1 (COGimmediaterecall1)<br>-trail 2 (COGimmediaterecall2)<br><br>Delayed recall (COGdelayedrecall)	 Range 0-10<br>Range 0-10<br><br>Range 0-10	irecall= COGimmediaterecall1+ COGimmediaterecall2<br><br>drecall=COGdelayedrecall	
B-PROOF	Rey Auditory Verbal Learning Test (RAVLT):<br>- 1st recall (RAVLT_1_corr, RAVLT_1_incorr, RAVLT_1_double)<br>- 2nd recall (RAVLT_2_corr, RAVLT_2_incorr, RAVLT_2_double)<br>- 3rd recall (RAVLT_3_corr, RAVLT_3_incorr, RAVLT_3_double)<br>- 4th recall (RAVLT_4_corr, RAVLT_4_incorr, RAVLT_4_double)<br>- 5th recall (RAVLT_5_corr, RAVLT_5_incorr, RAVLT_5_double)<br>- Delayed recall (RAVLT_DR_corr, RAVLT_dr_incorr, RAVLT_dr_double)<br>- Delayed recognition (RAVLT_del_recogn_score)	Score, max 15<br><br>Score, max 15<br><br>Score, max 15<br><br>Score, max 15<br><br>Score, max 15<br><br>Score, max 15<br><br>Score, max 30	Irecall= RAVLT_1_corr+ RAVLT_2_corr<br>if RAVLT_1_corr >10 → irecall=10+ RAVLT_2_corr<br>if RAVLT_2_corr >10 → irecall= RAVLT_1_corr +10<br>if RAVLT_1_corr >10 & RAVLT_2_corr >10 → irecall=20<br><br>drecall= RAVLT_DR_corr<br>if drecall>10 → drecall=10	

Rotterdam Study	Words Learning Test-15 test<br>Correct answers 15 WLT trial 1, 2, 3 (e5_2697, e5_2698, e5_2699)<br>Correct answers 15 WLT delayed recall (e5_2700)<br>Wrong words 15 WLT trial 1, 2, 3 (e5_15752, e5_15753, e5_15754)<br>Wrong words 15 WLT delayed recall (e5_15755)<br>Double words 15 WLT trial 1, 2, 3 (e5_15756, e5_15757, e5_15758)<br>Double words 15 WLT delayed recall (e5_15759)<br>Double wrong words 15 WLT trial 1, 2, 3 (e5_15760, e5_15761, e5_15762)<br>Double wrong words 15 WLT delayed recall (e5_15763)<br><br>Test status 15 Words Learning Test (e5_13188)<br><br><br><br><br><br><br><br><br><br><br>Correct positive answers 15 WLT recognition task (13191)<br>Incorrect positive answers 15 WLT recognition task (13192)<br>Incorrect negative answers 15 WLT recognition task (2748)<br><br>Test status 15 WLT recognition task (13189)	= 0 - 15<br>99 = missing<br>88 = not applicable/default<br><br><br><br><br><br><br><br><br><br><br><br>1 = complete and reliable<br>2 = technical problems<br>3 = refusal<br>4 = cognitive limitations<br>5 = physical limitations<br>6 = deviation fr.the instrument<br>9 = missing<br>8 = not administered/default<br><br><br>= 0 - 15<br>99 = missing<br>88 = not applicable/default<br><br><br>1 = complete and reliable<br>2 = technical problems<br>3 = refusal<br>4 = cognitive limitations<br>5 = physical limitations<br>6 = deviation fr.the instrument<br>9 = missing<br>8 = not administered/default	Irecall= e5_2697 + e5_2698<br>if e5_2697>10 → irecall=10 + e5_2698<br>if e5_2698>10 → irecall=e5_2697 + 10<br>if e5_2697>10 and e5_2698>10 → irecall=20<br><br>drecall= e5_2700<br>if drecall>10 → drecall=10	
MMSE				
LASA	MMSE (cmmsesc, bmmsesc)	Score Max. 30	Cmmsesc/bmmsesc=MMSE<br>If Cmmsesc/bmmsesc <0 → MMSE=missing	Variable name:<br>MMSE<br><br>Variable label:<br>Total MMSE score<br><br>Range 0-30
ActiFE Ulm	MMSE (mmse_BL)	Range 17-30	Use as is.<br>mmse_BL = MMSE	
TILDA	MMSE (COGmmse_ha)	Range 9-30	Use as is.<br>COGmmse_ha=MMSE	
B-PROOF	MMSE (MMSE_score)	Score Max. 30	MMSE_score=MMSE<br>If MMSE_score<0 → MMSE=missing	
Rotterdam Study	MMSE score (e5_3766)	Score max. 30	E5_3766=MMSE	

Gait speed				
LASA	Walk test: 2x3 meter walk and turn (as quickly as possible)<br>-time (BWALK04, CWALK04)<br><br><br>-reason test not done (BWALK02, CWALK02)	Range 3-64 sec<br>-1= missing<br><br>-1=See BWALK01<br>1=Respondent already walked<br>2=can walk (with aid)<br>3=can walk with aid not available<br>4=Cannot walk	**gait, wave C:**<br>if CWALK02 = 4 → gait = -1<br>if CWALK04 > 0 and CWALK02 ~= 4 → gait = 6 / CWALK04<br><br>**gait, wave 3B:**<br>if BWALK02 = 4 → gait = -1<br>if BWALK04 > 0 and BWALK02 ~= 4 → gait = 6 / BWALK04<br><br>**zgait**<br>Z-scores created for nonnegative gait values.	Variable name:<br>gait<br>zgait<br><br>Variable label:<br>Reported gait speed m/s<br>Z-scores of gait speed m/s – computed for each cohort individually<br><br>Value label:<br>gait<br>-1= Cannot walk<br><br>Notes:<br>- The instructions and reporting related the gait speed test differs per cohort. LASA and B-PROOF: as fast as possible. ERGO, TILDA, and ActiFE Ulm: normal speed. Z-scores were computed to make the cohorts comparable.
ActiFE Ulm	Short Physical Performance Battery (SPPB):<br>- Average gait speed (m/s) (SPPB_GS_BL)<br>- balance test (SPPB_b_cat_BL)<br>- time (sec) needed for Five-Chair-Rise test (SPPB_fcr_time_BL)<br>- walking aid (SPPB_walkaid_BL)<br>- gait speed test length (m) (SPPB_gs_length_BL)<br>- gait speed test: time needed for 1st run (sec) (SPPB_1run_BL)<br>- gait speed test: time needed for 2nd run (sec) (SPPB_2run_BL)<br>- gait speed test forerun (0:no forerun) (SPPB_gs_forerun_BL)<br>- gait speed test fastest time of the two runs (SPPB_gs_time_BL)<br>- gait speed test (categorised; 0:unable, 4:top fit) (SPPB_gs_cat_BL)<br>- five-chair-rise test (categorised; 0:unable, 4:top fit) (SPPB_fcr_cat_BL)<br>- SPPB sum score of the 3 categorised tests (SPPB_cat_sum_BL)<br>- SPPB frailty index (0:fit, 1:pre-frail, 2:frail) (SPPB_frailty_BL)	0=not able<br>4= top fit<br><br>0=not able<br>4= top fit<br><br>0=not able<br>4= top fit	**gait**<br>gait = SPPB_gs_BL<br>if SPPB_gs_cat_BL = 0 → gait = -1<br><br>**zgait**<br>Z-scores created for nonnegative gait values.	

TILDA	Timed up and go – rise, 2x3 meters and turn, sit down<br>-time (FRtugTimeSec)<br><br>Not available in public data wave 1:<br>-reason test not done (HOtugunabletocarryoutreason)<br><br>Gait speed was assessed using a computerised mat (active area 4.88 m) with embedded pressure sensors (GAITRite®, CIR Systems Inc, New York, USA). Respondents are asked to wear “normal walking shoes” preferably laced-up shoes or trainers. If respondents usually use assistive devices such as canes or walkers, they were advised to use them during the test which is recorded in the results software. Participants were asked to complete two walks along the mat at their usual walking pace. Participants were allowed a practice trial. The walking speeds obtained in the two walks in each condition are averaged to give usual gait speed. As participants started and finished 2.5 m before and 2 m after the walkway, it was assumed that the measured section represented steady state gait speed.<br>GRTspeed: Gait speed cm/s (normal timed walk)<br>GRT_Used_walking_aid: Was a walking aid used during the test<br>GRT_Gait_disturb_walk: Was the test interrupted after starting<br>gaitFileStatedG: Reason for GAIT assessment not being completed<br><br>Note: no variable available on reason test not done in public wave 1.	Range 4-105 sec	**gait**<br>gait = GRTspeed / 100<br>if gaitFileStatedG = “Unable” → gait = -1<br><br>**zgait**<br>Z-scores created for nonnegative gait values.	
B-PROOF	Timed walking test: participants were asked to walk 3 m, turn around and walk back as quickly as possible. (pp4)<br><br>Check walking ability (pp2)<br><br><br><br><br><br><br><br>Did R walk alone? (pp3)<br><br><br><br>Test terminated: reason (pp12)	Range 3-90 sec<br>-1=missing<br>400=measurement failed<br><br><br><br>1 = resp already walked<br>2 = can walk (with aid)<br>3 = can walk with aid, not available<br>4 = cannot walk<br><br>0 = no<br>1 = yes<br>2 = terminated<br><br>0 = Not terminated<br>1 = Not enough room/time<br>2 = R refused<br>3 = Exercise not safe<br>4 = Physically not capable<br>5 = R did not understand<br>6 = Other	**gait**<br>if pp4 > 0 → gait = 6 / pp4<br>if pp2 = 4 or pp12 = 4→ gait = -1<br><br>**zgait**<br>Z-scores created for non-negative gait values.	

Rotterdam Study	Gait assessment with a 5.79-m-long electronic walkway with 1.27-cm-wide pressure sensors (4.88×0.61-m active area) (GAITRite Platinum; CIR systems Inc., Sparta, NJ, USA): 3 items are scored during walking on the mat. Participants may keep on their shoes, except when they have high heels or because of other reasons (no variable names available, only nrs: 16400-16412):<br>^ A. Can you stand on the mat in the way you are used to?<br>^ B. Can you walk on the mat and exit the mat at the end?<br>^ And now the same instruction, but the other direction?<br>Repeat this 3 times.<br>^ C. Can you walk on the mat again, turn around at the end of the mat and then walk back?<br>^ D. Can you walk on the straight line (on the mat)?<br>Observe the walk and turn.<br><br>Centimeters/second (Velocity_first)<br><br>Centimeters/second (Velocity_second)<br>The second velocity measurement was taken at a later date, and is therefore less representative of respondents' gait speed at baseline.<br><br>Walking capacity?<br><br>Test on the mat completed?<br><br>Any missing parts?<br>Reden?	Range 21 - 186<br><br>Range 14 - 182<br><br>0 = normal<br>1 = almost normal, not in tandem position<br>2 = without support, abnormal & irregular<br>3 = without support, wobbling & difficult to turn<br>4 = not without autonomous support<br>5 = only possible with walking stick<br>6 = Only possible with 2 walking sticks or rollator<br>7 = Only with accompaniment<br>8 = walking impossible (wheelchair)<br>99 = missing<br>88 = not applicable/default<br><br>1 = yes, everything according to protocol<br>2 = no, not completely<br>3 = measurement not done<br>9 = missing<br>8 = default<br><br>String<br>8 = default	**gait**<br>gait = Velocity_first / 100<br><br>**zgait**<br>Z-scores created.	
Number of chronic conditions				
LASA	See Appendix 1		nchrdis=diabetes+heart+lung+cancer	Variable name:<br>nchrdis
ActiFE Ulm	See Appendix 1			

TILDA	See Appendix 1		See Appendix 1 for details on how the chronic diseases were selected for creating this summary score.<br>Please refer to disease specific sections to see how these variables were harmonized.	Variable label:<br>Number of chronic conditions<br><br>Range 0-4<br><br>Use of a summary score is not preferred as the number of measured chronic conditions differs per cohort.
B-PROOF	No data available, only diabetes and heart.			
Rotterdam Study	See Appendix 1			
**Number of medications**				
LASA	Respondents were asked to show their medication containers to the interviewers. Participants were asked to show only the medicines that were prescribed by a physician and that were used currently. The name, dose, frequency of intake, and duration of use of every medicine was recorded.<br>The number of recorded medications (BM#MED, CM#MED)	Range 0-19	nmed variable was created using the number of described medication.<br>if nmed >=8 → nmed = 8<br><br>if (nmed<5) → polypharmacy=0<br>if (nmed>=5) → polypharmacy=1	Variable name:<br>nmed<br>polypharmacy<br><br>variable label:<br>Number of medications<br>Polypharmacy (more than four types of medication)<br><br>Value label:<br>nmed<br>8=8 or more types of medication<br><br>polypharmacy<br>0=no<br>1=yes<br><br>Notes:<br>- see appendix for a list of all types of medications. For each of these types, a dichotomous variable will be included in the harmonized dataset.<br>- Supplements were removed from the number of medications (see appendix 8).
ActiFE Ulm	For each participant, every medication in use is described.	0-16	nmed variable was created using the number of described medication.<br>if nmed >=8 → nmed = 8<br><br>if (nmed<5) → polypharmacy=0.<br>if (nmed>=5) → polypharmacy=1.	
TILDA	Respondents were asked to show all medications taken; names were copied from the containers by the nurse (MDmeds).<br><br>Number of reported medications excluding supplements (MDmeds_excl_supps).	Range 0-14<br>NB: 14+ coded as 14.<br>82 missing	nmed= MDmeds_excl_supps<br>if nmed >=8 → nmed = 8<br><br>if (nmed<5) → polypharmacy=0.<br>if (nmed>=5) → polypharmacy=1.	
B-PROOF	For each participant, every medication in use is described.		nmed variable was created using the number of described medication.<br>if nmed >=8 → nmed = 8<br><br>if (nmed<5) → polypharmacy=0.<br>if (nmed>=5) → polypharmacy=1.	
Rotterdam Study	Medication use was registered during the home interview by trained research assistants; During the home interview, participants presented all the medication they used in the past week. Trained research assistants registered drug names, dose, and indication on a structured data entry form.<br>Variable for number of medications cannot be found in the variable list, but it is expected that this is available as medication use was measured for all participants. This should be asked separately.		nmed variable was created using the number of described medication.<br>if nmed >=8 → nmed = 8<br><br>if (nmed<5) → polypharmacy=0.<br>if (nmed>=5) → polypharmacy=1.	
**Number of people in household**				
LASA	**LASA C**<br>Number of other persons in household (excl. partner) (cnupers)<br><br>**LASA 3B**<br>Number of other persons in household (including partner) (bnupers)	Range 0-6	**LASA C**<br>If cnupers >=0 → npplhh= cnupers<br>if marital= 1 → npplhh = npplhh + 1*<br>if npplhh >= 3 → npplhh = 3<br><br>**LASA 3B**<br>If bnupers >=0 → npplhh= bnupers<br>If npplhh >=3 → npplhh=3<br><br>*) See marital status.	Variable name:<br>npplhh<br><br>Variable label:<br>Number of other people in the household<br><br>Value labels:<br>0=none<br>1=one<br>2=two<br>3=three or more

ActiFE Ulm	No data available.		Not harmonized.	
TILDA	Household size (includes partner) (hhsize)	Range 1-8<br>8+ coded as 8.<br>13 missing	npplhh= hhsize-1<br>If npplhh>=3 → npplhh=3	
B-PROOF	No data available.		Not harmonized.	
Rotterdam Study	How many people live in your household ? (e5_EIHOMEAP)<br><br>With whom are you living? (e5_EIHOMEW, e5_EIHOMEW2, e5_EIHOMEW3, e5_EIHOMEW4)	Number<br>77=don't know<br>88=not applicable<br>99=missing<br><br>1 = no one<br>2 = with partner<br>3 = with children<br>4 = with parents<br>5 = with brothers/sisters<br>6 = with others<br>7 = don't know<br>9 = no answer	npplhh=e5_EIHOMEAP-1<br>If e5_EIHOMEW = 1 → npplhh= 0<br>If npplhh>=3 → npplhh=3	
Occupation and retirement				
LASA	Paid job at present (bjob1, cjob1)<br><br>Hours per week (bjob6, cjob6)<br><br>(Partial) early retirement (bretired, cretired)	1=no<br>2=yes<br><br>Range 1-100<br><br>1=no<br>2=yes, partly<br>3=yes, completely	**job**<br>If bjob1/cjob1=1 → job=0<br>If bjob1/cjob1=2 → job=1	Variable name<br>job<br><br>Variable label<br>Current job status<br><br>Value label<br>0=not in paid job<br>1=in paid job
ActiFE Ulm	No data available.		Not harmonized.	
TILDA	Which one of these would you say best describes your current situation? (we001)<br><br>How many jobs do you currently have? (we102)<br><br>How many hours a week do you usually work in this job?<br>– most important job (we107)<br>– job 2 (we144_1)<br>– job 3 (we144_2)<br>– job 4 (we144_3)<br>– job 5 (we144_4)	1=retired<br>2=employed<br>3=self-employed<br>4=unemployed<br>5=permanently sick or disabled<br>6=looking after home or family<br>7=in education or training<br>95= other<br><br>Range 1-4<br><br>Range 0-168	**Job**<br>If we001=1 | we001=4 | we001=5 | we001=6 | we001=7 | we001=95 → job =0<br>If we001=2 | we001=3 → job=1	
B-PROOF	No data available		Not harmonized.	

Rotterdam Study	What is your current job status? (e5_EISES2)<br><br><br><br><br><br><br><br><br><br><br>What is your current job? (e5_EIJOBTY)<br><br>How many hours a week do you work? (e5_EIJOBHR)<br><br>In which year did you stop working? (e5_EISES2B)	0 = Payed office<br>1 = Unemployed (geregistreerd Gab)<br>2 = House wife/-man<br>3 = Incapacitated<br>4 = Rentier<br>5 = Early retirement<br>6 = retired (>= 65)<br>7 = don't know<br>9 = no answer<br><br>String<br><br>Hours<br><br>Year	**Job**<br>If e5_EISES2>=1 and e5_EISES2<=6 → job=0<br>If e5_EISES2=0 → job=1	
Pain				
LASA	Nottingham Health Profile questionnaire:<br>I am in pain when I am standing (bqpain1, cqpain1)<br>I find it painful to change position (bqpain2, cqpain2)<br>I am in pain when I am sitting (bqpain3, cqpain3)<br>I am in pain when I walk (bqpain4, cqpain4)<br>I have unbearable pain (bqpain5, cqpain5)<br>I am in constant pain (bqpain6, cqpain6)<br><br>Follow-up question to the above:<br>Evaluation of pain at present (bqpain, cqpain)<br><br><br><br><br>SF-12 pain item (<u>only in 3B</u>): During the past 4 weeks, how much did pain interfere with your normal work (including work outside the home and housework)? (BQQULI08)	 <br>1=yes<br>2=no<br><br><br><br><br><br><br><br><br><br><br><br>5=low (no pain)<br>6<br>7<br>8<br>9<br>10=high (severe pain)<br><br>1=not at all<br>2=a little bit<br>3=moderately<br>4=quite a bit<br>5=extremely	**pain**<br>If b/cqpain =5 → pain=0<br>If b/cqpain >=6 → pain=1<br><br>**painint**<br>If BQQULI08=1→ painint=0<br>If BQQULI08>=2 → painint=1	Variable name:<br>1. pain<br>2. painint<br><br>Variable labels:<br>1. Presence of pain<br>2. Do you experience pain that interferes with your work/normal activities?<br><br>Value labels<br>0=no<br>1=yes<br><br>Notes:<br>- Pain for ActiFE Ulm pain also includes backpain. With the inclusion of backpain, the harmonized distribution of pain in ActiFE Ulm is more in line with that of the other cohorts.<br>- The distribution of painint of TILDA differs greatly from that of the other cohorts. Unlike the other cohorts, the SF12 was not used in TILDA.

ActiFE Ulm	chronic pain until baseline (C_cpain_BL)<br>backpain until baseline (C_backpain_BL)<br><br>SF-12 pain item: During the past 4 weeks, how much did pain interfere with your normal work (including work outside the home and housework)? (SF12_Q8_BL)	0=no<br>1=yes<br><br>1=not at all<br>2=slightly<br>3=moderately<br>4=quite a bit<br>5=extremely	**pain**<br>If C_cpain_BL = 0 AND C_backpain_BL = 0 → pain = 0<br>If C_cpain_BL = 1 OR C_backpain_BL = 1 → pain = 1<br><br>**painint**<br>If SF12_Q8_BL =1 → painint=0<br>If SF12_Q8_BL>=2 → painint=1	
TILDA	Are you often troubled with pain? (ph501)<br><br>How bad is the pain most of the time? Is it... (ph502)<br><br><br>Does the pain make it difficult for you to do your usual activities such as household chores or work? (ph504)	1=yes<br>5=no<br><br>1=mild<br>2=moderate<br>3=severe<br><br>1=yes<br>5=no	**pain**<br>If ph501=5 → pain=0<br>If ph501=1 → pain=1<br><br>**painint**<br>If ph504=5 | ph501=5 → painint=0<br>If ph504=1 → painint=1	
B-PROOF	SF-12 pain item: During the past four weeks, how much did pain interfere with your normal work, including both work outside the home and housework? (SF12_8)<br><br>Euroqol item 4: range 1-3: pain or other complaints. (Euroqol_4)<br>Note that this item is somewhat broad.	1=not at all<br>2=a little bit<br>3=moderately<br>4=quite a bit<br>5=extremely<br><br><br>1=none<br>2=some<br>3=severe	**pain**<br>If Euroqol_4 = 1 → pain =0<br>If Euroqol_4>=2 → pain = 1<br><br>**painint**<br>If SF12_8=1 painint=0<br>If SF12_8>=2 and SF12_8<=5 → paintint=1	

Rotterdam Study	In the past 6 months, did you experience any pain? (e5_EIPIJN)<br><br><br><br><br><br><br><br>EUR-QoL question 4: pain/complaints (e5_EQOL4)<br><br><br><br><br><br><br><br>NB: No items on influence of pain on usual activities found.	0 = no<br>1 = yes, daily<br>2 = yes, weekly<br>3 = yes, a few times/ monthly<br>7 = don't know<br>9 = missing<br><br>1 = no pain or other complaints<br>2 = some pain or other complaints<br>3 = a lot of pain or other complaints<br>7 = no answer<br>9 = missing	**pain**<br>If e5_EIPIJN=0 → pain=0<br>If e5_EIPIJN=1 or e5_EIPIJN=2 or e5_EIPIJN=3 → pain=1<br><br>**painint**<br>Not harmonized.	
Parkinson's disease				
LASA	No data available.		Not harmonized.	
ActiFE Ulm	No data available.			
TILDA	No data available.			
B-PROOF	Data available?			
Rotterdam Study	Data available?			

Physical activity				
LASA	LASA Physical Activity Questionnaire (LAPAQ) asks about the frequency and duration engaged in each activity during the past 2 weeks.<br>In the past two weeks, did you do:<br>-walking outdoors (BLPHYA07, CLPHYA07)<br>-cycling (BLPHYA11, CLPHYA11)<br>-gardening (BLPHYA17, CLPHYA17)<br>-light household (BLPHYA32, CLPHYA32)<br>-heavy household (BLPHYA36, CLPHYA36)<br>-sport 1 (BLPHYA21, CLPHYA21)<br>-sport 2 (BLPHYA26, CLPHYA26)<br><br>In the past two weeks, how often did you:<br>-walking outdoors (BLPHYA08, CLPHYA08)<br>-cycling (BLPHYA12, CLPHYA12)<br>-gardening (BLPHYA18, CLPHYA18)<br>-light household (BLPHYA33, CLPHYA33)<br>-heavy household (BLPHYA37, CLPHYA37)<br>-sport 1 (BLPHYA23, CLPHYA23)<br>-sport 2 (BLPHYA27, CLPHYA27)<br><br>How much time did you spent doing this activity each time:<br>-walking outdoors (BLPHYA09, CLPHYA09)<br>-cycling (BLPHYA13, CLPHYA13)<br>-gardening (BLPHYA19, CLPHYA19)<br>-light household (BLPHYA34, CLPHYA34)<br>-heavy household (BLPHYA38, CLPHYA38)<br>-sport 1 (BLPHYA24, CLPHYA24)<br>-sport 2 (BLPHYA28, CLPHYA28)	1= yes<br>2= no	**LTPA**<br>*calculated time/week spent in each domain, then sum time spent walking, cycling, and sports, and dichotomise.<br>If sum<=10 → LTPA=0<br>If sum>10 → LTPA=1<br><br>**physact**<br>*minutes per week are calculated for each domain of activity, then multiplied by its respective MET value and summed.<br>physact=(walking*3.5)+(cycling*4.0)+<br>(heavy household*4.0)+(garden*4.0)+ (sport*6.0)<br><br>**physact3**<br>If (physact)<40 → physact3=0<br>If physact>=40 & physact<600 → physact3=1<br>If physact>=600 → physact3=2<br><br>**zphysact**<br>Z-scores created.	Variable names:<br>1. LTPA<br>2. physact<br>3. physact3<br>4. zphysact<br><br>Variable labels:<br>1. Leisure time physical activity<br>2. Physical activity (MET.min/week)<br>3. Physical activity level<br>4. Z-score for MET level<br><br>Value labels:<br>LTPA:<br>0=no<br>1=yes (≥1 time/week)<br><br>physact:<br>Range= 0- MET.minutes/week<br><br>physact3:<br>0=inactive (<40 MET.min/week)<br>1=low level of activity (40-<600 MET.min/week)<br>2=high level of activity (≥600 MET.min/week)<br><br>Note: all cohorts use LAPAQ except for TILDA (i.e. IPAQ). IPAQ includes quite different activities (walking, moderate and vigorous activities). IPAQ can be harmonized with the

ActiFE Ulm	LASA Physical Activity Questionnaire (LAPAQ)<br><br>physical activity: walking (1:yes, 0:no) (LAPAQ_walk_BL)<br>daily mean duration (min) of walking (LAPAQ_walk_daily_BL)<br>physical activity: cycling (1:yes, 0:no) (LAPAQ_cycl_BL)<br>daily mean duration (min) of cycling (LAPAQ_cycl_daily_B)<br>physical activity: gardening (1:yes, 0:no) (LAPAQ_garden_BL)<br>daily mean duration (min) of gardening (LAPAQ_garden_daily_BL)<br>physical activity: to do sports (1:yes, 0:no) (LAPAQ_sport_1st_BL)<br>daily mean duration (min) of most frequent sport (LAPAQ_sport_1st_daily_BL)<br>physical activity: to do more sports (1:yes, 0:no) (LAPAQ_sport_2nd_BL)<br>daily mean duration (min) of 2nd most frequent sport (LAPAQ_sport_2nd_daily_BL)<br>physical activity: light housework (1:yes, 0:no) (LAPAQ_light_hwork_BL)<br>daily mean duration (min) of light housework (LAPAQ_light_hwork_daily_BL)<br>physical activity: heavy housework (1:yes, 0:no) (LAPAQ_heavy_hwork_BL)<br>daily mean duration (min) of heavy housework (LAPAQ_heavy_hwork_daily_BL)<br>daily mean duration (min) spend on physical activity (LAPAQ_totact_BL)<br>daily mean duration (min) spend on physical activity without housework (LAPAQ_totact_nohwork_BL)<br>daily mean duration (min) spend on sports activity (LAPAQ_totact_sport_BL)<br>daily mean duration (min) spend on non-sports activity (LAPAQ_totact_nosport_BL)<br>number of different physical activities (LAPAQ_num_act_BL)		**LTPA**<br>* Calculated time/week spent in each domain, then sum time spent walking, cycling, and sports, and dichotomise.<br>If sum<=10 → LTPA=0<br>If sum>10 → LTPA=1<br><br>**physact**<br>*minutes per week are calculated for each domain of activity, then multiplied by its respective MET value and summed.<br>physact=(walking*3.5)+(cycling*4.0)+<br>(heavy household*4.0)+(garden*4.0)+ (sport*6.0)<br><br>**physact3**<br>If (physact)<40 → physact3=0<br>If physact>=40 & physact<600 → physact3=1<br>If physact>=600 → physact3=2<br><br>**zphysact**<br>Z-scores created.	rest by computing z-scores for the MET scores. Note: after merging the files, the z-scores for LAPAQ were computed across all cohorts that used the instrument.

TILDA	The International Physical Activity Questionnaire (IPAQ) (short-form) asks about the frequency and time spent in the following activities during the last week:<br>During the last 7 days, on how many days did you do this activity<br>-vigorous physical activity like heavy lifting, digging, aerobics, fast cycling (bh101)<br>-moderate activities that make you breathe somewhat harder than normal, like carrying light loads, cycling at a regular pace or doubles tennis? Do not include walking (bh103)<br>-walking, including at work and at home, to travel from place to place and other walking you might do for recreation, sport, exercise or leisure (bh105)<br><br>How much time did you usually spend doing this activity on one of those days:<br>-vigorous physical activities (hours: bh102, minutes: bh102a)<br>-moderate physical activities (hours: bh104, minutes: bh104a)<br>-walking (hours: bh106, minutes: bh106a)	1= Range 1-7<br>5= No I have not done this activity<br><br>Range hours: 0-10<br>Range minutes: 0-60	**LTPA**<br>*As the vigorous and moderate activity questions may include household and gardening activities, no LTPA variable could be created.<br><br>**Phyasact & physact3**<br>TILDA already includes a variable containing the MET score (IPAQmetminutes). Due to differences between the IPAQ and LAPAQ I do not think it is appropriate to use MET sores as cut-off points for levels of activity. Instead, I will create z-scores for the MET scores.<br><br>**zphysact**<br>Z-scores created.	

B-PROOF	LASA Physical Activity Questionnaire (LAPAQ): asks about physical activity in general and the frequency and duration engaged in each activity during the past 2 weeks.<br><br>In general, do you do the following activities:<br>-walking outdoors (lapaq_6)<br>-cycling (lapaq_10)<br>-gardening (lapaq_14, lapaq_15)<br>-light household (lapaq_31)<br>-heavy household (lapaq_34)<br>-sport 1 (lapa_20)<br>-sport 2 (lapaq_25)<br><br>In the past two weeks, did you do:<br>-walking outdoors (lapaq_7)<br>-cycling (lapaq_11)<br>-gardening (lapaq_16)<br>-sport 1 (LAPAQ_21)<br>-sport 2 (LAPA_26)<br><br>In the past two weeks, how often did you:<br>-walking outdoors (LAPAQ_8)<br>-cycling (LAPAQ_12)<br>-gardening (LAPAQ_17)<br>-light household (LAPAQ_32)<br>-heavy household (LAPAQ_35)<br>-sport 1 (LAPAQ_23)<br>-sport 2 (LAPAQ_28)<br><br>How much time did you spent doing this activity:<br>-walking outdoors (lapaq_9)<br>-cycling (lapaq_13)<br>-gardening (lapaq_18)<br>-light household (lapaq_33)<br>-heavy household (lapaq_36)<br>-sport 1 (lapaq_24)<br>-sport 2 (lapaq_29)	 <br><br>0=no<br>1=yes<br><br>Also: # of months in year<br><br><br><br><br><br><br>0=no<br>1=yes<br><br><br><br><br><br># of times/days, range<br><br><br><br><br><br><br><br><br>Time in minutes, range	**LTPA**<br>*calculated time/week spent in each domain, then sum time spent walking, cycling, and sports, and dichotomise.<br>If sum<=10 → LTPA=0<br>If sum>10 → LTPA=1<br><br>**physact**<br>*minutes per week are calculated for each domain of activity, then multiplied by its respective MET value and summed.<br>physact=(walking*3.5)+(cycling*4.5)+<br>(heavy household*4.0)+(garden*4.0)+ (sport*6.0)<br><br>**physact3**<br>If (physact)<40 → physact3=0<br>If physact>=40 & physact<600 → physact3=1<br>If physact>=600 → physact3=2<br><br>**zphysact**<br>Z-scores created.	

Rotterdam Study	In ERGO-5 the LASA Physical Activity Questionnaire was used to measure total PA, walking, cycling, domestic work, sports, gardening.<br><br>In general, do you do the following activities:<br>-walking outdoors (e5_15995)<br>-cycling (e5_16000)<br>-gardening (e5_16005, e5_16006)<br>-light household (e5_16091)<br>-heavy household (e5_16095)<br>-sport 1 (e5_16012 – e5_16085)<br>-sport 2 (e5_16086 - e5_16090)<br><br>In the past two weeks, did you do:<br>-walking outdoors (e5_15996)<br>-cycling (e5_16001)<br>-gardening (e5_16007)<br>-sport 1 (e5_16012 – e5_16085)<br>-sport 2 (e5_16086 - e5_16090)<br><br>In the past two weeks, how often did you:<br>-walking outdoors (e5_15997)<br>-cycling (e5_16002)<br>-gardening (e5_16008)<br>-light household (e5_16092)<br>-heavy household (e5_16096)<br>-sport 1 (e5_16012 – e5_16085)<br>-sport 2 (e5_16086 - e5_16090)<br><br>How much time did you spent doing this activity:<br>-walking outdoors (e5_15998, e5_15999)<br>-cycling (e5_16003, e5_16004)<br>-gardening (e5_16009, e5_16010)<br>-light household (e5_16093, e5_16094)<br>-heavy household (e5_16097)<br>-sport 1 (e5_16012 – e5_16085)<br>-sport 2 (e5_16086 - e5_16090)	0=no<br>1=yes<br>8=default<br>9=missing<br><br>0=no<br>1=yes<br>8=default<br>9=missing<br><br>88=default<br>99=missing<br><br>88=default<br>99=missing	**LTPA**<br>*calculated time/week spent in each domain, then sum time spent walking, cycling, and sports, and dichotomise.<br>If sum<=10 → LTPA=0<br>If sum>10 → LTPA=1<br><br>**physact**<br>*minutes per week are calculated for each domain of activity, then multiplied by its respective MET value and summed.<br>physact=(walking*3.5)+(cycling*4.5)+<br>(heavy household*4.0)+(garden*4.0)+ (sport*6.0)<br><br>**physact3**<br>If (physact)<40 → physact3=0<br>If physact>=40 & physact<600 → physact3=1<br>If physact>=600 → physact3=2<br><br>**zphysact**<br>Z-scores created.	
Pulse rate				
LASA	**LASA C**<br>Pulse rate sitting (arm) (cmvar802)<br>Pulse rate lying (arm) (cmvar805)<br>Pulse rate standing (arm) (cmvar808)<br><br>**LASA 3B**<br>Pretest 1: Pulse rate sitting (arm) (BMARMPS01)<br>Pretest 2: Pulse rate sitting (arm) (BMARMPS02)<br>Pulse rate 1 sitting (arm) (BMARMPS1)<br>Pulse rate 2 sitting (arm) (BMARMPS2)	-2/-1 = missing	**Wave C**<br>Use as is.<br>If (cmvar802>0) pulse = cmvar802<br><br>**Wave 3B**<br>Use as is.<br>If (BMARMPS01>0) pulse = BMARMPS01	Variable name:<br>pulse<br><br>Variable label:<br>Pulse rate (beats/min)<br><br>In case of multiple measurements, the first measurement was taken. It is unclear from which position pulse rate was measured in ULM and B-Proof. Nonetheless, pulse rate is typically measured from sitting position.

ActiFE Ulm	pulse (beats/min) [1. measurement] (pulse_1_BL)<br>pulse (beats/min) [2. measurement] (pulse_2_BL)<br>pulse (beats/min) [3. measurement] (pulse_3_BL)		Use as is.<br>pulse = pulse_1_BL	
TILDA	No data available.		Not harmonized.	
B-PROOF	Heart rate measured with datascoop during vascular measurement (beats / min) (HR_datascoop)		Use as is.<br>pulse = HR_datascoop	
Rotterdam Study	Heart rate 1 in sitting position (in beats/min) (e5_15643)<br>Heart rate 2 in sitting position (in beats/min) (e5_15644)		Use as is.<br>If not missing (e5_15643) pulse = e5_15643	
Quality of life				
LASA	**LASA C & 3B:**<br>In the self-administered LASA questionnaire, an abbreviated version of the GHPQ is included (General Health Perceptions Questionnaire). This version consists of eight questions, four about current health perception, and four about the expectation of future health:<br>In the future I will probably be sick a lot (QHEALTH1)<br>I don't feel well (QHEALTH2)<br>In the future I expect to be healthier than other people I know (QHEALTH3)<br>I am just as healthy as other people I know (QHEALTH4)<br>In the future, I think my health will be worse than now (QHEALTH5)<br>My health is excellent (QHEALTH6)<br>I expect to live a very healthy life (QHEALTH7)<br>I don't feel well the past couple of days (QHEALTH8)<br><br>**LASA 3B:**<br>Besides GHPQ, also includes EuroQol (BQEQ5D1… BQEQ5D5 & BQEQVAS)<br><br>And: Euroquol TTO-method UK or NL index. (bqeqixuk, bqeqixnl)<br><br>Also includes SF-12 Health Survey (BQQULI01… BQQULI012, BQQULIPS, BQQULIMS, BQQULISF, BPCS12, BMCS12)<br><br>Physical Component score (SF-12) (BPCS12) → according to official manual (American)<br>Mental Component score (SF-12) (BMCS12) → according to official manual (American)	-1= no answer<br>1= strongly disagree<br>2= disagree<br>3=no agreement/disagreement<br>4= agree<br>5= strongly agree<br><br>Range 1-3:<br>Mobility<br>Self-care<br>Daily activities<br>Pain or other complaints<br>Mood<br><br>Score your health (0-100)<br><br>-2=no valid score<br>1=perfect health status<br><br>Range, -1=no scale (mv)<br><br>Range, -1=no scale (mv)	**Euroqol**<br>If bqeqixnl>0 → euroqol= bqeqixnl<br><br>**MCS**<br>If BMCS12>0 → MCS=BMCS12<br><br>**PCS**<br>If BPCS12>0 → MCS=BPCS12	Variable names:<br>1. euroqol<br>2. MCS<br>3. PCS<br><br>Variable label:<br>1. Euroqol summary score<br>2. SF12 - Mental Component Score<br>3. SF12 - Physical Component Score<br><br>LASA C only includes the GHPQ and no Euroqol or SF-12, so is not harmonized. TILDA only uses CASP-19. GHPQ and CASP-19 are clearly different measures than SF-12 and Euroqol; GHPQ measures general perception on current and future health, and CASP-19 measures a fuller range of QoL (not only health-related QoL, also control, autonomy, pleasure, self-realization). Both are therefore not comparable with SF-12 and Euroqol.<br><br>For the scoring of the EQ-5D instrument, the Dutch TTO-method by Lamers et al. (2006) was used. Specifically, we used the regression coefficients of the 'N3', gives additional weight to having at least one EQ-5D dimension at the worst level. Out of the models in Lamers' paper, the N3 model had the best predictive performance.
ActiFE Ulm	SF-12 questionnaire (SF12_Q1_BL … SF12_Q12_BL)		**Euroqol**<br>Not harmonized.<br><br>**MCS & PCS**<br>Harmonized using LASA's syntax.	

TILDA	CASP-19 questionnaire	Range 0-57	Not harmonized.	
B-PROOF	Short form 12 health survey (SF-12) is used (SF12_1…SF12_12)<br><br>Also, the EuroQol (EQ-5D) is used (Euroqol_1…Euroqol_5 & Euroqol_scale)	Range 1-3:<br>Mobility<br>Self-care<br>Daily activities<br>Pain or other complaints<br>Mood<br><br>Score your health (0-100)	**Euroqol**<br>Harmonized using LASA's syntax.<br><br>**MCS & PCS**<br>Harmonized using LASA's syntax.	
Rotterdam	EuroQoL questionnaire (e5_EQOL1, e5_EQOL2, e5_EQOL3, e5_EQOL4, e5_EQOL5)<br><br>Other 6th item on QOL: e5_EQOL6, Range 0-100	Range 1-3<br>Mobility<br>Self-care<br>Daily activities<br>Pain or other complaints<br>Mood<br><br>1=I have no problems with walking<br>2=I have some problems with walking<br>3=I'm immobile (bedlegerig)<br>7=no answer<br>9=missing<br><br>777=don't know<br>888=not applicable<br>999=missing	**Euroqol**<br>Harmonized using LASA's syntax.<br><br>**MCS & PCS**<br>Not harmonized.	
Reaction time				
LASA	No data available		Not harmonized.	Data not harmonized as there are fewer than 3 studies with relevant data.
ActiFE Ulm	No data available			
TILDA	Choice reaction time task: participants were asked to press a NO button if 'no' appeared and a YES button if 'yes' appeared on the screen. The mean response time was taken. (wave 1, 3: CRTcog)	Range		
B-PROOF	No data available			
Rotterdam Study	No data available	= 0.000 - 70.000<br>99.999 = missing<br>88.888 = not applicable/default		

Self-rated health				
LASA	Self-perceived health (bsubhea1, csubhea1)	-1=missing<br>1=excellent<br>2=good<br>3=fair<br>4=sometimes good/ sometimes bad<br>5=poor	**SRH**<br>if (bsubhea1>0) SRH= bsubhea1<br><br>**SRHdich**<br>If bsubhea1=4 or bsubhea1=5 → SRHdich=1<br>If bsubhea1=1 or bsubhea1=2 or bsubhea1=3 → SRHdich=0	Variable name:<br>1.SRH<br>2.SRHdich<br><br>Variable label:<br>1.Self-rated health (1: excellent; 5: poor)<br>2.Self-rated health (good versus poor; 0 = good health, 1 = poor health)<br><br>Value labels<br><br>The labelling of the categories differs in LASA from the other cohorts. The distribution also differs. I have left the categories as is for each cohort, but also choose to dichotomise as the lowest 2 (i.e., worst possible ratings) vs. the highest 3 categories (i.e., best possible rating).<br><br>Note that for ERGO, the VAS of the Euroqol was used. To my knowledge, there is no paper that describes converting the VAS-scale to a five-point or two-point scale for self-rated health. Therefore, I decided to not harmonize self-rated health for the Rotterdam Study.
ActiFE Ulm	SF-12 item: evaluation of your health in general? (SF12_Q1_BL)<br><br>self-assessed health (from SF_12) (health_BL)	1=excellent<br>2=very good<br>3=good<br>4=fair<br>5=poor<br><br>0=good<br>1=bad	**SRH**<br>Use as is.<br>SRH = (SF12_Q1_BL)<br><br>**SRHdich**<br>If SF12_Q1_BL =4 or SF12_Q1_BL =5 → SRHdich=1<br>If SF12_Q1_BL =1 or SF12_Q1_BL =2 or SF12_Q1_BL =3 → SRHdich=0	
TILDA	Would you say your health is … (ph001)	1=excellent<br>2=very good<br>3=good<br>4=fair<br>5=poor	**SRH**<br>SRH=ph001<br><br>**SRHdich**<br>If ph001=4 or ph001=5 → SRHdich=1<br>If ph001=1 or ph001=2 or ph001=3 → SRHdich=0	
B-PROOF	SF-12 item: evaluation of your health in general? (SF12_1)<br><br>Euroqol item: score your health. (Euroqol_scale)	1=excellent<br>2=very good<br>3=good<br>4=fair<br>5=poor<br><br>Range 0-100	**SRH**<br>SRH=SF12_1<br><br>**SRHdich**<br>If SF12_1=4 or SF12_1=5 → SRHdich=1<br>If SF12_1=1 or SF12_1=2 or SF12_1=3 → SRHdich=0	
Rotterdam Study	How do you view your own health on this moment, compared to your peers? (e5_EIGEZLFT)<br><br>The final question of euroqol asks partcipants to rate their health on a 0-100 scale (e5_EQOL6)	1 = better<br>2 = the same<br>3 = worse<br>7 = don't know<br>9 = missing	Not harmonized.	
Sex				
LASA	Sex	1=male<br>2=female	If sex=1 → sex=0<br>If sex=2 → sex=1	Variable name:<br>sex<br><br>Variable label:<br>0=male<br>1=female
ActiFE Ulm	sex	1=male<br>2=female	If sex=1 → sex=0<br>If sex=2 → sex=1	
TILDA	sex	1=male<br>2=female	If sex=1 → sex=0<br>If sex=2 → sex=1	
B-PROOF	gender	1=men<br>2=women	If gender=1 → sex=0<br>If gender=2 → sex=1	

Rotterdam Study	sex	0=male<br>1=female	Use as is.	
Sleep				
LASA	What time do you go to sleep (only 3B)<br>-hour of the day (bqsleeph)<br>-minute (bqsleepm)<br><br>Total minutes of sleep in 24 hours (bqsleep1, cqsleep1)<br><br>Do you have problems with falling asleep (bqsleep2, cqsleep2)<br>Do you have problems with continuing sleep (bqsleep3, cqsleep3)<br>Do you have problems with waking too early? (bqsleep4, cqsleep4)<br><br>Only 3B: How would you rate your sleep quality in the past month (bqsleep5)<br><br>Derived scale based on 3 categorical questions about sleep problems (bqsleep, cqsleep)	 Range 1-24<br>Range 0-46<br><br>Range 180-900<br><br>1=almost never<br>2=some of the time<br>3=often<br>4=most of the time<br><br>1=very good<br>2=somewhat good<br>3=somewhat bad<br>4=very bad<br><br>Range 3-12<br>3=no problems<br>12=many problems	**sleep, wave C:**<br>If cqsleep2=1 and cqsleep4=1 → sleepp=0<br>If (cqsleep2>=2 and cqsleep2<=4) or (cqsleep2>=2 and cqsleep2<=4) → sleepp=1<br><br>**sleep, wave 3B:**<br>If bqsleep2=1 and bqsleep4=1 → sleepp=0<br>If (bqsleep2>=2 and bqsleep2<=4) or (bqsleep2>=2 and bqsleep2<=4) → sleepp=1	Variable name:<br>sleepp<br><br>Variable label:<br>Sleeping problems (Waking up too early and/or having difficulty falling asleep)<br><br>Value labels:<br>0=no<br>1=yes
ActiFE Ulm	No data available.		Not harmonized.	
TILDA	How likely are you to doze off or fall asleep during the day? (bh201)<br><br>How often do you have trouble falling asleep? (bh202)<br><br>How often do you have trouble with waking up too early and not being able to fall asleep (bh203)	1=would never doze<br>2=slight chance of dozing<br>3=moderate chance of dozing<br>4=high chance of dozing<br><br>1=most of the time<br>2=sometimes<br>3=rarely or never<br><br>1=most of the time<br>2=sometimes<br>3=rarely or never	If bh203=3 and bh202=3 → sleepp=0<br>If bh203<=2 or bh202<=2 → sleepp=1	
B-PROOF	No data available.		Not harmonized.	

Rotterdam Study	Subjective sleep quality was assessed with the Pittsburgh Sleep Quality Index (PSQI). The PSQI is a self-rating questionnaire which measures sleep quality and disturbance retrospectively over a 1-month period, resulting in a global score between 0 and 21, with higher scores indicating poorer sleep quality.<br><br>During the past month;<br>… At what time did you go to bed most of the time? (e5_EIPSQ1)<br>… How long did it take you to fall asleep most of the time? (in min) (e5_EIPSQ2)<br>… At what time did you wake up? (e5_EIPSQ2A)<br>… At what time did you get up most of the time? (e5_EIPSQ3)<br><br>… How long do you usually sleep in the night (in hours)? (e5_YIPSQ4)<br>… How long do you usually sleep on the day (purposefully nap)? (e5_YIPSQ4A)<br><br>In the past year, have you been to your general practitioner for sleeping problems? (e5_YIPSQ5)<br><br>During the past month, how often did you sleep poorly because:<br>… you couldn't fall asleep within 30 minutes (e5_YIPSQ5A)<br>… you woke up in the middle of the night….? e5_YIPSQ5B)<br>… you had to go to the bathroom (e5_YIPSQ5C)<br>… you had difficulty breathing (e5_YIPSQ5D)<br>… you were coughing or snoring (e5_YIPSQ5E)<br>… you were too cold (e5_YIPSQ5F)<br>… you were too hot (e5_YIPSQ5G)<br>… you had bad dreams (e5_YIPSQ5H)<br>… you were in pain (e5_YIPSQ5I)<br>Were there other reasons you slept poorly? (e5_YIPSQ5J)<br><br>What reasons? (e5_YIPSQ5JX)<br><br>How would you rate your sleep over the past month? (e5_YIPSQ6)	Time<br><br>Hours<br><br>0 = no<br>1 = yes<br>7 = don't know<br><br>0 = not once during the past month<br>1 = < once a week<br>2 = 1 or 2 times a week<br>3 = > 2 times a week<br>7 = don't know<br>9 = no answer<br><br>String<br><br>0 = very good<br>1 = relatively good<br>2 = relatively bad<br>3 = very bad<br>7 = don't know<br>9 = no answer	If e5_YIPSQ5B=0 & e5_YIPSQ5A=0 → sleepp=0<br>If (e5_YIPSQ5B=1 or e5_YIPSQ5B=2 or e5_YIPSQ5B=3) or (e5_YIPSQ5A=1 or e5_YIPSQ5A=2 or e5_YIPSQ5A=3) → sleepp=1	
Smoking				
LASA	Do you smoke? (BMVAR31, CMVAR31)<br>Did you smoke before? (BMVAR32, CMVAR32)	1=no<br>2=yes	**smoke, wave C**<br>If CMVAR31=1 and CMVAR32=1 → smoke=0<br>If CMVAR31=1 and CMVAR32=2 → smoke=1<br>If CMVAR31=2 → smoke=2<br><br>**smoke, wave 3B**	Variable name:<br>smoke<br>smokeyn<br><br>Variable label:

			If BMVAR31=1 and BMVAR32 =1 → smoke=0<br>If BMVAR31=1 and BMVAR32 =2 → smoke=1<br>If BMVAR31=2 → smoke=2<br><br>**smokeyn, wave C**<br>If CMVAR31=1 → smokeyn=0<br>If CMVAR31=2 → smokeyn=1<br><br>**smokeyn, wave 3B**<br>If BMVAR31=1 → smokeyn=0<br>If BMVAR31=2 → smokeyn=1	Smoking status (never, ex-smoker, current smoker)<br>Do you smoke? (yes/no)<br><br>Value labels:<br>0=never smoked<br>1=ex-smoker<br>2=current smoker<br><br>Value labels:<br>0=no<br>1=yes
ActiFE Ulm	Smoking (smoker_BL)	0=never,<br>1=former,<br>2=current	**smoke**<br>Use as is.<br>smoke= smoker_BL<br><br>**smokeyn**<br>if smoker_BL=0 or smoker_BL=1 → smokeyn=0<br>if smoker_BL=2→ smokeyn=1	
TILDA	Have you ever smoked cigarettes, cigars, cigarillos or pipe daily for a period of at least 1 year? (bh001)<br><br><br>Do you smoke at the present time? (bh002)	 1=yes<br>5=no<br><br>1=yes<br>5=no, I have stopped	**smoke**<br>If bh001=5 → smoke=0<br>If bh001=1 & bh002=5 → smoke=1<br>If bh002=1 → smoke=2<br><br>**smokeyn**<br>if bh001=5 or bh002=5 → smokeyn=0<br>if bh002=1 → smokeyn=1	
B-PROOF	Do you smoke cigarettes, cigars, other tabacco? (smoking1)<br><br><br><br>Do you smoke cigarettes? (smoking_cigarettes)<br><br><br>Number of cigarettes a day? (nr_cigarettes)<br><br>Do you smoke cigars? (smoking_cigars)<br><br><br>Number of cigars? (nr_cigars)	0=have never smoked<br>1=smoke now<br>2=have smoked in the past<br><br>0=no<br>1=yes<br><br># of cigarettes<br><br>0=no<br>1=yes<br><br># of cigars	**smoke**<br>If smoking1=0 → smoke=0<br>If smoking1=1 → smoke=2<br>If smoking1=2 → smoke=1<br><br>**smokeyn**<br>If smoking1=0 or smoking1=2 → smokeyn=0<br>If smoking1=1 → smokeyn=1	

Rotterdam Study	Do you smoke cigarettes? (e5_EILF6)<br><br>Have you smoked cigarettes in the past? (e5_EILFE)<br><br>Do you smoke cigars? (If not, ask): Have you never smoked cigars in the past or have you quit smoking cigars? (e5_EILF4)<br><br>Do you smoke a pipe? (Zo nee, vraag): (If not, ask): Have you never smoked a pipe in the past or have you quit smoking pipe? (e5_EILF5)	0 = no<br>1 = yes<br>7 = don't know<br>9 = no answer<br><br>0 = no<br>1 = yes<br>7 = don't know<br>8 = n.a.<br>9 = no answer<br><br>0 = no, never<br>1 = no, small cigars in past<br>2 = no, large cigars in past<br>3 = no, small and large cigars in past<br>4 = yes, small cigars<br>5 = yes, large cigars<br>6 = yes, small and large cigars<br>7 = don't know<br>9 = no answer<br><br>0 = No, never<br>1 = no, but in the past I did<br>2 = yes<br>7 = don't know<br>9 = no answer	**smoke**<br>If e5_EILF6=0 and e5_EILFE=0 → smoke=0<br>If e5_EILF6=0 and e5_EILFE=1 → smoke=1<br>If e5_EILF6=1 → smoke=2<br><br>**smokeyn**<br>If e5_EILF6=0 → smokeyn=0<br>If e5_EILF6=1 → smokeyn=1	
Speed of processing				
LASA	Coding Task: adjusted version of the Alphabet Coding Task-15 (Savage, 1984), a letter substitution task described by Piccinin and Rabbitt (1999). In this task two rows of characters are shown, each character in the upper row belongs to a character in the bottom row. The test contains also two rows, one of them containing characters and the bottom row is empty. The respondent has to complete as many character combinations as possible, by naming the corresponding character. This was done in three cycles of one minute. The test measures speed of processing/reaction time and working memory. The mean score for the three trials is used in the analyses. (BMVAR47, BMVAR48, BMVAR49; CMVAR47, CMVAR48, CMVAR49)	Range 9.33-47.67	**Zprocspeed**<br>meanproc = -1 * mean (BMVAR47, BMVAR48, BMVAR49 // CMVAR47, CMVAR48, CMVAR49)<br><br>Create z-scores of meanproc for both waves individually	Variable names:<br>Zprocspeed<br><br>Variable label:<br>Z-score processing speed<br><br>Note: there are three cohorts with a coding task LASA C, 3B and ERGO. These are harmonized.<br><br>The coding/letter-digit tasks (LASAC/3B, ERGO) is most comparable with the Colour trail 2 test (TILDA, B-PROOF), but not a great match. We decided to make a Z-score combining de coding/letter-digit task with colour-trail task.
ActiFE Ulm	No data available.		Not harmonized.	

TILDA	Colour trail 2 test, participants were asked to connect numbers from 1 to 23 in alternating colours in consecutive order as quickly as possible. This test measures visual speed of processing, executive function. (wave 1, 3: COGtrail1time COGtrail2time)<br><br>Choice reaction time task: participants were asked to press NO if 'no' appeared and YES if 'yes appeared on the screen. The mean cognitive response time was taken. This test measures speed of processing/reaction time. (wave 1, 3: CRTmeancog)<br><br>Sustained attention to response task (SART): participants were seated in front of a screen that repeatedly displayed the digits 1 to 9 during 4 minutes. Participants were asked to press a key as quickly as possible every time a digit appeared, except when the number 3 appeared. This test measures processing speed and inhibition. (wave 1, 3: COGsartRawMean, COGsartCookedMean)	Range 234.65-3274.40	**Zprocspeed**<br>Meanproc = mean(COGtrail1time, COGtrail2time)<br><br>Create z-scores for meanproc	Scores in LASA were inverted by multiplying them by -1, so that scores are comparable across cohorts.age
B-PROOF	Trail Making Test (TMT) item A en B (Time_sec_TMT_A, TMT_A_corrections, Time_sec_TMT_B, TMT_B_corrections)<br><br>Stroop colour test (card 1 (words), 2 (colours), 3 (words & colours) (Time_sec_Stroop_Card1, Stroop_Card1_corrections, Time_sec_Stroop_Card2, Stroop_Card2_corrections, Time_sec_Stroop_Card3, Stroop_Card3_corrections)	Time in seconds spent on test (max. 300 s) and # of corrections made by interviewer<br><br>Time in seconds spent on test (max. 300 s) and # of corrections made by interviewer	**Zprocspeed**<br>Meanproc = mean(Time_sec_TMT_A, Time_sec_TMT_B)<br><br>Create z-scores for meanproc	
Rotterdam Study	Letter-digit test:<br>Number of attempts Letter-Digit test (e5_2743)<br><br>Correct answers Letter-Digit test (e5_2744)<br><br>Stroop test (e5_16505, e5_2720 … e5_2727)	= 0 - 80<br>99 = missing<br>88 = not admin. – default<br>= 0 - 80<br>99 = missing<br>88 = not admin. – default	**Zprocspeed**<br>Create z scores for sum of stroop test, based on total time needed for stroop test.	
Stroke				
LASA	self report: CVA (b3_srCVA/c_srCVA)<br>gp diagnosis: CVA (b3_gpCVA/c_gpCVA)<br><br>Algorithm used for determining stroke (see LASA website) (c_alg_CVA / b3_CVA)	-1 = missing<br>0 = no<br>1 = yes<br>2 = possible<br><br>-1 = missing<br>0 = no<br>1 = definitive<br>2 = possible<br>3 = contradictory	**C:**<br>If c_srCVA =0 → stroke = 0<br>If c_srCVA = 1 → stroke = 1	Variable name:<br>stroke<br><br>Variable label:<br>History of stroke<br><br>Value label:<br>0=no<br>1=yes
ActiFE Ulm	stroke until baseline (C_stroke_BL)	0=no<br>1=yes	Use as is.<br>C_stroke_BL=stroke	
TILDA	Has a doctor ever told you that you have any of the following conditions: stroke (ph201_06)	0=no<br>1=yes	ph201_06=stroke	

B-PROOF	Did participant ever had a stroke or TIA? (TIA)<br>Note that this variable also includes TIA.	0=no<br>1=yes	Use as is.<br>TIA=stroke	
Rotterdam Study	Heeft u na het vorige ERGO interview een beroerte, hersenbloeding, herseninfarct of CVA gehad? (e5_EICVA01)<br>Heeft U ooit een beroerte of hersenbloeding doorgemaakt die door een arts werd vastgesteld ? (ep_pimc15)<br>Stroke after Ergo1? (e2_b1cva)<br>Heeft hij/zij ooit een beroerte gehad (e3_3832)<br>Heeft hij/zij ooit een beroerte gehad (ep_3832)<br>Heeft hij/zij ooit een beroerte gehad (e4_3832)<br><br>From Medical history questionnaire:<br>After the previous ERGO interview, have you had a stroke, cerebral hemorrhage, cerebral infarction or CVA? (e5_EICVA01)	0 = no<br>1 = yes<br>7 = don't know<br>9 = no answer	if e5_EICVA01 = 0 or ep_pimc15 = 0 or e2_b1cva = 0 or e3_3832 = 0 or ep_3832 = 0 or e4_3832 = 0 → stroke = 0.<br>if e5_EICVA01 = 1 or ep_pimc15 = 1 or e2_b1cva = 1 or e3_3832 = 1 or ep_3832 = 1 or e4_3832 = 1 → stroke = 1.	
Urbanisation				
LASA	Level of urbanisation expressed as mean number of addresses per squared kilometre within a 1km radius (Burb, Curb)<br><br>Data are provided by Statistics Netherlands.<br>http://www.lasa-vu.nl/themes/demographics/urban-rural.html	1= not (<500)<br>2=little (500-1000)<br>3=somewhat (1000-1500)<br>4=highly (1500-2500)<br>5=very highly (>=2500)	If Burb>=3 & Burb<=5 → urban=0<br>If Burb=1 or burb=2 → urban=1	Variable name:<br>urban<br><br>Variable label:<br>Level of urbanisation<br><br>Value label:<br>0=urban<br>1=rural/remote
ActiFE Ulm	No data available.		Not harmonized.	
TILDA	Is the dwelling located: (local3)	1=Dublin city or county<br>2=a city or town in the Republic of Ireland other than Dublin<br>3=In a rural part of the Republic of Ireland	If local3=1 | local3=2 → urban=0<br>If local3=3 → urban=1	
B-PROOF	No data available		Not harmonized.	
Rotterdam Study	All urban areas		Urban=0	
Urinary incontinence				
LASA	Do you have incontinence? (BINCON1, CINCON1)<br><br>How often do you have loss of urine? (BINCON5, CINCON5)	<u>3B:</u><br>1=no<br>2=yes<br><br><u>C:</u><br>0=no, never<br>1=no, but at wave B yes<br>2=yes, but at wave B no<br>3=yes, and at wave B yes<br><br>1=≤2 per month<br>2=3-4 times per month<br>3=a few times per week<br>4=daily	**C:**<br>If CINCON1=0 → urine=0 or<br>If CINCON1=1 → urine=0<br><br>If (CINCON1=2 or CINCON1=3) → urine=1<br><br>**3B:**<br>If BINCON1= 1 → urine=0<br>If BINCON1=2 → urine=1	Variable name<br>urine<br><br>Variable label:<br>Presence of urinary incontinence<br><br>Categories:<br>0=no<br>1=yes
ActiFE Ulm	urinary incontinence (incontinence_BL)	0=no<br>1=yes	Used as is.<br>incontinence_BL=urine	

TILDA	PH601: During the last 12 months, have you lost any amount of urine beyond your control? (ph601)<br>PH602: Did this happen more than once during a 1 month period? (ph602)	1=yes<br>5=no	If ph601=1→ urine=1<br>If ph601=5 → urine=0	
B-PROOF	No data available		Not harmonized.	
Rotterdam Study	Kunt u uw plas goed ophouden (e3_3613)<br>Kunt u uw plas goed ophouden (ep_3613)<br>Kunt u uw plas goed ophouden (e4_3613)<br>Kunt u uw plas goed ophouden (ej_3613)	0 = ja<br>1 = soms nat<br>2 = vaak nat<br>8 = geen antwoord/weet niet<br>9 = not applicable/missing	Not harmonized as there is no data from the ergo-5 wave.	
Visual problems				
LASA	**LASA C & 3B: self-reported:**<br>Usually wears glasses or contact lenses (bsens01, csense01)<br><br>Subjective vision: do you see well enough? (bsens02, csense02)<br>Can you read small print in a paper without glasses etc? (bsens03, csense03)<br>Can you read small print in a paper with glasses etc? (bsens04, csense04)<br>Can you recognize a face at 4 meters distance without glasses? (bsens05, csense05)<br>Can you recognize a face at 4 meters distance with glasses? (bsens06, csense06)<br><br>**Acuity was measured in 3B**	1=no<br>2=yes<br><br>1=yes, without difficulty<br>2=yes, with some difficulty<br>3=yes, with much difficulty<br>4=no I cannot	**VisionNear**<br>If bsens01=1 & bsens03=1 → VisionNear=0<br>If bsens01=2 & bsens04=1 → VisionNear=0<br>If bsens01=1 & bsens03>1 → VisionNear=1<br>If bsens01=2 & bsens04>1 → VisionNear=1<br>If bsens01=2 & bsens04=missing & bsens03=1 → VisionNear=0<br>If bsens01=2 & bsens04=missing & bsens03>1 → VisionNear=1<br><br>**VisionFar**<br>If bsens01=1 & bsens05=1 → Visionfar=0<br>If bsens01=2 & bsens06=1 → Visionfar=0<br>If bsens01=1 & bsens05>1 → Visionfar=1<br>If bsens01=2 & bsens06>1 → Visionfar=1<br>If bsens01=2 & bsens06=missing & bsens05=1 → Visionfar=0<br>If bsens01=2 & bsens06=missing & bsens05>1 → Visionfar=1<br><br>**Visionsub**<br>If bsense02= 1 or bsense02= 2 → Visionsub=0<br>If bsense02= 3 or bsense02= 4 → Visionsub=1	Variable name:<br>1.Visionnear<br>2.Visionfar<br>3.Visionsub<br><br>Variable label:<br>1.Do you see well up close?<br>2.Do you see well from a distance?<br>3.Do you rate your vision as bad? (subjective)<br><br>Value labels<br>0=no<br>1=yes
ActiFE Ulm	visual sense (see_BL)	0=good<br>1=bad	**Visionsub**<br>Use as is.<br>see_BL = Visionsub	

TILDA	Do you usually wear glasses or contact lenses? (ph101)<br><br><br>Do you usually wear ordinary glasses, bifocals or contact lenses? (ph101a)<br><br><br><br>Is your eyesight (using glasses or contact lens if you use them)... (ph102)<br>How good is your eyesight for seeing things at a distance, like recognising a friend across the street (using glasses or contact lens if you use them)? Would you say it is... (ph103)<br>How good is your eyesight for seeing things up close, like reading ordinary newspaper print (using glasses or contact lens if you use them)? Would you say it is... (ph104)	1=yes<br>5=no<br><br>1=glasses<br>2=bifocals<br>3=contact lenses<br><br>1=excellent<br>2=very good<br>3=good<br>4=fair<br>5=poor<br>6=registered or legally blind	**VisionNear**<br>If ph104<=3 → VisionNear=0<br>If ph104>=4 → VisionNear=1<br><br>**VisionFar**<br>If ph103<=3 → VisionFar=0<br>If ph103>=4 → VisionFar=1<br><br>**Visionsub**<br>If ph102 >=1 AND ph102<=3 → Visionsub=0<br>If ph102 >=4 → Visionsub=1	
B-PROOF	No data available		Not harmonized.	
Rotterdam Study	Visual acuity (measured vision)<br>From 15 February 2010 onwards, new variables for visual acuity are in use: v16816 and v16817. The previous variables for visual acuity v192 and v194 are not active anymore from this date.<br><br>Visual actuiy was measured using ETDRS charts.	0-1<br>8.88/9.99 = missing	Not harmonized.	
Vitamin B12 level (blood)				
LASA	**LASA C** (cmvitb12)<br>**LASA 3B:** no data?	Range in pMOL/L<br>-2= no data, n.a.<br>-1= no valid data	Use as is.<br>vitb12= b/cmvitb12	Variable name:<br>vitb12<br><br>Variable label:<br>Vitamin B12 (pmol/l)
ActiFE Ulm	No data available.		Not harmonized.	
TILDA	No data available.		Not harmonized.	
B-PROOF	VitaminB12	Range pmol/l	Use as is.<br>vitb12= VitaminB12	
Rotterdam	No data available		Not harmonized.	
Vitamin D level (blood)				
LASA	b/cmvitd25	Nmol/l	Use as is.<br>vitd= b/cmvitd25	Variable name:<br>vitd<br><br>Variable label:<br>Serum 25-hydroxy vitamin D (nmol/l)
ActiFE Ulm	Vitamin D (ng/ml) (LAB_Vitamin_D_BL)	Range in ng/ml:<br>2.0 – 67/7	Use as is.<br>vitd= 2.5 x LAB_Vitamin_D_BL	
TILDA	No data available.		Not harmonized.	
B-PROOF	vitaminD_25OH	Range nmol/l<br>-3= will be repeated<br>-2= below 4 nmol/l<br>-1= no determination	Use as is.<br>vitd= vitaminD_25OH	
Rotterdam	No data available		Not harmonized.	

Walking aid used during test				
LASA	Only data available for 3B:<br>Walking aid or wheelchair? (BMVAR709)<br><br>If yes:<br>-cane (BMV7091)<br>-walker (BMV7092)<br>-rollator (BMV7093)<br>-wheelchair (BMV7094)<br>-other (BMV7095)<br><br>Both LASA C and B:<br>Walking aids during walking test (C/BWALK06)	1=no<br>2=yes<br><br>0=not mentioned<br>1=mentioned<br><br>3B:<br>1=walking bar<br>2=stick<br>3=rollator<br>4=leaning on objects/interviewer<br>5=other<br>6=none<br><br>C:<br>1=walking bar<br>2=stick<br>3=other<br>4=none	**walkaid**<br>If BWALK06=6 → walkaid=0<br>If BWALK06>=1 and BWALK06<=3 walkaid=1<br><br>If CWALK06=4 → walkaid=0<br>If CWALK06=1 or CWALK06=2 walkaid=1<br><br>**walkingaidgen**<br>If BMVAR709 = 1 → walkingaidgen = 0<br>If BMVAR709 = 2 → walkingaidgen = 1	Variable name:<br>walkaid<br>walkingaidgen<br><br>Variable label:<br>Use of walking aid during walking test<br>Use of walking aid in general<br><br>Value label:<br>walkaid:<br>0=no<br>1=yes<br><br>walkingaidgen:<br>0=no<br>1=yes<br><br>Note: includes walking crunches, sticks, frames, bars, rollator, and wheelchair. Leaning on objects or on interviewer does not count as walking aid.<br><br>Note that walkingaidgen is missing for most participants in TILDA.
ActiFE Ulm	Walking aid (walking_aid_BL)<br><br><br><br><br>walking aid [SPPB] (SPPB_walkaid_BL)	1 = none<br>2 = walking aid<br>3 = crutches<br>4 = rollator<br><br>0=no<br>1=yes	**walkaid**<br>If SPPB_walkaid_BL = 0 → walkaid = 0<br>If SPPB_walkaid_BL = 1 → walkaid = 1<br><br>**walkingaidgen**<br>If walking_aid_BL = 1 → walkingaidgen = 0<br>If walking_aid_BL >= 2 AND walking_aid_BL <= 4 → walkingaidgen = 1	

TILDA	Do you ever use equipment or devices such as a walking stick or frame when crossing a room? (fl006)<br><br>Which equipment is that?<br>-walking stick (fl007_01) (public data)<br>-Walking frame (fl007_02) (public data)<br><br>Not in public data:<br>-Crutches (fl007_03)<br>-railing (fl007_04)<br>-orthopaedic shoes (fl007_05)<br>-brace (leg or back) (fl007_06)<br>-limb prosthesis (fl007_07)<br>-oxygen/respirator (fl007_08)<br>-Furniture or walls (fl007_09)<br>-wheelchair or cart (fl007_10)<br>-other (fl007_11)<br><br>During gait assessment: use of aid? (GRT_Used_walking_aid)	1=yes<br>5=no<br><br>0=no<br>1=yes<br><br>0=no<br>1=yes	**walkaid**<br>walkaid= GRT_Used_walking_aid<br><br>**walkingaidgen**<br>If fl006 = 5 → walkingaidgen = 0<br>If fl006 = 1 → walkingaidgen = 1	
B-PROOF	Do you use a walking aid? (walking_aid)<br><br>Type of first, second, third, fourth walking aid? (walking_aid_type1, walking_aid_type2, walking_aid_type3, walking_aid_type4)<br><br>Walking aid during walking test (pp6)	0=no<br>1=yes<br><br>1=stick<br>2=walking frame<br>3=rollator<br>4=wheelchair<br>5=other<br><br>1=walking frame<br>2=stick<br>3=other<br>4=no	**walkaid**<br>If pp6>=1 and pp6<=3 → walkaid=1<br>If pp6=4 → walkaid=0<br><br>**walkingaidgen**<br>If walking_aid = 0 → walkingaidgen = 0<br>If walking_aid = 1 → walkingaidgen = 1	

Rotterdam Study	Do you use any walking aids? (e5_EI3_50)<br><br>Loop capaciteit (e5_16407)	1 = none<br>2 = walking stick<br>3 = tripod<br>4 = walking frame without wheels<br>5 = walking frame with wheels<br>6 = crutches<br>7 = wheelchair<br>77 = don't know<br>99 = no answer<br><br>0=normal<br>1=c.norm, niet in tandem pos<br>2=zond.steun,abnorm.& onregel<br>3=z.st.,wagg.& moeilkh.draai<br>4=niet zonder autonome steun<br>5=alleen mogelijk met stok<br>6=alleen emt 2 stok.of rollat<br>7=alleen met begeleiding<br>8=lopen onmogelijk (rolstoel)<br>88=not applicable/default<br>99=missing	**walkaid**<br>if e5_16407=5 or e5_16407=6 or e5_16407=8 → walkaid=1<br>if e5_16407=0 or e5_16407=1 or e5_16407=2 or e5_16407=3 or e5_16407=4 or e5_16407=7 → walkaid=0<br><br>**walkingaidgen**<br>If e5_EI3_50 = 1 → walkingaidgen = 0<br>If e5_EI3_50 >= 2 AND e5_EI3_50 <= 7 → walkingaidgen = 1	
Waist circumference				
LASA	Waist circumference (cm) in duplicate (BMED156 BMED157; CMED156 CMED157)	Range 0-150	If CMED156/BMED156>0 and CMED157/BMED157>0 → waist=mean(CMED156/BMED156 CMED157/BMED157)<br>If CMED156/BMED156<0 or CMED157/BMED157<0 → waist= max(CMED156/BMED156, CMED157/BMED157)	Variable name:<br>waist<br><br>Variable label:<br>Waist circumference (cm)
ActiFE Ulm	No data available		Not harmonized.	
TILDA	Waist circumference (in cm) (FRwaist)	Range 49-163<br><br>N=2387 missing	Waist=FRwaist	
B-PROOF	No data available		Not harmonized.	
Rotterdam Study	Minimal waist circumference was measured in cm (e5_231)	Range<br>40.00-200.00<br>999.99=missing<br>888.88=not appropriate-def	Waist=e5_231<br>If e5_231 > 888 → waist=missing	
Weight				
LASA	Measured weight in kilograms (BMED153, CMED153)		weight = BMED153, CMED153	Variable name:<br>weight
ActiFE Ulm	body weight (kg) (weight_BL)		Use as is.<br>weight = weight_BL	

TILDA	Measured weight in kilograms (weight)<br><br>Note that self-reported weight is available for all waves	Range 45-135<br><br>NB: <=45 coded as 45;<br>135+ coded as 135<br>N=2374 missing	Use as is.	Variable label:<br>Weight in kg
B-PROOF	Weight of the participant in kg (1 decimal) (Weight)		Use as is.	
Rotterdam Study	Weight (in kg) (e5_230)	= 30.0 - 160.0<br>999.9 = missing<br>888.8 = not appropriate-default	Use as is.<br>If e5_230 > 888 → weight=missing	
Weight loss				
LASA	**LASA C & 3B:**<br>In the past 6 months: (c/bmvar142)<br><br>If 2 or 3; how much weight did you gain/loose? (mvar143)	1= weight remain stable<br>2= gain weight<br>3= loose weight<br><br>Range in kg (round up when decimals 0,5)	If c/bmvar142=1 or c/bmvar142=2 → weightloss=0<br>If c/bmvar142=3 → weightloss=1	Variable name:<br>weightloss<br><br>Variable label:<br>Weight loss?<br><br>Value label:<br>0= no<br>1= yes<br><br>Note: because of different time frames asked in the different studies, and different categories of weight loss (range or categories) we chose to create a simple weight loss (yes/no) variable.<br><br>Note: These questions all differ with respect to time frame and quantity of weight loss
ActiFE Ulm	In the past 3 months: weight loss (weight_loss_BL)	weight loss<br>0=none<br>1=1-3kg<br>2=>3kg<br>3=unknown	If weight_loss_BL = 0 → weightloss = 0<br>If weight_loss_BL = 1 or 2 → weightloss = 1	
TILDA	In the past year have you lost 10 pounds (4.5 kg) or more in weight when you weren't trying to, for example, because of illness? (ph008)	1= Yes<br>5= No<br>98= don't know<br>99= refused	If ph008=1 → weightloss=1<br>If ph008=5 → weightloss=0	
B-PROOF	No data available		Not harmonized.	
Rotterdam Study	Gewichtsverlies gedurende de periode (e5_4355)	0= geen verandering<br>1= matig verlies (niet 2)<br>2= meer dan 5% v.gew. in 1 mnd<br>3= meer dan 15% v.gew<br>4= als 3, niet aankomen onverw.*<br>8= not applicable/default**<br>9= missing<br><br>*) There are no cases in the data of e5_4355=4<br>**) There are 196 cases of e5_4355=8	If e5_4355=0 → weightloss=0<br>If e5_4355>=1 and e5_4355<=3 → weightloss=1	
Year of data collection				
LASA			LASA 3B: year = 2012<br>LASA C: year = 1995	Variable name:<br>year<br><br>Unit:<br>Year of data collection<br><br>Range 1989-2014
ActiFE Ulm	year of data collection (year_BL)		Use as is.<br>year = year_BL	
TILDA			year=2009	
B-PROOF	Date of interview (Date_interview)		year = xdate.year(Date_interview)	

Rotterdam Study	Date of interview (e5_3493)		year = xdate.year(e5_3493)	
**Medication use**				
**See Appendix 8.**				

Appendix 1

	LASA		TILDA		Rotterdam	B-PROOF	ActiFE Ulm
	3B (2012)	C (1995)	w1 (2010)	w3 (2014)	ERGO-5 (2009-2013)	Baseline (2008-2011)	Baseline (2009/2010)
**Arthritis**	1	1	1	1	1		1
**Depression**			1	1			1
**Diabetes**	1	1	1	1	1	1	1
**Heart disease**	1	1	1	1		1	1
**Hypertension**	1	1	1	1		1	1
**Stroke**	1	1	1	1	1	1	1
**Asthma**	1	1	1	1	1		1
**Bronchitis/emphysema**	1	1	1	1	1		1
**Osteoporosis**	1	1	1	1	1		1
**Cancer**	1	1	1	1	1		1
**Chronic fatigue syndrome**							
**Parkinson's disease**			1	1	1		

Derived variable '**number of chronic conditions**' includes diabetes, heart disease, lung disease and cancer (range 0-4)

Appendix 2

MET-values were based on overlap in categories between the cohorts and MET values used

	B-PROOF	LASA	ERGO	TILDA	ActiFE Ulm	harmonised
**walking**	3.5	3.5	3.5	3.3	3.5	
**cycling**	4.5	4.5	4.5		4.5	
**light household**	2.5	2.5	2.5		2.5	
**heavy household**	4.5	4.5	4.5		4.5	
**gardening**	4.5	4.5	4.5		4.5	
**sport/exercise**	4.0	4.0	4.0		4.0	
**moderate leisure[1]**				4.0		
**vigorous leisure[2]**				8.0		

[1] Including activities that make you breathe somewhat harder than normal, like carrying light loads, cycling at a regular pace or doubles tennis? Do not include walking.

[2] Including heavy lifting, digging, aerobics, fast cycling.

Note: *Vigorous/heavy* house and garden activities are included in the definition of physical activity, as these were counted in the responses to the IPAQ questions for moderate and vigorous activity used in TILDA.

Appendix 3

Definitions for levels of alcohol intake are typically based on guidelines for recommended alcohol intake. These guidelines are different for the four countries.

Definitions of low risk alcohol intake are:

- Australia: maximum of 2 drinks on any day (http://www.alcohol.gov.au/internet/alcohol/publishing.nsf/Content/guide-adult)
- Netherlands: maximum of 1 drink per day, both for men and women. (https://www.gezondheidsraad.nl/sites/default/files/201524_richtlijnen_goede_voeding_2015.pdf)
- Ireland: maximum of 17 drinks for men and 11 drinks for women, spread out over the course of a week with at least 2-3 alcohol free days (http://alcoholireland.ie/alcohol-and-you/guidelines/)
- UK: maximum of 14 drinks per week spread over 3 or more days (https://www.gov.uk/government/uploads/system/uploads/attachment_data/file/602132/Communicating_2016_CMO_guidelines_Mar_17.pdf)

I was unable to find global/international guidelines. The WHO reports on alcohol use, but does not specify a low risk level. In the Netherlands, the recommended intake was lowered to 1 per day in 2015. At the time the current data were collected (2012/13), the guidelines recommended a maximum of 2 glasses per day, which is in line with the guidelines in Australia and the UK. I therefore used that threshold to define the levels of risk as specified below and in line with the classification used in the Australian NHMRC guidelines. Researchers can decide to use alternative definitions if preferred.

	Quantity of alcohol consumed					
Frequency of drinking alcohol	I don't drink alcohol	1 or 2 drinks	3 or 4 drinks	5 to 8 drinks	9 or more drinks	Missing
I don't drink alcohol	0					0
I rarely drink		1	1	1	1	1
Less than once a week		2	2	2	2	
1 or 2 days		2	2	2	3	
3 or 4 days		2	2	3	4	
5 or 6 days		2	3	4	4	
Every day		2	3	4	4	
Missing	0	2				

0=non-drinker
1=rarely drinks
2=low risk (≤14 per week)
3=risky (15-28 per week)
4=high risk (>28 per week)

Appendix 4

Overview of items included in each of the anxiety scales

	Scales				
ITEMS	Goldberg	HADS	GAD	PSF	GHQ
feeling tense, keyed up or on edge	1	1	1	1	1
worrying	1	1	1	1	
irritable	1		1		1
difficulty relaxing	1	1			1
sleeping poorly	1			1	
difficulty falling asleep	1			1	1
headaches or neck aches	1				
trembling, tingling, dizzy, sweating, diarrhoea, passing urine	1			1	
frightened		1			
restless		1	1	1	
sudden panics				1	1
feeling tired			1	1	
difficulty keeping mind on what you are doing			1	1	
tense, sore or aching muscles			1		
feeling something terrible might happen				1	
couldn't do anything because nerves were too bad					1

Appendix 5

Overview of cardiovascular diseases measured in the cohorts

	**LASA**		**TILDA**	**B-PROOF**	**Rotterdam study**	**ActiFE Ulm**
	C	3B	Wave 1; public data	Baseline wave	ERGO-5	Baseline wave
Arrhythmia	X	X	X	X		
Angina Pectoris	X	X	X	X		
Myocardial infarction	X	X	X	X	X	x
Heart failure	X	X	X	X		x
Stroke/CVA	X	X	X	X (includes TIA)	X	x
Peripheral arterial disease (PAD)	X	X				

Appendix 6

Overview of items mentioned for functional limitations

ITEMS	LASA-C	LASA-3B	Tilda	B-PROOF	ERGO-5	ActiFE Ulm
Get clothes from closets or drawers					X	
**Dress and/or undress**	X	X	X	Combi with wash	X	X
Wash hair					X	
**Get up from chair**	X	X	X		Without arms	X
Get out of bed			X		X	
Eating, cutting food, drinking			X		X	
Open new carton of milk					X	
**Walk (outside)**	5 minutes	5 minutes / In general (Euroqol)	100 meter	In general (Euroqol)	X	5 minutes
**Walk stairs without rest**	15 steps	15 steps	1 flight of stairs	In general	5 steps	15 steps
Was & dry entire body				Combi with dress	X	
Take bath or use shower		X	X		X	X
Open and close tap					X	
Sit down and get up from toilet			X		X	
Comb & style hair					X	
Grab something from shelf above head			Arms above shoulder		X	
**Bend down to pick something from floor**			Stoop/kneel/crouch		X	
Open car door from outside					X	
Open jar of jam that has been opened before					X	
Use a pen or pencil					X	
Daily grocery shopping					X	X
Get in and out of passenger car					X	
Travel independently/use own or public transport	X	X			X	X
Do small chores in house					X	X
Run the household					X	
Cycle on your own					X	
Using telephone					X	
Cooking a dish					X	
Doing the laundry					X	
Taking medication					X	X
Arranging finance					X	
**Cut toenails**	X	X				X
Daily activities in general		X (Euroqol)		X (Euroqol)		X (Euroqol)

Appendix 7

Overview of relevant genetic variants

		LASA		B-PROOF	Rotterdam study
		**C**	**3B**	**Baseline wave**	**ERGO-5**
**CYP2C19**	rs4244285				
	rs12248560				
**CYP3A4**	rs35599367				
**CYP3A5**	rs776746				
**CYP2D6**	rs3892097				
**CYP2C9**	rs1799853				
	rs1057910				
**CYP1A2**	rs2069514				
	rs762551				
	rs12720461				
**SLCO1B1**	rs4149056				
**ABCB1**	rs1045642				
	rs2032582				
	rs1128503				
**CYP2C8**	rs1058930				
	rs10509681				
	rs11572080				
**CYP1A1**	rs4646903				
	rs1048943				

Appendix 8. Medication classes and ATC codes belonging to these groups

**Medication classes**	**ATC codes belonging to this group**
Proton pump inhibitor	A02BC<br>A02BD01<br>A02BD02<br>A02BD03<br>A02BD04<br>A02BD05<br>A02BD06<br>A02BD07<br>A02BD09<br>A02BD10<br>A02BD11<br>B01AC56<br>M01AE52
blood glucose lowering drugs, excl. insulins	A10B
Insulins and analogues	A10A
Cardiac glycosides	C01A
Antiarrhythmics, class I and III	C01B
Vasodilators used in cardiac diseases	C01DA

Antiadrenergic agents, antihypertensives	C02A<br>C02B<br>C02C<br>C02LA<br>C02LB<br>C02LC<br>C02LE<br>C02LF
Diuretic use	C03<br>C02L<br>C07B<br>C07C<br>C07D<br>C08GA<br>C09BA<br>C09DA<br>C09DX01<br>C09DX03<br>C09DX06<br>C09AX54<br>C09AX52<br>C09BX03<br>C09BX01<br>C10BX13
High-ceiling diuretics	C03C<br>C03EB
Low-ceiling diuretics	C03A<br>C03B<br>C03EA<br>C07B<br>C07C<br>C07D<br>C08GA<br>C09BA<br>C09DA<br>C09DX01<br>C09DX03<br>C09DX06<br>C09AX54<br>C09AX52<br>C09BX03<br>C09BX01<br>C10BX13<br>C02L
Non-selective beta-blocker use	C07AA<br>C07AG<br>C07BA<br>C07BG<br>C07CA<br>C07CG<br>C07DA<br>C07EA<br>C07FX01<br>C07FX02<br>C07FX06<br>C07FA05

Selective beta blocker use	C07AB<br>C07BB<br>C07CB<br>C07DB<br>C07EB<br>C07FB02<br>C07FB03<br>C07FB07<br>C07FB12<br>C07FB13<br>C07FX03<br>C07FX04<br>C07FX05<br>C09BX02
Calcium channel blockers	C08<br>C09BB<br>C09DB<br>C07FB<br>C10BX03<br>C10BX07<br>C10BX09<br>C10BX11<br>C10BX14<br>C09BX03<br>C09BX01<br>C09BX02<br>C09DX01<br>C09DX03<br>C09DX06<br>C09XA53<br>C09XA54
ACE inhibitors	C09A<br>C09B<br>C10BX04<br>C10BX06<br>C10BX07<br>C10BX11<br>C10BX12<br>C10BX13<br>C10BX14<br>C10BX15
Angiotensin II Antagonist	C09C<br>C09D<br>C10BX10
Statins	C10AA<br>C10BA<br>C10BX<br>A10BH51<br>A10BH52
Drugs for urinary frequency and incontinence	G04BD<br>G04CA53
Alpha-adrenoreceptor antagonist used in benign prostatic hypertrophy	G04CA
NSAID	M01AA<br>M01AB<br>M01AC<br>M01AE

	M01AG<br>M01AH<br>M01AX01<br>M01AX02<br>M01AX04<br>M01AX07<br>M01AX13<br>M01AX17<br>M01AX18<br>M01AX22<br>M01AX23<br>M01AX68<br>M01BA02<br>N02AJ08<br>N02AJ14<br>N02AJ19
Opioids	N02A<br>N02BE51<br>N02BA51<br>M01AE51
Antiepileptics	N03
Anti-parkinson drugs	N04
Antipsychotics	N05A
Non-selective monoamine reuptake inhibitors	N06AA<br>N06CA01<br>N06CA02
SSRIs	N06AB<br>N06CA03
Other Antidepressant	N06AX<br>N06AF<br>N06AG
Antihistamines	R06
Benzodiazepines	N05BA, N05CD
Benzodiazepine-related drugs	N05CF
Anticholinergic medications<br>(only includes medicines with ACB score of 3)<br>Based on the Anticholinergic cognitive burden scale:<br>http://www.miltonkeynesccg.nhs.uk/resources/uploads/ACB_scale_-_legal_size.pdf	A03AA07, A03AB05, A03BA01, A03BA03, A03BB01, G04BD02, G04BD04, G04BD05, G04BD06, G04BD07, G04BD08, G04BD09, G04BD10, G04BD11, M03BA03, M03BC01, N04AA01, N04AA02, N04AA04, N04AB02, N04AC01, N05AA01, N05AA03, N05AB03, N05AB06, N05AC02, N05AH03, N05AH04, N05BB01, N05CM05, N06AA, N06AB05, R06AA02, R06AA04, R06AA08, R06AA09, R06AA52, R06AB01, R06AB04, R06AD02, R06AE05
Supplements that were excluded from the *number of medications* variable:	A11 (all underlying ATC-codes), A12 , A13, B03AA, B03AB, B03AD, B03AE,

This list is based on Directive 2002/46/EC of the European Parliament.	B03B, B02BA, C10AX06, M01AX05, N06DX02, V06, homeopathic preparations, and herbal supplements

Supplementary Table 1. Complete characteristics of the cohort studies with prospective data on falls

Variable	LASA (*n* = 1433)	B-PROOF (*n* = 2912)	ActiFE Ulm (*n* = 1377)	Total (*n* = 5722)
Outcome variables				
Any fall in follow-up (≥ 1 falls)	468 (33.9)	957 (35.0)	443 (35.1)	1868 (34.7)
Recurrent falls in follow-up (≥ 2 falls)	174 (12.7)	375 (13.8)	162 (13)	711 (13.3)
Sociodemographic variables				
Age (years)	75 [69, 81]	73 [69, 78]	74 [70, 81]	74 [69, 79]
Sex, female	738 (51.5)	1456 (50.0)	591 (42.9)	2785 (48.7)
Educational status				
Low	1038 (72.5)	2006 (68.9)	1069 (78.5)	4113 (72.1)
Middle	219 (15.3)	149 (5.1)	141 (10.4)	509 (8.9)
High	174 (12.2)	755 (25.9)	151 (11.1)	1080 (18.9)
Living situation (institutionalized)	72 (5.0)	36 (3.2)	0 (0.0)	108 (2.7)
Living with partner	779 (54.4)	1839 (63.2)	908 (66.3)	3526 (61.7)
Measures of emotional functioning				
Depressive symptoms[a,b]	212 (15.3)	132 (4.6)	140 (10.7)	484 (8.7)
HADS-A score	2 [0, 4]	—	4 [2, 6]	3 [1, 5]
Measures of cognitive functioning				
Immediate recall (number of words)	11.04 ± 3.71	10.77 ± 3.26	—	10.86 ± 3.42
Delayed recall (number of words)	6 [3, 8]	7 [5, 9]	—	6 [4, 9]
MMSE score	28 [26, 29]	28 [27, 29]	29 [27, 30]	28 [27, 29]
Verbal fluency[b]				
Animals named in test	—	—	21 [17, 25]	21 [17, 25]
Items named in test	—	12 [9, 15]	—	12 [9, 15]
Processing speed[b]				
Score on adjusted Alphabet Coding task-15	23 [18, 28]	—	—	23 [18, 28]
Time spent in seconds on Trail Making Test	—	68 [53.50, 87]	—	68 [53.50, 87]
Hearing impairment	308 (21.5)	—	334 (24.7)	642 (23.0)
Visual impairment	102 (7.1)	—	201 (14.8)	303 (10.9)

Symptoms of dizziness	216 (15.1)	—	312 (22.9)	528 (18.9)
Measures of physical functioning				
BMI	26.93 ± 4.28	27.14 ± 3.96	27.56 ± 4.12	27.19 ± 4.08
Weight loss in past 3-6 months	217 (15.2)	—	150 (11.1)	367 (13.2)
Use of walking aid	—	422 (14.6)	15 (1.2)	437 (10.5)
Able to perform tandem stand for 10s	885 (64.5)	1960 (67.5)	1171 (89.6)	4016 (72.0)
Symptoms of pain	370 (31.0)	1347 (46.3)	812 (59.3)	2529 (46.2)
Poor self-rated health	196 (13.7)	380 (13.1)	213 (15.5)	789 (13.8)
Number of functional limitations (0-5)[b]	1 [0, 2]	—	0 [0, 2]	1 [0, 2]
Grip strength (kg)[b]	29.23 ± 10.18	32.49 ± 10.84	33.51 ± 11.37	31.91 ± 10.93
Gait speed (m/s)[b]	0.79 ± 0.27	0.94 ± 0.28	1.04 ± 0.32	0.93 ± 0.30
Urinary incontinence	359 (25.1)	—	511 (37.6)	870 (31.2)
Systolic blood pressure (mmHg)	153.08 ± 23.91	148.73 ± 19.68	142.34 ± 11.81	148.20 ± 19.67
Diastolic blood pressure (mmHg)	83.53 ± 12.16	80.16 ± 12.82	79.51 ± 10.67	80.90 ± 12.20
Pulse rate (beats/min)	69.25 ± 11.60	68.67 ± 13.38	68.61 ± 12.25	68.90 ± 12.12
≥ 1 fall in previous 12 months	458 (32.0)	737 (32.6)	469 (34.5)	1664 (33.0)
≥ 2 falls in previous 12 months	215 (15.1)	268 (11.9)	157 (11.6)	640 (12.7)
Fear of falling				
Not afraid of falling	667 (46.8)	—	829 (60.7)	1496 (53.6)
Somewhat afraid of falling	567 (39.8)	—	489 (35.8)	1056 (37.8)
Very afraid of falling	191 (13.4)	—	47 (3.4)	238 (8.5)
Self-reported chronic conditions				
Cancer ever	183 (12.8)	—	261 (19.0)	444 (15.8)
Diabetes ever	114 (8.0)	233 (10.3)	180 (13.1)	527 (10.4)
Any cardiovascular disease[c]	394 (28.5)	893 (30.7)	568 (41.3)	1855 (32.4)
Heart failure ever	79 (5.6)	85 (3.8)	202 (14.7)	366 (7.3)
Angina pectoris ever	162 (11.4)	61 (2.7)	—	223 (6.1)
Arrhythmia ever	171 (12.2)	183 (8.2)	—	354 (9.7)
Myocardial infarction ever	38 (2.7)	215 (9.6)	124 (9.0)	377 (7.5)
Stroke ever	115 (8.0)	198 (8.8)	73 (5.3)	386 (7.6)
Lung disease ever	230 (16.1)	—	104 (7.6)	334 (12.0)

Arthritis ever	705 (49.2)	—	675 (49.2)	1380 (49.2)
Comorbidity[c]	964 (67.3)	1994 (68.5)	976 (70.9)	3934 (68.8)
Variables related to lifestyle				
Total physical activity (MET/week)[b]	2968.75 [1630.94, 4585.62]	3211.25 [2047.00, 4883.12]	5131.00 [3433.50, 7336.00]	3569.38 [2190.00, 5470.50]
Alcohol use				
Non-drinker	358 (25.0)	399 (13.7)	10 (0.7)	767 (13.5)
Drinks less than once a month	161 (11.2)	270 (9.3)	221 (16.3)	652 (11.4)
Drinks 1-3 times a month	175 (12.2)	305 (10.5)	345 (25.5)	825 (14.5)
Drinks 1-4 days a week	343 (24.0)	808 (27.8)	354 (26.2)	1505 (26.4)
Drinks (almost) daily	395 (27.6)	1129 (38.8)	423 (31.3)	1947 (34.2)
Current smoker	265 (18.5)	281 (9.6)	88 (6.4)	634 (11.1)
Biomarkers				
eGFR (mL/min)	61.13 ± 17.67	72.48 ± 20.64	65.99 ± 19.09	68.25 ± 20.17
CRP (mg/l)	6.72 ± 13.52	2.89 ± 5.01	3.35 ± 5.82	3.88 ± 8.10
Vitamin B12 (pmol/l)	338.48 ± 578.04	285.43 ± 115.94	—	301.45 ± 332.91
Vitamin D (nmol/l)	53.44 ± 24.04	55.70 ± 24.83	51.10 ± 18.52	54.04 ± 23.31
Medication use				
Number of medications	2 [0, 3]	3 [1, 5]	3 [1, 5]	3 [1, 5]
Cardiovascular drugs				
Antiarrhythmics	18 (1.3)	53 (1.8)	24 (1.7)	95 (1.7)
Cardiac glycosides	95 (6.6)	64 (2.2)	40 (2.9)	199 (3.5)
Nitrates	132 (9.2)	122 (4.2)	22 (1.6)	276 (4.8)
Antiadrenergic agents	26 (1.8)	29 (1.0)	29 (2.1)	84 (1.5)
Diuretics	316 (22.1)	771 (26.5)	214 (15.5)	1301 (22.7)
Low-ceiling diuretics	171 (11.9)	617 (21.2)	103 (7.5)	891 (15.6)
High-ceiling diuretics	133 (9.3)	146 (5.0)	115 (8.4)	394 (6.9)
Other diuretics	28 (2.0)	120 (4.1)	20 (1.5)	168 (2.9)
Beta-blocking agents	231 (16.1)	751 (25.8)	424 (30.8)	1406 (24.6)

Non-selective beta-blocking agents	73 (5.1)	106 (3.6)	41 (3.0)	220 (3.8)
Selective beta-blocking agents	158 (11.0)	649 (22.3)	383 (27.8)	1190 (20.8)
Calcium channel blockers	103 (7.2)	376 (12.9)	220 (16.0)	699 (12.2)
ACE inhibitors	156 (10.9)	475 (16.3)	246 (17.9)	877 (15.3)
Angiotensin II antagonists	0 (0.0)	507 (17.4)	122 (8.9)	629 (11.0)
Alpha-adrenoceptor antagonists	10 (0.7)	152 (5.2)	133 (9.7)	295 (5.2)
Statins	62 (4.3)	724 (24.9)	362 (26.3)	1148 (20.1)
Psychotropics				
Antipsychotics	22 (1.5)	19 (0.7)	14 (1.0)	55 (1.0)
Antidepressants	35 (2.4)	145 (5.0)	70 (5.1)	250 (4.4)
SSRIs	9 (0.6)	78 (2.7)	24 (1.7)	111 (1.9)
TCAs	25 (1.7)	40 (1.4)	35 (2.5)	100 (1.7)
Other antidepressants	1 (0.1)	28 (1.0)	15 (1.1)	44 (0.8)
Benzodiazepines	200 (14.0)	150 (5.2)	15 (1.1)	365 (6.4)
Benzodiazepine-related drugs	9 (0.6)	39 (1.3)	13 (0.9)	61 (1.1)
Others medications				
Anti-Parkinson drugs	6 (0.4)	38 (1.3)	24 (1.7)	68 (1.2)
Antiepileptics	17 (1.2)	52 (1.8)	39 (2.8)	108 (1.9)
Opioids	24 (1.7)	101 (3.5)	18 (1.3)	143 (2.5)
NSAIDs	142 (9.9)	164 (5.6)	143 (10.4)	449 (7.8)
Anticholinergic medications	40 (2.8)	127 (4.4)	67 (4.9)	234 (4.1)
Insulin	30 (2.1)	42 (1.4)	29 (2.1)	101 (1.8)
Other glucose lowering drugs	57 (4.0)	240 (8.2)	121 (8.8)	418 (7.3)
Proton pump inhibitors	37 (2.6)	612 (21.0)	183 (13.3)	832 (14.5)
Drugs for urinary frequency and incontinence	7 (0.5)	40 (1.4)	29 (2.1)	76 (1.3)
Antihistamines	16 (1.1)	83 (2.9)	17 (1.2)	116 (2.0)

Data are presented as mean ± SD, n (%), or median [IQR]. The sign "—" indicates the corresponding variable is systematically missing.

BMI, body mass index; HADS-A, Hospital Anxiety Depression Scale-Anxiety subscale; MMSE, Mini-Mental State Examination; MET, Metabolic Equivalent of Task; eGFR, estimated glomerular filtration rate; CRP, C-reactive protein; ACE, angiotensin-converting enzyme; SSRI, selective serotonin reuptake inhibitor; TCA, tricyclic antidepressant; NSAID, nonsteroidal anti-inflammatory drug

[a]Defined by validated cutoff scores for Center for Epidemiological Studies Depression Scale (in LASA), Hospital Anxiety and Depression Scale-Depression subscale (in ActiFE Ulm), and Geriatric Depression Scale (in B-PROOF)

[b]Variable was harmonized across the three cohort studies and modelled using z-scores

[c]Variable was computed after multiple imputation

Supplementary Table 2. Complete characteristics of the cohort studies with retrospective data on falls

	Cohorts with retrospective and prospective data on falls			Cohorts with retrospective data on falls			
Variable	LASA: wave C (*n* = 1507)	B-PROOF (*n* = 2912)	ActiFE Ulm (*n* = 1463)	LASA: wave 3B (*n* = 887)	Rotterdam Study (*n* = 7151)	TILDA (*n* = 8081)	Total (*n* = 22001)
Sociodemographic variables							
Age (years)	75 [70, 81]	73 [69, 78]	74 [70, 81]	60 [57, 62.50]	69 [63, 77]	62 [56, 71]	68 [61, 76]
Sex, female	781 (51.8)	1456 (50.0)	634 (43.3)	454 (51.2)	4163 (58.2)	4375 (54.1)	11863 (53.9)
Educational status							
Low	1094 (72.7)	2006 (68.9)	1137 (78.7)	374 (42.2)	3544 (50.1)	4341 (53.7)	12496 (57.1)
Middle	231 (15.3)	149 (5.1)	147 (10.2)	229 (25.8)	2086 (29.5)	1351 (16.7)	4193 (19.1)
High	180 (12.0)	755 (25.9)	160 (11.1)	284 (32.0)	1449 (20.5)	2386 (29.5)	5214 (23.8)
Living situation (institutionalized)	88 (5.8)	36 (3.2)	0 (0.0)	2 (0.2)	702 (9.9)	0 (0.0)	828 (4.1)
Living with partner	806 (53.5)	1839 (63.2)	951 (65.4)	658 (74.2)	4834 (67.7)	5592 (69.2)	14680 (66.8)
Measures of emotional functioning							
Depressive symptoms[a,b]	229 (15.8)	132 (4.6)	154 (11.2)	106 (12.0)	656 (9.8)	763 (9.6)	2040 (9.6)
HADS-A score	2 [0, 4]	—	4 [2, 6]	2 [0, 4]	2 [0, 4]	5 [3, 7]	3 [1, 6]
Measures of cognitive functioning							
Immediate recall (number of words)	10.94 ± 3.75	10.77 ± 3.26	—	13.18 ± 3.34	13.07 ± 3.71	13.13 ± 3.30	12.59 ± 3.59

Delayed recall (number of words)	5 [3, 8]	7 [5, 9]	—	7 [5, 9]	8 [5, 10]	6 [4, 8]	7 [5, 9]
MMSE score	27 [26, 29]	28 [27, 29]	29 [27, 29]	29 [27, 29]	25 [23, 27]	29 [28, 30]	29 [27, 30]
Verbal fluency[b]							
Animals named in test	—	—	21 [17, 25]	22 [19, 26]	—	—	21 [18, 26]
Items named in test	—	12 [9, 15]	—	—	22 [18, 26]	20 [15, 25]	20 [16, 25]
Processing speed[b]							
Score on adjusted Alphabet Coding task-15	23 [17, 28]	—	—	31 [26, 34]	—	—	26 [20, 31]
Time spent in seconds on Trail Making Test	—	68 [53.50, 87]	—	—	89 [78, 104]	77 [63, 100]	84 [70, 102]
Hearing impairment	330 (21.9)	—	358 (25.0)	66 (7.4)	702 (14.6)	1145 (14.2)	2601 (15.6)
Visual impairment	110 (7.3)	—	216 (15.1)	28 (3.2)	—	798 (9.9)	1152 (9.7)
Symptoms of dizziness	235 (15.6)	—	335 (23.2)	58 (6.5)	1126 (23.4)	—	1754 (20.3)
Measures of physical functioning							
BMI	26.90 ± 4.30	27.14 ± 3.96	27.59 ± 4.14	27.15 ± 4.64	27.56 ± 4.39	28.51 ± 4.62	27.72 ± 4.42
Weight loss in past 3-6 months	234 (15.6)	—	164 (11.5)	155 (17.6)	—	599 (7.4)	1152 (9.7)
Use of walking aid	—	422 (14.6)	19 (1.4)	29 (3.3)	531 (7.5)	69 (71.1)	1070 (8.7)
Able to perform tandem stand for 10s	911 (63.4)	1960 (67.5)	1227 (88.5)	820 (92.8)	3411 (93.4)	—	8329 (81.2)
Symptoms of pain	385 (31.3)	1347 (46.3)	864 (59.4)	227 (27.9)	1656 (54.9)	2859 (35.4)	7338 (41.9)

Poor self-rated health	204 (13.5)	380 (13.1)	241 (16.6)	112 (12.6)	—	1866 (23.1)	2803 (18.9)
Number of functional limitations (0-5)[b]	1 [0, 2]	—	0 [0, 2]	0 [0, 1]	1 [0, 3]	0 [0, 1]	0 [0, 1]
Grip strength (kg)[b]	29.00 ± 10.24	32.49 ± 10.84	33.44 ± 11.41	35.52 ± 12.30	28.03 ± 10.33	28.08 ± 10.04	29.59 ± 10.77
Gait speed (m/s)[b]	0.79 ± 0.27	0.94 ± 0.28	1.03 ± 0.33	1.04 ± 0.24	1.20 ± 0.20	1.36 ± 0.21	1.14 ± 0.30
Urinary incontinence	386 (25.6)	—	541 (37.6)	151 (17.0)		1014 (12.6)	2092 (17.6)
Systolic blood pressure (mmHg)	153.21 ± 24.17	148.73 ± 19.68	142.30 ± 11.75	137.57 ± 19.11	143.93 ± 22.16	135.68 ± 19.91	142.22 ± 21.24
Diastolic blood pressure (mmHg)	83.48 ± 12.19	80.16 ± 12.82	79.57 ± 10.69	83.78 ± 10.97	83.48 ± 11.17	82.36 ± 11.29	82.39 ± 11.56
Pulse rate (beats/min)	69.39 ± 11.64	68.67 ± 13.38	68.75 ± 12.45	70.59 ± 11.82	69.52 ± 11.31	—	69.45 ± 11.66
≥ 1 fall in previous 12 months	485 (32.3)	737 (32.6)	502 (34.9)	222 (25.1)	1690 (23.7)	1566 (19.4)	5202 (24.4)
≥ 2 falls in previous 12 months	230 (15.3)	268 (11.9)	171 (11.9)	79 (8.9)	—	577 (7.2)	1325 (9.4)
Fear of falling							
Not afraid of falling	693 (46.3)	—	868 (60.1)	—	—	6209 (76.9)	7770 (70.6)
Somewhat afraid of falling	598 (40.0)	—	520 (36.0)	—	—	1404 (17.4)	2522 (22.9)
Very afraid of falling	205 (13.7)	—	57 (3.9)	—	—	459 (5.7)	721 (6.5)
Self-reported chronic conditions							
Cancer ever	188 (12.5)	—	273 (18.7)	85 (9.6)	823 (20.4)	512 (6.3)	1881 (11.8)
Diabetes ever	125 (8.3)	233 (10.3)	198 (13.6)	69 (7.8)	867 (12.2)	621 (7.7)	2113 (9.9)

Any cardiovascular disease[c]	419 (27.8)	865 (29.7)	610 (41.7)	88 (9.9)	2220 (31.0)	1208 (14.9)	5410 (24.6)
Heart failure ever	83 (5.6)	85 (3.8)	214 (14.7)	10 (1.1)	—	87 (1.1)	479 (3.4)
Angina pectoris ever	169 (11.3)	61 (2.7)	—	26 (2.9)	—	442 (5.5)	698 (5.5)
Arrhythmia ever	183 (12.4)	183 (8.2)	—	26 (3.0)	—	580 (7.2)	972 (7.7)
Myocardial infarction ever	39 (2.6)	215 (9.6)	131 (9.0)	31 (3.5)	452 (6.3)	376 (4.7)	1244 (5.8)
Stroke ever	123 (8.2)	198 (8.8)	79 (5.4)	21 (2.4)	68 (1.9)	131 (1.6)	620 (3.5)
Lung disease ever	241 (16.0)	—	113 (7.8)	90 (10.1)	175 (7.0)	977 (12.1)	1596 (11.1)
Arthritis ever	744 (49.4)	—	724 (49.6)	362 (40.8)	3034 (44.0)	2223 (27.5)	7087 (37.6)
Comorbidity[c]	1024 (67.9)	1909 (65.5)	1054 (72.0)	391 (44.0)	4546 (63.6)	3635 (45.0)	12558 (57.1)
Variables related to lifestyle							
Total physical activity (MET/week)[b]	2910.00 [1575.00, 4567.50]	3211.25 [2047.00, 4883.12]	5082.00 [3389.75, 7320.25]	3405.00 [2026.50, 5055.00]	3060.00 [1710.00, 4935.00]	1653.00 [587.25, 4158.00]	2790.00 [1266.75, 4914.00]
Alcohol use							
Non-drinker	387 (25.7)	399 (13.7)	11 (0.8)	63 (7.1)	1183 (16.6)	1878 (27.9)	3921 (19.0)
Drinks less than once a month	169 (11.2)	270 (9.3)	240 (16.7)	61 (6.9)	1111 (15.6)	625 (9.3)	2476 (12.0)
Drinks 1-3 times a month	184 (12.2)	305 (10.5)	362 (25.3)	115 (13.0)	898 (12.6)	818 (12.1)	2682 (13.0)
Drinks 1-4 days a week	359 (23.8)	808 (27.8)	375 (26.2)	358 (40.4)	1104 (15.5)	2728 (40.5)	5732 (27.8)

Drinks (almost) daily	407 (27.0)	1129 (38.8)	445 (31.1)	290 (32.7)	2826 (39.7)	685 (10.2)	5782 (28.1)
Current smoker	287 (19.1)	281 (9.6)	100 (6.9)	159 (18.0)	897 (12.6)	1468 (18.2)	3192 (14.5)
Biomarkers							
eGFR (mL/min)	60.98 ± 17.63	72.48 ± 20.64	65.93 ± 19.09	94.49 ± 23.40	82.03 ± 25.48	—	76.28 ± 24.41
CRP (mg/l)	6.83 ± 13.64	2.89 ± 5.01	3.40 ± 6.17	2.65 ± 4.96	—	3.34 ± 9.84	3.58 ± 8.89
Vitamin B12 (pmol/l)	337.30 ± 571.99	285.43 ± 115.94	—	—	—	—	301.34 ± 331.94
Vitamin D (nmol/l)	53.21 ± 24.00	55.70 ± 24.83	51.14 ± 18.84	68.66 ± 21.98	—	—	55.44 ± 23.60
Medication use							
Number of medications	2 [1, 3]	3 [1, 5]	3 [1, 5]	1 [0, 3]	3 [1, 5]	2 [0, 4]	2 [1, 4]
Cardiovascular drugs							
Antiarrhythmics	18 (1.2)	53 (1.8)	24 (1.6)	8 (0.9)	173 (2.4)	37 (0.5)	313 (1.4)
Cardiac glycosides	104 (6.9)	64 (2.2)	40 (2.7)	3 (0.3)	113 (1.6)	99 (1.2)	423 (1.9)
Nitrates	138 (9.2)	122 (4.2)	24 (1.6)	8 (0.9)	400 (5.6)	137 (1.7)	829 (3.8)
Antiadrenergic agents	28 (1.9)	29 (1.0)	34 (2.3)	3 (0.3)	43 (0.6)	153 (1.9)	290 (1.3)
Diuretics	347 (23.0)	771 (26.5)	235 (16.1)	112 (12.6)	1678 (23.5)	1102 (13.6)	4245 (19.3)
Low-ceiling diuretics	190 (12.6)	617 (21.2)	113 (7.7)	103 (11.6)	1251 (17.5)	787 (9.7)	3061 (13.9)
High-ceiling diuretics	144 (9.6)	146 (5.0)	129 (8.8)	11 (1.2)	415 (5.8)	326 (4.0)	1171 (5.3)
Other diuretics	30 (2.0)	120 (4.1)	24 (1.6)	3 (0.3)	130 (1.8)	15 (0.2)	322 (1.5)
Beta-blocking agents	237 (15.7)	751 (25.8)	449 (30.7)	132 (14.9)	1812 (25.3)	1164 (14.4)	4545 (20.7)

Non-selective beta-blocking agents	75 (5.0)	106 (3.6)	43 (2.9)	13 (1.5)	174 (2.4)	71 (0.9)	482 (2.2)
Selective beta-blocking agents	162 (10.7)	649 (22.3)	406 (27.8)	119 (13.4)	1638 (22.9)	1097 (13.6)	4071 (18.5)
Calcium channel blockers	110 (7.3)	376 (12.9)	237 (16.2)	66 (7.4)	639 (8.9)	863 (10.7)	2291 (10.4)
ACE inhibitor	165 (10.9)	475 (16.3)	264 (18.0)	86 (9.7)	1077 (15.1)	1099 (13.6)	3166 (14.4)
Angiotensin II antagonists	0 (0.0)	507 (17.4)	133 (9.1)	73 (8.2)	979 (13.7)	960 (11.9)	2652 (12.1)
Alpha-adrenoceptor antagonists	11 (0.7)	152 (5.2)	143 (9.8)	13 (1.5)	264 (3.7)	169 (2.1)	752 (3.4)
Statins	62 (4.1)	724 (24.9)	386 (26.4)	150 (16.9)	1957 (27.4)	2437 (30.2)	5716 (26.0)
Psychotropics							
Antipsychotics	24 (1.6)	19 (0.7)	16 (1.1)	8 (0.9)	48 (0.7)	111 (1.4)	226 (1.0)
Antidepressants	40 (2.7)	145 (5.0)	75 (5.1)	64 (7.2)	488 (6.8)	560 (6.9)	1372 (6.2)
SSRIs	10 (0.7)	78 (2.7)	25 (1.7)	40 (4.5)	265 (3.7)	306 (3.8)	724 (3.3)
TCAs	28 (1.9)	40 (1.4)	37 (2.5)	11 (1.2)	131 (1.8)	116 (1.4)	363 (1.6)
Other antidepressants	2 (0.1)	28 (1.0)	17 (1.2)	17 (1.9)	101 (1.4)	157 (1.9)	322 (1.5)
Benzodiazepines	215 (14.3)	150 (5.2)	16 (1.1)	32 (3.6)	583 (8.2)	301 (3.7)	1297 (5.9)
Benzodiazepine-related drugs	10 (0.7)	39 (1.3)	13 (0.9)	9 (1.0)	115 (1.6)	203 (2.5)	389 (1.8)
Others medications							
Anti-Parkinson drugs	7 (0.5)	38 (1.3)	25 (1.7)	6 (0.7)	58 (0.8)	57 (0.7)	191 (0.9)

Antiepileptics	18 (1.2)	52 (1.8)	42 (2.9)	17 (1.9)	158 (2.2)	234 (2.9)	521 (2.4)
Opioids	25 (1.7)	101 (3.5)	18 (1.2)	30 (3.4)	328 (4.6)	370 (4.6)	872 (4.0)
NSAIDs	148 (9.8)	164 (5.6)	155 (10.6)	47 (5.3)	487 (6.8)	525 (6.5)	1526 (6.9)
Anticholinergic medications	47 (3.1)	127 (4.4)	72 (4.9)	34 (3.8)	395 (5.5)	338 (4.2)	1013 (4.6)
Insulin	31 (2.1)	42 (1.4)	31 (2.1)	16 (1.8)	175 (2.4)	69 (0.9)	364 (1.7)
Other glucose lowering drugs	63 (4.2)	240 (8.2)	135 (9.2)	49 (5.5)	635 (8.9)	461 (5.7)	1583 (7.2)
Proton pump inhibitors	41 (2.7)	612 (21.0)	197 (13.5)	133 (15.0)	1617 (22.6)	1143 (14.1)	3743 (17.0)
Drugs for urinary frequency and incontinence	9 (0.6)	40 (1.4)	32 (2.2)	2 (0.2)	79 (1.1)	110 (1.4)	272 (1.2)
Antihistamines	18 (1.2)	83 (2.9)	18 (1.2)	37 (4.2)	266 (3.7)	74 (0.9)	496 (2.3)

Data are presented as mean ± SD, n (%), or median [IQR]. The sign "—" indicates the corresponding variable is systematically missing.

BMI, body mass index; HADS-A, Hospital Anxiety Depression Scale-Anxiety subscale; MMSE, Mini-Mental State Examination; MET, Metabolic Equivalent of Task; eGFR, estimated glomerular filtration rate; CRP, C-reactive protein; ACE, angiotensin-converting enzyme; SSRI, selective serotonin reuptake inhibitor; TCA, tricyclic antidepressant; NSAID, nonsteroidal anti-inflammatory drug

[a]Defined by validated cutoff scores for Center for Epidemiological Studies Depression Scale (in LASA), Hospital Anxiety and Depression Scale-Depression subscale (in ActiFE Ulm), and Geriatric Depression Scale (in B-PROOF)

[b]Variable was harmonized across the three cohort studies and modelled using z-scores

[c]Variable was computed after multiple imputation

Supplementary Table 3. Performance of the AD*F*ICE_IT models for predicting any fall and recurrent falls based on internal-external cross-validation in the cohorts with prospective data on falls

	Validation in LASA[a]	Validation in B-PROOF[b]	Validation in ActiFE Ulm[c]
Model for predicting any fall			
C-statistic (95% CI)[d]	0.68 (0.64-0.71)	0.65 (0.63-0.67)	0.61 (0.58-0.64)
Intercept (95% CI)[e]	0.01 (-0.10-0.13)	-0.02 (-0.11-0.06)	0.02 (-0.10-0.14)
Slope (95% CI)[e]	1.12 (0.91-1.32)	0.79 (0.67-0.92)	0.67 (0.48-0.87)
Model for predicting recurrent falls			
C-statistic (95% CI)[d]	0.71 (0.67-0.76)	0.71 (0.67-0.74)	0.69 (0.64-0.73)
Intercept (95% CI)[e]	-0.03 (-0.20-0.14)	0.07 (-0.06-0.19)	0.11 (-0.07-0.29)
Slope (95% CI)[e]	1.01 (0.83-1.20)	0.79 (0.66-0.93)	0.88 (0.68-1.09)

[a] Models were derived from B-PROOF and ActiFE Ulm and then validated in LASA

[b] Models were derived from LASA and ActiFE Ulm and then validated in B-PROOF

[c] Models were derived from LASA and B-PROOF and then validated in ActiFE Ulm

[d] A C-statistic of 0.50 represents no discrimination and a C-statistic of 1.00 represents perfect discrimination

[e] Intercept of 0 and slope of 1 represent perfect calibration

Supplementary Figure 1: Calibration plots for the AD*F*ICE_IT models for predicting any fall (A) and recurrent falls (B) as derived from the internal-external cross-validation procedure in cohorts with prospective data on falls. Perfect calibration is represented by the dashed line.

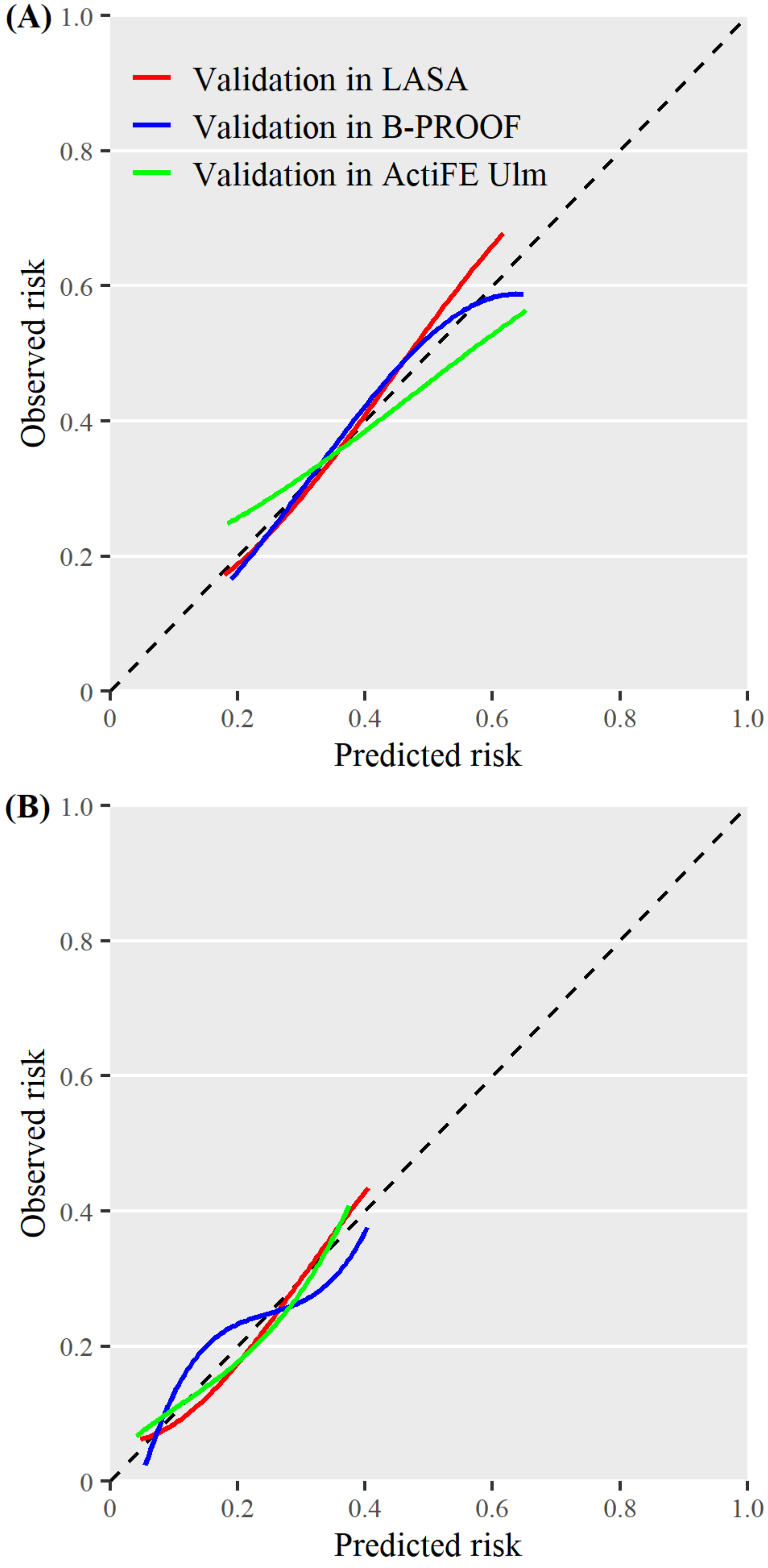

Supplementary Table 4. model for predicting any fall that was developed using a subset of the available candidate predictors in the cohorts with prospective data on falls

Predictor	Beta[a]	OR (95% CI)[a]
Intercept	-0.043	
Educational status		
Middle	0.173	1.19 (0.97-1.46)
High	0.324	1.38 (1.19-1.61)*
Depression score[b]	0.068	1.07 (1.00-1.15)
BMI	-0.018	0.98 (0.97-1.00)*
Number of functional limitations (0-5)[b]	0.125	1.13 (1.02-1.26)
Grip strength (kg)[b]	-0.148	0.86 (0.81-0.93)*
Gait speed (m/s)[b]	0.088	1.09 (1.01-1.18)
Systolic blood pressure (mmHg)	-0.003	1.00 (0.99-1.00)
≥ 1 fall in previous 12 months	0.426	1.53 (1.32-1.77)*
≥ 2 falls in previous 12 months	0.597	1.82 (1.49-2.21)*
Fear of falling		
Somewhat afraid of falling	0.199	1.22 (1.05-1.42)*
Very afraid of falling	0.195	1.22 (0.95-1.56)
Current smoker	-0.252	0.78 (0.64-0.95)*
Use of calcium channel blockers	-0.164	0.85 (0.71-1.02)
Use of antiepileptics	0.436	1.55 (1.00-2.40)
Use of drugs for urinary frequency and incontinence	0.656	1.93 (1.12-3.32)*

BMI, body mass index; CI, confidence interval

[a] Coefficients were corrected for overfitting with a shrinkage factor of 0.88

[b] OR refers to standardized Z-score, which were used for the purpose of harmonization. Z-scores are calculated as: Z-score $depression_{LASA}$ = (CES-D score – 7.980)/7.826; Z-score $depression_{B\text{-}PROOF}$ = (GDS score – 1.440)/ 1.942; Z-score $depression_{ActiFE\ Ulm}$ = (HADS-D score – 3.802)/2.899; Z-score functional $limitations_{LASA}$ = (number of functional limitations – 1.209)/1.529; Z-score functional $limitations_{ActiFE\ Ulm}$ = (number of functional limitations – 1.013)/1.362; Z-score grip $strength_{LASA}$ = (kg – 29.015)/10.244; Z-score grip $strength_{B\text{-}PROOF}$ = (kg – 32.484)/10.841; Z-score grip $strength_{ActiFE\ Ulm}$ = (kg – 33.364)/11.412; Z-score gait $speed_{LASA}$ = (s/m – 0.828)/0.287; Z-score gait $speed_{B\text{-}PROOF}$ = (s/m – 0.942)/0.276; Z-score gait $speed_{ActiFE\ Ulm}$ = (s/m – 1.035)/0.327

* $p < 0.01$

Supplementary Table 5. Model for predicting any fall that was developed using a subset of the available candidate predictors. Performance estimates were based on internal-external cross-validation in the cohorts with prospective data on falls

	Validation in LASA[a]	Validation in B-PROOF[b]	Validation in ActiFE Ulm[c]
C-statistic (95% CI)[d]	0.67 (0.64-0.70)	0.65 (0.63-0.67)	0.61 (0.58-0.65)
Intercept (95% CI)[e]	0.02 (-0.09-0.14)	-0.10 (-0.18- -0.02)	0.07 (-0.04-0.19)
Slope (95% CI)[e]	1.11 (0.90-1.32)	0.84 (0.69-0.99)	0.69 (0.50-0.89)

[a] Model was derived from B-PROOF and ActiFE Ulm and then validated in LASA

[b] Model was derived from LASA and ActiFE Ulm and then validated in B-PROOF

[c] Model was derived from LASA and B-PROOF and then validated in ActiFE Ulm

[d] A C-statistic of 0.50 represents no discrimination and a C-statistic of 1.00 represents perfect discrimination

[e] Intercept of 0 and slope of 1 represent perfect calibration

Supplementary Figure 2: Calibration plots for model for predicting any fall that was developed using a subset of the available candidate predictors. The calibration plots are based on internal-external cross-validation in the cohorts with prospective data on falls. Perfect calibration is represented by the dashed line.

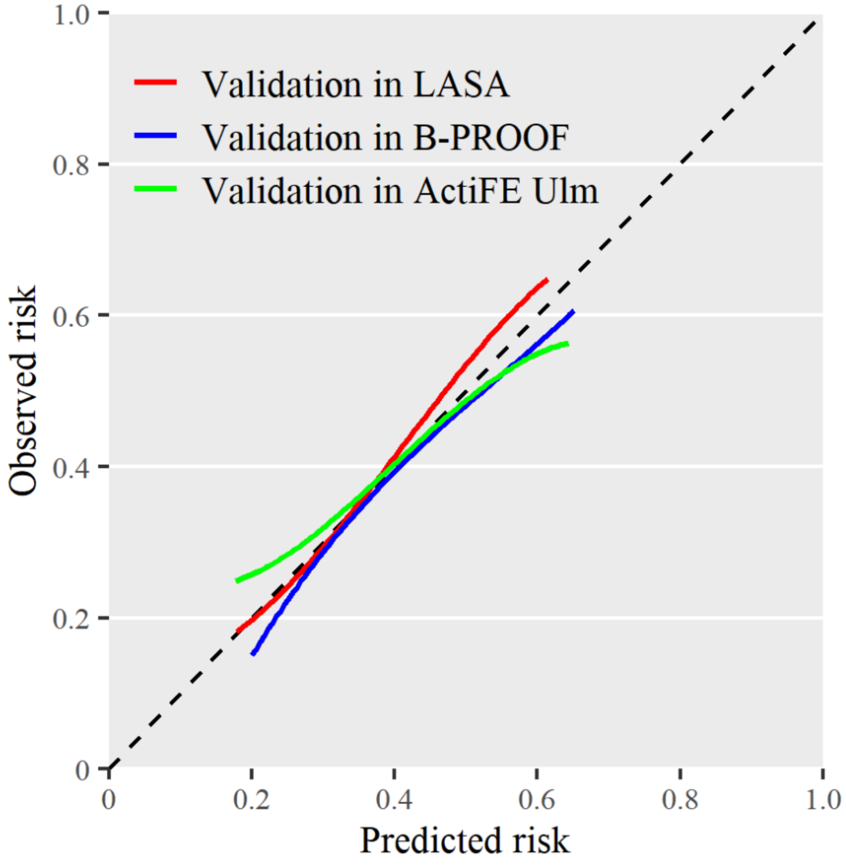

Supplementary Table 6. Regression coefficients for the prediction models for any fall as developed within user groups of selected medications in the cohorts with prospective data

Predictor[a]	Development population				
	Users of beta-blocking agents (*n* = 1406)	Users of low-ceiling diuretics (*n* = 891)	Users of statins (*n* = 1148)	Users of ACE inhibitor (*n* = 877)	Users of proton pump inhibitor (*n* = 832)
Intercept	-2.459	-0.563	-0.887	-1.392	-0.485
Age (years)	0.021	–	–	–	–
Education	–	–	–	–	–
Middle	0.052	–	–	0.115	–
High	0.536	–	–	0.582	–
Depression score[b]	–	–	–	–	0.142
Able to perform tandem stand for 10s	–	-0.378	-0.409	–	-0.439
Symptoms of pain	–	–	–	0.357	–
Poor self-rated health	–	–	0.499	–	–
Grip strength (kg)[b]	-0.208	–	–	–	–
≥ 1 fall in previous 12 months	0.482	–	0.585	0.471	0.529

≥ 2 falls in previous 12 months	0.678	1.269	0.913	0.787	0.718
Use of calcium channel blockers	-0.358	–	–	–	-0.620
Use of Alpha-adrenoceptor antagonist	–	–	–	–	-0.723
Use of antiepileptics	0.924	–	–	1.248	1.251
Use of opioids	–	–	–	0.890	–
Use of drugs for urinary frequency and incontinence	–	–	–	–	1.023
Use of antihistamines	–	–	0.970	–	–

ACE, angiotensin-converting enzyme

[a] Only predictors that were included in at least one of the models are shown

[b] OR refers to standardized Z-score, which were used for the purpose of harmonization. Z-scores are calculated as: Z-score grip strength$_{LASA}$ = (kg – 29.015)/10.244; Z-score grip strength$_{B\text{-}PROOF}$ = (kg – 32.484)/10.841; Z-score grip strength$_{ActiFE\ Ulm}$ = (kg – 33.364)/11.412; Z-score gait speed$_{LASA}$ = (m/s – 0.828)/0.287; Z-score gait speed$_{B\text{-}PROOF}$ = (m/s – 0.942)/0.276; Z-score gait speed$_{ActiFE\ Ulm}$ = (m/s – 1.035)/0.327

Supplementary Table 7. Performance of the prediction models for any fall as developed within user groups of selected medications in the cohorts with prospective data

User group	C-statistic for subgroup-specific model[a]	C-statistic for AD*F*ICE_IT model for predicting any fall[a]
Users of beta-blocking agents (*n* = 1406)	0.66	0.67
Users of low-ceiling diuretics (*n* = 891)	0.61	0.66
Users of statins (*n* = 1148)	0.66	0.68
Users of ACE inhibitors (*n* = 877)	0.66	0.66
Users of proton pump inhibitors (*n* = 832)	0.69	0.69

ACE, angiotensin-converting enzyme

[a] Optimism-adjusted C-statistic measures were obtained via a bootstrap procedure using 200 samples for each prediction model. A C-statistic of 0.50 represents no discrimination and a C-statistic of 1.00 represents perfect discrimination.

Supplementary Table 8. Final model for history of falls in community-dwelling older adults derived from the European studies with retrospective data on falls

Predictor	Beta[a]	OR (95% CI)
Intercept	-1.775	
Cohort		
LASA (wave 3B)	-0.214	0.81 (0.66-0.99)
B-PROOF	0.017	0.92 (0.79-1.08)
Rotterdam Study	-0.389	0.62 (0.54-0.71)*
ActiFE Ulm	-0.079	1.02 (0.86-1.21)
TILDA	-0.476	0.68 (0.59-0.78)*
Age (years)	0.009	1.01 (1.00-1.02)*
Sex, female	-0.131	0.88 (0.79-0.97)*
Education		
Middle	0.075	1.08 (0.98-1.18)
High	0.187	1.21 (1.11-1.32)*
Living with partner	-0.106	0.90 (0.83-0.97)*
Depression score[b]	0.041	1.04 (1-1.09)
Hearing impairment	0.212	1.24 (1.13-1.35)*
Symptoms of dizziness	0.174	1.19 (1.04-1.37)
BMI	-0.013	0.99 (0.98-1.00)*
Weight loss in past 3-6 months	0.218	1.24 (1.07-1.44)*
Able to perform tandem stand for 10s	-0.133	0.88 (0.79-0.97)*
Symptoms of pain	0.173	1.19 (1.09-1.3)*
Number of functional limitations (0-5)[b]	0.095	1.10 (1.05-1.15)*
Grip strength (kg)[b]	-0.111	0.90 (0.85-0.95)*
Urinary incontinence	0.341	1.41 (1.27-1.56)*
Fear of falling		
Somewhat afraid of falling	0.341	1.41 (1.29-1.53)*
Very afraid of falling	0.520	1.68 (1.42-1.99)*
Stroke ever	-0.151	0.86 (0.72-1.03)
Total physical activity (MET/week)[b]	0.053	1.05 (1.02-1.09)*
Alcohol use		
Drinks less than once a month	0.053	1.05 (0.93-1.20)
Drinks 1-3 times a month	0.128	1.14 (1.01-1.28)
Drinks 1-4 days a week	0.159	1.17 (1.06-1.30)*

Drinks (almost) daily	0.146	1.16 (1.04-1.30)*
Current smoker	-0.151	0.86 (0.78-0.95)*
eGFR (mL/min)	0.004	1.00 (1.00-1.01)*
Use of antiarrhythmics	0.298	1.35 (1.05-1.73)*
Use of antiadrenergic agents	-0.272	0.76 (0.57-1.02)
Use of low-ceiling diuretics	-0.085	0.92 (0.83-1.01)
Use of antidepressants	0.294	1.34 (1.18-1.53)*
Use of antiepileptics	0.203	1.23 (1.00-1.50)

BMI, body mass index; CI, confidence interval; MET, Metabolic Equivalent of Task; eGFR, estimated glomerular filtration rate

[a] Coefficients were corrected for overfitting with a shrinkage factor of 0.85

[b] OR refers to standardized Z-score, which were used for the purpose of harmonization. Z-scores are calculated as: Z-score depression$_{\text{LASA (wave C)}}$ = (CES-D score – 7.980)/ 7.826; Z-score depression$_{\text{LASA (wave 3B)}}$ = (CES-D score – 7.198)/7.008; Z-score depression$_{\text{B-PROOF}}$ = (GDS score – 1.440)/1.942; Z-score depression $_{\text{Rotterdam Study}}$ = (CES-D score – 5.689)/7.177; Z-score depression$_{\text{ActiFE Ulm}}$ = (HADS-D score – 3.802)/2.899; Z-score depression$_{\text{TILDA}}$ = (CES-D – 5.860)/7.226; Z-score functional limitations$_{\text{LASA (wave C)}}$ = (number of functional limitations – 1.209)/1.529; Z-score functional limitations$_{\text{LASA (wave 3B)}}$ = (number of functional limitations – 0.622)/1.181; Z-score functional limitations$_{\text{Rotterdam Study}}$ = (number of functional limitations – 1.530)/1.662; Z-score functional limitations$_{\text{ActiFE Ulm}}$ = (number of functional limitations – 1.013)/1.362; Z-score functional limitations$_{\text{TILDA}}$ = (number of functional limitations – 0.500)/0.903; Z-score grip strength$_{\text{LASA (wave C)}}$ = (kg – 29.015)/10.244; Z-score grip strength$_{\text{LASA (wave 3B)}}$ = (kg – 35.541)/12.311; Z-score grip strength$_{\text{B-PROOF}}$ = (kg – 32.484)/10.841; Z-score grip strength$_{\text{Rotterdam Study}}$ = (kg – 28.027)/10.322; Z-score grip strength$_{\text{ActiFE Ulm}}$ = (kg – 33.364)/11.412; Z-score grip strength$_{\text{TILDA}}$ = (kg – 28.052)/10.041; Z-score physical activity$_{\text{LASA (wave C and wave 3B), B-PROOF, Rotterdam Study, and ActiFE Ulm}}$ = (MET-min/week – 4005.135)/3059.858; Z-score physical activity$_{\text{TILDA}}$ = (MET-min/week – 2927.648)/3382.322

* $p < 0.01$

Supplementary Table 9. Performance of the model for history of a fall based on internal-external cross-validation in the cohort studies with retrospective data on falls

	Validation in LASA (wave C)[a]	Validation in LASA (wave 3B)[b]	Validation in B-PROOF[c]	Validation in the Rotterdam Study[d]	Validation in ActiFE Ulm[e]	Validation in TILDA[f]
C-statistic (95% CI)[g]	0.62 (0.58-0.65)	0.60 (0.55-0.65)	0.66 (0.63-0.68)	0.67 (0.65-0.69)	0.66 (0.63-0.69)	0.64 (0.63-0.66)
Intercept (95% CI)[h]	0.33 (0.21-0.44)	0.21 (0.05-0.36)	0.27 (0.17-0.37)	-0.39 (-0.46- -0.32)	0.39 (0.27-0.50)	-0.25 (-0.33- -0.16)
Slope (95% CI)[h]	0.65 (0.48-0.82)	0.65 (0.34-0.96)	0.90 (0.75-1.05)	0.97 (0.86-1.09)	0.98 (0.78-1.18)	0.93 (0.83-1.03)

[a] The model was developed using all studies except for LASA (wave C)

[b] The model was developed using all studies except for LASA (wave 3B)

[c] The model was developed using all studies except for B-PROOF

[d] The model was developed using all studies except for the Rotterdam Study

[e] The model was developed using all studies except for ActiFE Ulm

[f] The model was developed using all studies except for TILDA

[g] A C-statistic of 0.50 represents no discrimination and a C-statistic of 1.00 represents perfect discrimination

[h] Intercept of 0 and slope of 1 represent perfect calibration
